# Mass azithromycin distribution and antibiotic resistance in the gut and nasopharynx: a cluster-randomized trial

**DOI:** 10.1038/s41591-026-04217-9

**Published:** 2026-03-17

**Authors:** Thuy Doan, Daisy Yan, Ahmed M. Arzika, Amza Abdou, Ramatou Maliki, Bawa Aichatou, Ismael Mamane Bello, Diallo Beidi, Nasser Galo, Naser Harouna, Alio M. Karamba, Sani Mahamadou, Moustapha Abarchi, Almou Ibrahim, Lina Zhong, Cindi Chen, YuHeng Liu, Danny Yu, Thomas Abraham, Angela S. Cheng, Brittany Peterson, Catherine E. Oldenburg, Travis C. Porco, Benjamin F. Arnold, Armin Hinterwirth, Elodie Lebas, Kieran S. O’Brien, Thomas M. Lietman

**Affiliations:** 1 Francis I Proctor Foundation, University of California, San Francisco, CA USA; 2 Department of Ophthalmology, University of California, San Francisco, CA USA; 3 Centre de Recherche et Interventions en Santé Publique, Birni N’Gaoure, Niger; 4 Programme National de Santé Oculaire, Niamey, Niger; 5 Department of Epidemiology and Biostatistics, University of California, San Francisco, CA USA; 6 Institute of Global Health Sciences, University of California, San Francisco, CA USA

**Keywords:** Epidemiology, Developing world, Policy and public health in microbiology, Microbiome

## Abstract

Repeated semiannual azithromycin mass drug administration (MDA) to children has been shown to reduce all-cause childhood mortality. However, antibiotic resistance is a major public health concern as the program is being implemented in sub-Saharan Africa. In the double-blind, cluster-randomized, placebo-controlled trial (AVENIR) in Niger, we evaluated the impact of azithromycin MDA targeting different age groups on mortality and on the gut and nasopharyngeal microbiome and resistome of children in participating communities. A total of 3,000 communities were randomized in a 1:1:1 allocation to 3 arms: 2 years of semiannual MDA of (1: child–azithromycin) azithromycin to 1–59-month olds, (2: infant–azithromycin) azithromycin to 1–11-month olds and placebo to 12–59-month olds or (3: placebo) placebo to 1–59-month olds. Mortality (co-primary endpoint) and safety data have previously been published. Here we report on resistance (the co-primary endpoint). One hundred fifty communities (50 per arm) were selected for this analysis. A total of 4,382 rectal and 4,402 nasopharyngeal samples were included. The co-primary outcomes included changes in gut and nasopharynx macrolide AMR. The trial met its primary AMR endpoint for the gut but not for the nasopharynx. The gut macrolide AMR burden in fold change between arms was highest in child–azithromycin compared with placebo (1.16, 95% confidence interval (CI): 1.06–1.28; *P* < 0.01), followed by child–azithromycin compared with infant–azithromycin (1.13, 95% CI: 1.02–1.23; *P* = 0.01), and infant–azithromycin compared with placebo (1.04×, 95% CI: 0.94–1.15×; *P* = 0.66). There were no statistically significant differences in macrolide AMR selection fold change in the nasopharynx between arms: 2.14 (95% CI: 0.93–4.99) for child–azithromycin versus placebo, 2.08 (95% CI: 0.93–4.69) for infant–azithromycin versus placebo, and 1.03 (95% CI: 0.46–2.30) for child–azithromycin versus infant–azithromycin. Close monitoring of AMR should be an essential component of MDA for childhood mortality. ClinicalTrials.gov registration: NCT04224987

## Abstract (web summary)

In a double-blind, cluster-randomized, placebo-controlled trial in Niger that examined azithromycin mass drug distribution in different age groups of children, there was evidence of selection of macrolide resistance determinants in the gut in children 1 to 59 months old, but resistance to other classes of antibiotics were not observed in the gut or nasopharynx.

## Main

Mass drug distribution (MDA) of broad-spectrum antibiotics to preschool children prevents mortality of those under 5 years old in some regions in sub-Saharan Africa^1–4^. Currently, Niger, Nigeria, Burkina Faso and Mali are implementing large-scale programs distributing azithromycin every 6 months to all children aged 1–59 months. Other countries with similarly high mortality rates are considering the intervention. Targeting treatment to children aged 1–59 months was based on findings from AVENIR (Azithromycine pour la Vie des Enfants au Niger)^5^. Before this study, the World Health Organization’s (WHO) conditional guidelines in 2020 were to target children aged 1–11 months to reduce the risk of antimicrobial resistance (AMR) selection^6^. Given the challenging ethical issues involved, several additional trials were conducted after this time to provide extra data to help weigh the risks versus benefits before programs began on a larger scale, even though the guidelines were already available.

AVENIR was a double-blind, response-adaptive, cluster-randomized, placebo-controlled trial that demonstrated a 14% reduction in mortality only when azithromycin MDA was distributed to children aged 1–59 months. The same treatment for a narrower age group of 1–11 months, as recommended by WHO, did not result in a statistically significant decrease in mortality. In fact, treating the broader age range of 1–59 months resulted in a 17% lower mortality rate in the 1–11-month-old-age group compared to treating 1–11-month-old-age group alone^5^. In AVENIR, serious adverse events requiring hospitalization for malaria or vomiting, diarrhea and fever included three children in the placebo group, and one child and one infant in the azithromycin-treated group^5^.

A fundamental challenge for local stakeholders and policymakers has been how to best balance the mortality benefits of azithromycin MDA with the risk of AMR selection^7,8^. Here we report the AMR results of the AVENIR I trial, which was conducted after the expansion of seasonal malaria chemoprevention (SMC) that includes a long-acting sulfonamide, sulfadoxine^9^. We compared the gut and respiratory resistomes, two large reservoirs of AMR genes in the body, across three treatment arms. Azithromycin MDA modulations on the flora of these niches were also evaluated.

## Results

### Trial setting

**Fig. 1:**
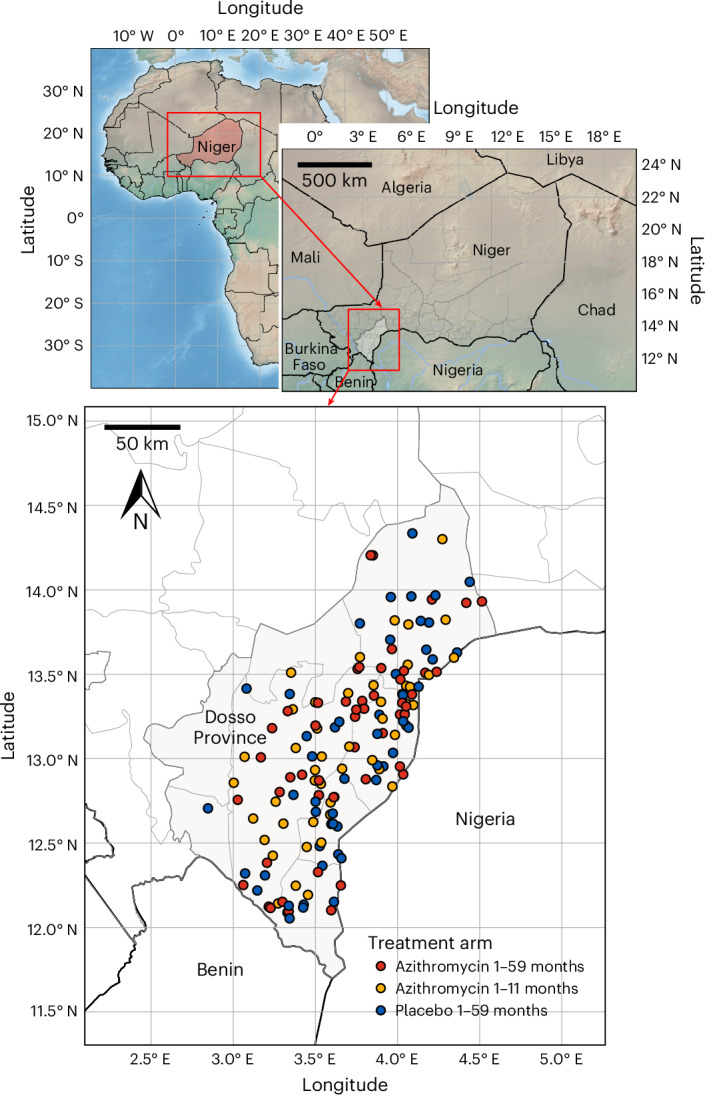
Regions in which communities were enrolled. Overview map of the Dosso Province in Niger and neighboring countries. Each point represents a community enrolled in the AMR trial (*n* = 150 communities). Niger administrative boundaries adapted from the Humanitarian Data Exchange under a CC BY 4.0 license (https://data.humdata.org/dataset/cod-ab-ner). Topography data from the European Space Agency (2024). Copernicus Global Digital Elevation Model. Distributed by OpenTopography (10.5069/G9028PQB). Continent-level and country-level maps made with Natural Earth (https://www.naturalearthdata.com/), accessed 25 April 2025. Source data

This AMR study was a cluster-randomized, placebo-controlled trial within the larger parent AVENIR I study^5^. Communities included in the AMR study were in the Dosso Province of Niger (Fig. 1). Since 2018, children aged 3–59 months in all communities in the study region received between 2 to 4 monthly distributions of SMC with sulfadoxine, pyrimethamine and amodiaquine during the malaria season. Therefore, this trial was conducted in a setting where programmatic SMC had been implemented for approximately 2 years.

### Trial participants

**Fig. 2:**
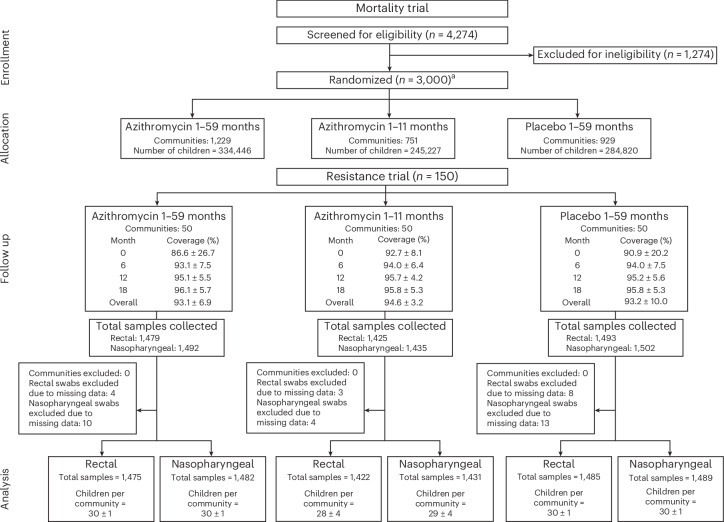
Study profile. The AVENIR study randomly assigned 3,000 rural communities in Niger to 4 semiannual distributions of azithromycin for children 1–59 months of age (child–azithromycin), 4 semiannual distributions for infants 1–11 months of age and placebo for children 12–59 months of age (infant–azithromycin), or placebo for children 1–59 months of age (placebo). All-cause mortality was significantly lower in the child–azithromycin arm compared to the placebo arm 5. A random sample of 150 communities was selected for AMR monitoring. Within each community, approximately 30 children were randomly chosen for rectal and nasopharyngeal sample collection at the 24-month follow-up time point for AMR analysis. ^a^After randomization, 91 communities were excluded, including 39 census inaccuracies, 7 refusals, 40 in insecure areas, 2 moved communities, 2 protocol deviations and 1 which only had 1 censused household. Source data

**Extended Data Fig. 1:**
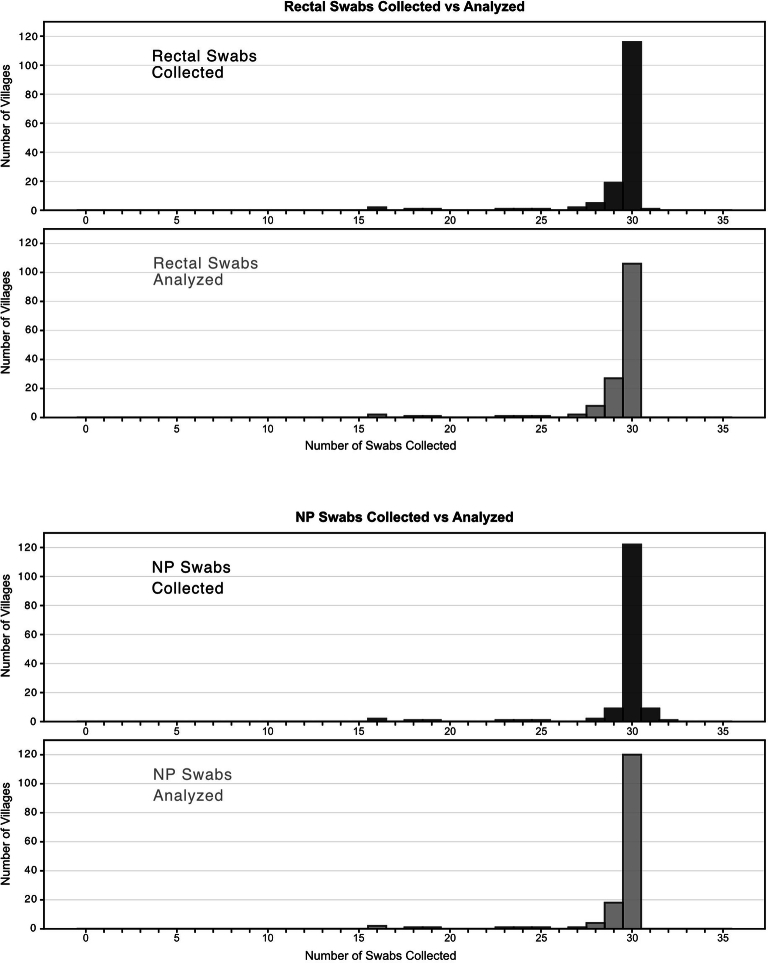
Sample Collection and Sample Analyzed Distributions. The number of swabs collected (top panels) and the number of swabs analyzed (lower panels) per community for rectal and nasopharyngeal categories. A total of 150 communities were included across the three treatment arms. Source data

The AVENIR I mortality trial randomized 3,000 villages to one of three arms: semiannual (every 6 months) azithromycin to participants 1–59 months old (child–azithromycin), semiannual oral azithromycin to 1–11 months old and placebo to 12–59 months old (infant–azithromycin), and semiannual placebo to 1–59 months old (placebo) for 2 years in Niger. From each arm, 50 villages were randomly selected to be included in the morbidity/AMR trial totaling 150 villages (Figs. 1 and 2). From each included village, 30 children aged 1–59 months were randomly selected for rectal and nasopharyngeal sample collection. A census was completed before to determine the sampling frame, and a simple random sample of eligible children was taken from the census data. The AMR study census was conducted from 24 November 2020 to 31 July 2023. Six months after the fourth semiannual MDA treatments (azithromycin or placebo), sample collection was obtained for the 24-month outcome AMR assessment, which started on 20 May 2023 and ended on 23 November 2023. Sampling was not stratified by age group. Any child aged 1–59 months was eligible for sample collection, regardless of having received a specific number of treatments. Study drug coverage over the four semiannual treatments was 93.1% ± 6.9% (± s.d.) for child–azithromycin, 94.6% ± 3.2% for infant–azithromycin and 93.2% ± 10.0% for placebo. No child was excluded due to azithromycin allergy. At the 24-month time point, an average of 29 ± 2 children per village provided rectal or nasopharyngeal samples (Table 1 and Extended Data Fig. 1). In total, 4,382 rectal samples and 4,402 nasopharyngeal samples were pooled, processed, sequenced and analyzed (Fig. 2 and Table 1). For the rectal samples, 1,475 samples from the child–azithromycin arm, 1,422 samples from the infant–azithromycin arm and 1,485 samples from the placebo arm were included in the 24-month analysis. For the nasopharyngeal samples, 1,482 samples, 1,431 samples and 1,489 samples were included in the child–azithromycin, infant–azithromycin and placebo arms, respectively. Characteristics of participants contributing the analyzed swabs are shown in Table 1.

**Table 1:** Demographics of analyzed participants

	Azithromycin 1–59 months	Azithromycin 1–11 months^a^	Placebo 1–59 months	Total
**Rectal**				
**Number of villages**	50	50	50	150
**Number of children**	1,475	1,422	1,485	4,382
**Number of children per village, mean (s.d.)** **Number of samples pooled per village, median (IQR)**	30 (1) 30 (30, 30)	28 (4) 30 (29, 30)	30 (1) 30 (30, 30)	29 (2) 30 (29, 30)
**Sex (%)**				
Male	735 (49.8%)	699 (49.2%)	777 (52.3%)	2,211 (50.5%)
Female	740 (50.2%)	723 (50.8%)	708 (47.7%)	2,171 (49.5%)
**Age, months**				
Median (IQR)	36 (22, 48)	34 (20, 48)	34 (20, 48)	35 (21, 48)
**Age category (%)**				
1–11 months	139 (9.4%)	153 (10.8%)	179 (12.1%)	471 (10.7%)
12–59 months	1,336 (90.6%)	1,269 (89.2%)	1,306 (87.9%)	3,911 (89.3%)
**Nasopharyngeal**				
**Number of villages**	50	50	50	150
**Number of children**	1,482	1,431	1,489	4,402
**Number of children per village, mean (s.d.)** **Number of samples pooled per village, median (IQR)**	30 (1) 30 (30, 30)	29 (4) 30 (29, 30)	30 (1) 30 (30, 30)	29 (2) 30 (30, 30)
**Sex (%)**				
Male	739 (49.9%)	707 (49.4%)	783 (52.6%)	2,229 (50.6%)
Female	743 (50.1%)	724 (50.6%)	706 (47.4%)	2,173 (49.4%)
**Age, months**				
Median (IQR)	36 (22, 48)	34 (20, 48)	34 (20, 48)	35 (21, 48)
**Age category (%)**				
1–11 months	140 (9.4%)	155 (10.8%)	180 (12.1%)	475 (10.8%)
12–59 months	1,342 (90.6%)	1,276 (89.2%)	1,309 (87.9%)	3,927 (89.2%)

Baseline characteristics of samples analyzed. IQR, interquartile range. ^a^Children aged 1–11 months were treated with azithromycin, while children aged 12–59 months were given placebo.

Source data

### Main study outcomes

The prespecified primary outcomes for this AMR study included separate analyses of the load of macrolide genetic resistance determinants in pooled community-level rectal and nasopharyngeal samples collected after four semiannual distributions. Analyses were performed hierarchically using a gatekeeping procedure to control for family-wise error rate to 5%, following this prespecified priority order: (1) child–azithromycin versus placebo, (2) infant–azithromycin versus placebo and (3) infant–azithromycin versus child–azithromycin. Antimicrobial resistance secondary outcomes included the load of genetic resistance determinants to nonmacrolide antibiotic classes for rectal and nasopharyngeal swabs. Phenotypic resistance, quantified by the proportion of macrolide-resistant pneumococcal isolates from nasopharyngeal swabs collected from children aged 1–59 months, was to be performed by the Niger National Reference Laboratory and is not reported here.

### Primary endpoints

**Fig. 3:**
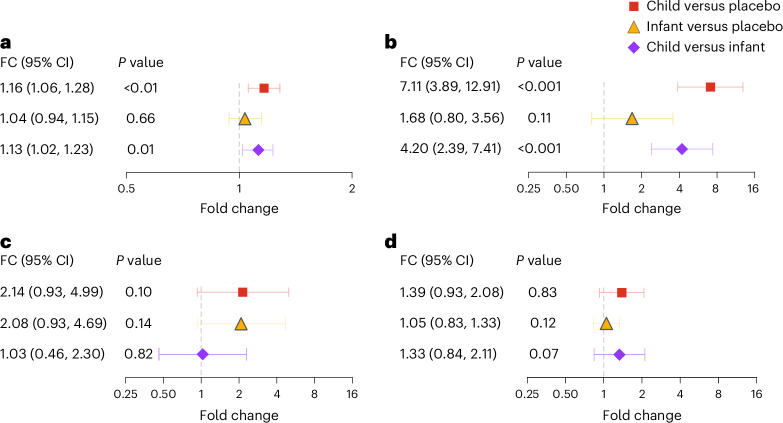
Macrolide AMR determinants between treatment groups. **a**, Change in gut macrolide AMR at class level for three comparison groups: child–azithromycin versus placebo (square); infant–azithromycin versus placebo (triangle) and child–azithromycin versus infant–azithromycin (diamond). **b**, Fold change in the antibiotic resistance gene *ermF* in the gut for the same comparison groups as in **a**. **c**, Change in nasopharyngeal macrolide AMR for the same comparison groups as in **a**. **d**, Fold change in *ermF* in the nasopharynx for the same comparison groups as in **c**. Bars indicate the mean and 95% CI derived from the *t* distribution. *P* values are unadjusted Wilcoxon rank-sum *P* values. Child–azithromycin: communities whose 1–59-month-old participants were treated with semiannual azithromycin (*n* = 50 communities); infant–azithromycin: communities whose 1–11-month-old participants were treated with semiannual azithromycin and 12–59-month-old participants were treated with placebo (*n* = 50 communities); placebo: communities whose 1–59-month-old participants were treated with semiannual placebo (*n* = 50 communities). FC, fold change. Source data

**Extended Data Fig. 2:**
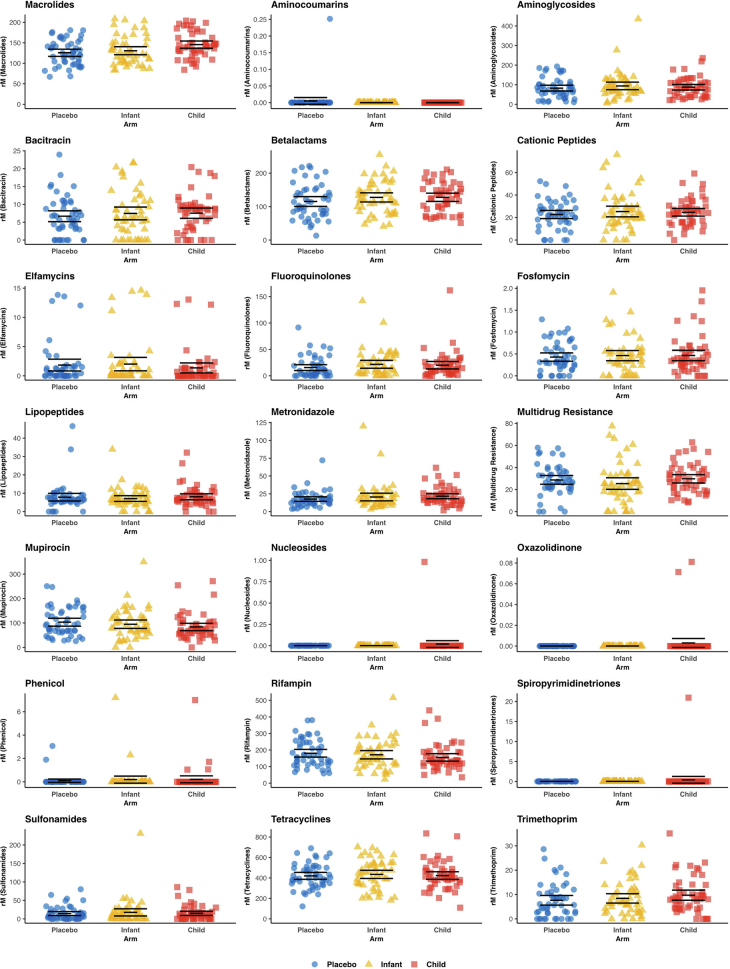
Normalized Gut Antibiotic-Resistance Determinants Across 3 Treatment Arms at the 24-Month Time Point. rM: matched resistance reads per million reads. Bars indicate median and 95% confidence intervals. Placebo: placebo distribution every 6 months to children 1–59 months (N = 50 communities); Infant: azithromycin distribution every 6 months to infants 1–11 months (N = 50 communities); Child: azithromycin distribution every 6 months to children 1–59 months (N = 50 communities). Source data

In the gut, macrolide genetic resistance at the class level was detected across all 150 communities at 24 months (6 months after 4 semiannual distributions) (Extended Data Fig. 2). Macrolide resistance determinants were 1.16-fold as high (95% confidence interval (CI): 1.06–1.28-fold, *P* < 0.01) in communities whose children aged 1–59 months were treated with azithromycin compared to communities whose children aged 1–59 months were treated with placebo (Fig. 3a). There were no detectable differences among the communities whose infants were treated only with azithromycin compared to communities whose children were treated with placebo (1.04-fold, 95% CI: 0.94–1.15, *P* = 0.66). However, communities whose children were treated with azithromycin had macrolide resistance determinants 1.13-fold higher (95% CI: 1.02–1.23-fold, *P* = 0.01) than communities where only infants were treated with azithromycin (Fig. 3a).

In the nasopharynx, macrolide resistance was not statistically significant between treatment groups (Fig. 3c). In the child–azithromycin group versus the placebo group, the mean fold difference in macrolide AMR was 2.14-fold (95% CI: 0.93–4.99-fold, *P* = 0.10; Fig. 3c). Similarly, the AMR difference in the infant–azithromycin arm compared to the placebo arm was 2.08-fold (95% CI: 0.93–4.69-fold), and 1.03-fold (95% CI: 0.46–2.30-fold) for the comparison between the child–azithromycin arm and the infant–azithromycin arm (Fig. 3c).

### Secondary endpoints

**Fig. 4:**
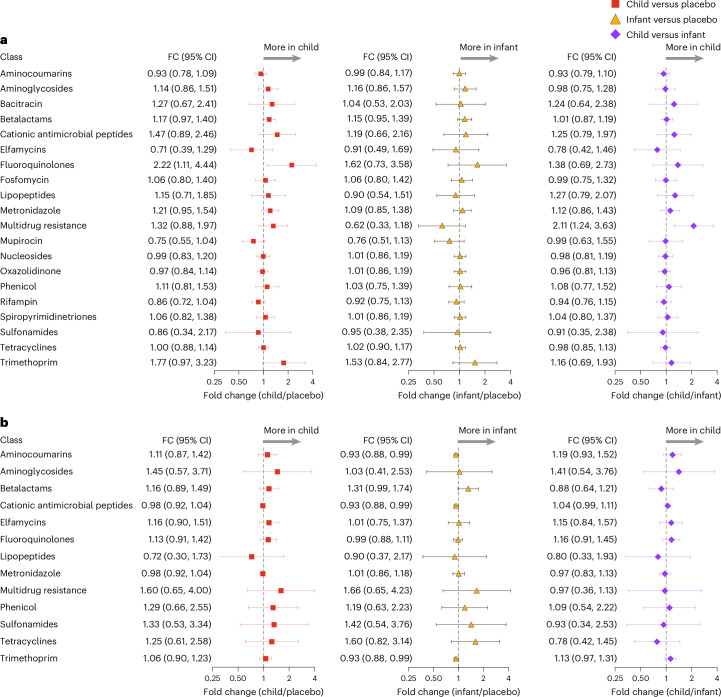
Nonmacrolide AMR determinants in the gut and nasopharynx between treatment groups. **a**,**b**, Fold change of nonmacrolide AMR in the gut (**a**) and nasopharynx (**b**) of children in three comparison groups: child–azithromycin versus placebo (square); infant–azithromycin versus placebo (triangle); child–azithromycin versus infant–azithromycin (diamond). Bars indicate the mean and 95% CI derived from the *t* distribution. Child–azithromycin: communities whose 1–59-month-old participants were treated with semiannual azithromycin (*n* = 50 communities); infant–azithromycin: communities whose 1–11-month-old participants were treated with semiannual azithromycin and 12–59-month-old participants were treated with placebo (*n* = 50 communities); placebo: communities whose 1–59-month-old participants were treated with semiannual placebo (*n* = 50 communities). Source data

**Extended Data Fig. 3:**
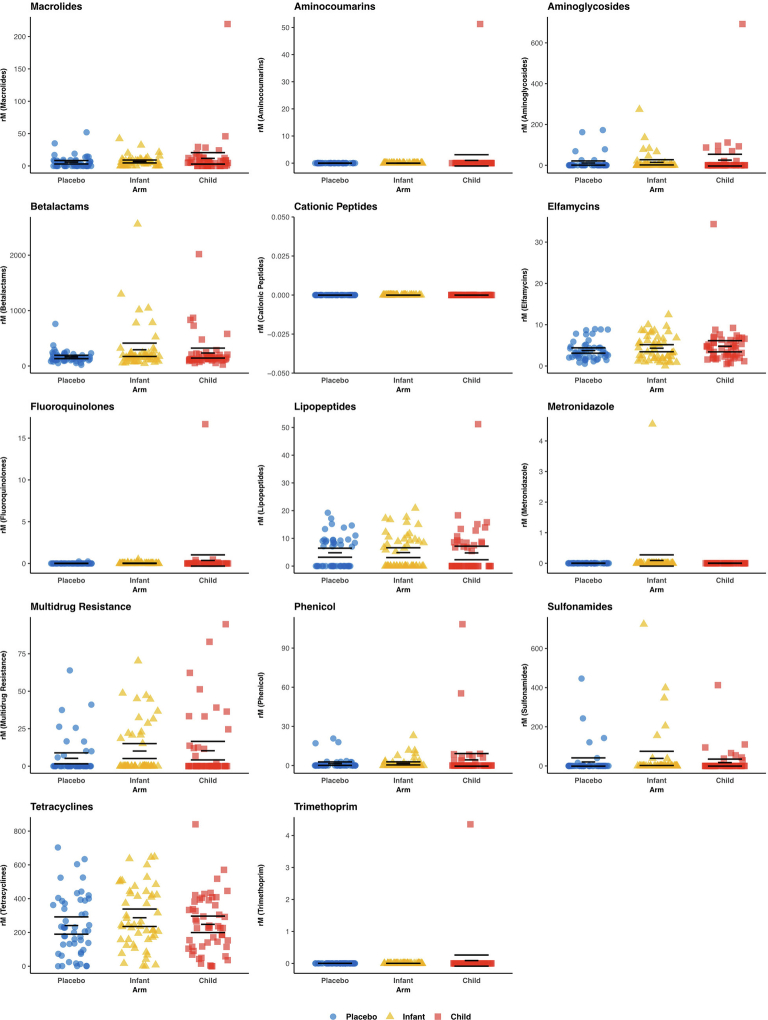
Normalized Nasopharyngeal Antibiotic-Resistance Determinants Across 3 Treatment Arms at the 24-Month Time Point. rM: matched resistance reads per million reads. Bars indicate median and 95% confidence intervals. Placebo: placebo distribution every 6 months to children 1–59 months (N = 50 communities); Infant: azithromycin distribution every 6 months to infants 1–11 months (N = 50 communities); Child: azithromycin distribution every 6 months to children 1–59 months (N = 50 communities). Source data

In the gut, 20 nonmacrolide antibiotic classes, including betalactams, were evaluated (Extended Data Fig. 2). There were no notable differences for any nonmacrolide antibiotic classes across all comparisons (child–azithromycin versus placebo, infant–azithromycin versus placebo and child–azithromycin versus infant–azithromycin) (Fig. 4a and Extended Data Table 1). Similar to the gut, there was no detectable selection for any of the nonmacrolide antibiotic classes evaluated in the nasopharynx (Fig. 4b, Extended Data Fig. 3 and Extended Data Table 2).

### Additional prespecified and exploratory outcomes

**Extended Data Fig. 4:**
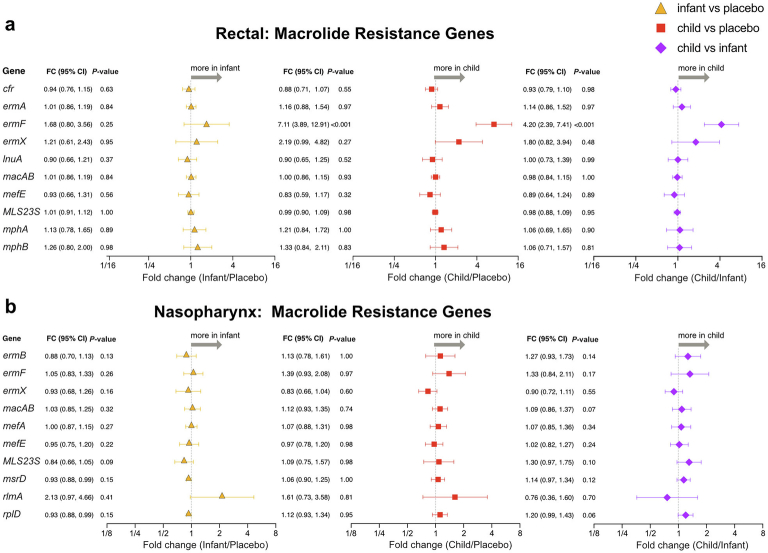
Macrolide Antibiotic Resistance Genes in the Gut and Nasopharynx in Infants and Children at 24 Months. The difference in antibiotic resistance genes (ARGs) for macrolides in the gut (**a**) and in the nasopharynx (**b**) in each of the 3 comparison arms. Bars represent mean and 95% confidence intervals. Child: azithromycin distribution every 6 months to children 1–59 months (N = 50 communities); Infant: azithromycin distribution every 6 months to infants 1–11 months (N = 50 communities); Placebo: placebo distribution every 6 months to children 1–59 months (N = 50 communities). Source data

**Extended Data Fig. 5:**
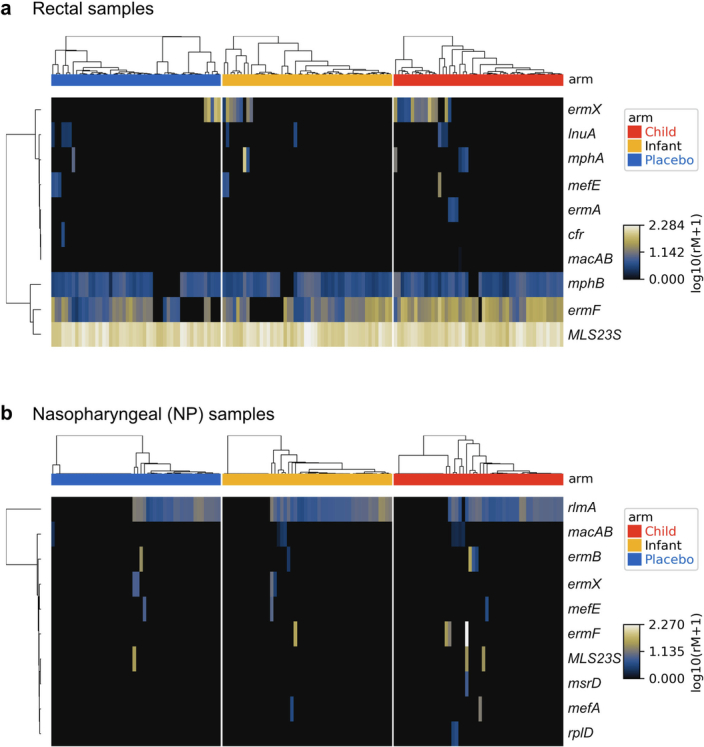
Clustered Image Maps of Gut and Nasopharyngeal Macrolide Antibiotic Resistance Genes (ARGs) Between Treatment Arms. Supervised hierarchical clustering using Ward’s method and Canberra distance over all detected macrolide ARGs, stratified by treatment arm for rectal (**a**) and nasopharyngeal (**b**) samples. Pooled samples (communities) are shown in columns and ARGs in rows. Heatmap colors represent relative ARG abundance (black = low, yellow = high). Child: azithromycin distribution every 6 months to children 1–59 months (N = 50 communities); Infant: azithromycin distribution every 6 months to infants 1–11 months (N = 50 communities); Placebo: placebo distribution every 6 months to children 1–59 months (N = 50 communities). Source data

Given that there was a difference in macrolide resistance between treatment groups in the gut, we next evaluated antimicrobial resistance genes (ARGs) to determine which ARGs may be associated with macrolide resistance at the class level. e*rmF*, an ARG that encodes a methyltransferase that modifies the 23S rRNA in the bacterial ribosome, drove the changes seen at the antibiotic class level in the gut (Fig. 3b, Extended Data Figs. 4a and 5a, and Extended Data Table 3). There was a 7.11-fold as high (95% CI: 3.89–12.91-fold, *P* < 0.001) in *ermF* in the child–azithromycin arm compared to the placebo arm and a 4.20-fold as high (95% CI: 2.39–7.41-fold, *P* < 0.001) in the child–azithromycin arm compared to the infant–azithromycin arm. Other detected macrolide ARGs were not different across the three treatment arms (Extended Data Figs. 4a and 5a). At the ARG level in the nasopharynx, neither *ermF* nor other macrolide resistance genes were selected for by azithromycin treatment in any of the comparison groups (Fig. 3d, Extended Data Figs. 4b and 5b, and Extended Data Table 4). The relative abundance of macrolide resistance in the gut did not correlate with the relative abundance of macrolide resistance in the nasopharynx across the three treatment arms (Spearman’s rank correlation, *r*_s_ = −0.11, *P* = 0.16). The overall ARG compositions, however, were also not different between treatment arms for the gut (Bray–Curtis PERMANOVA, *P* = 0.50) or for the nasopharyngeal (Bray–Curtis PERMANOVA, *P* = 0.39) resistomes.

**Fig. 5:**
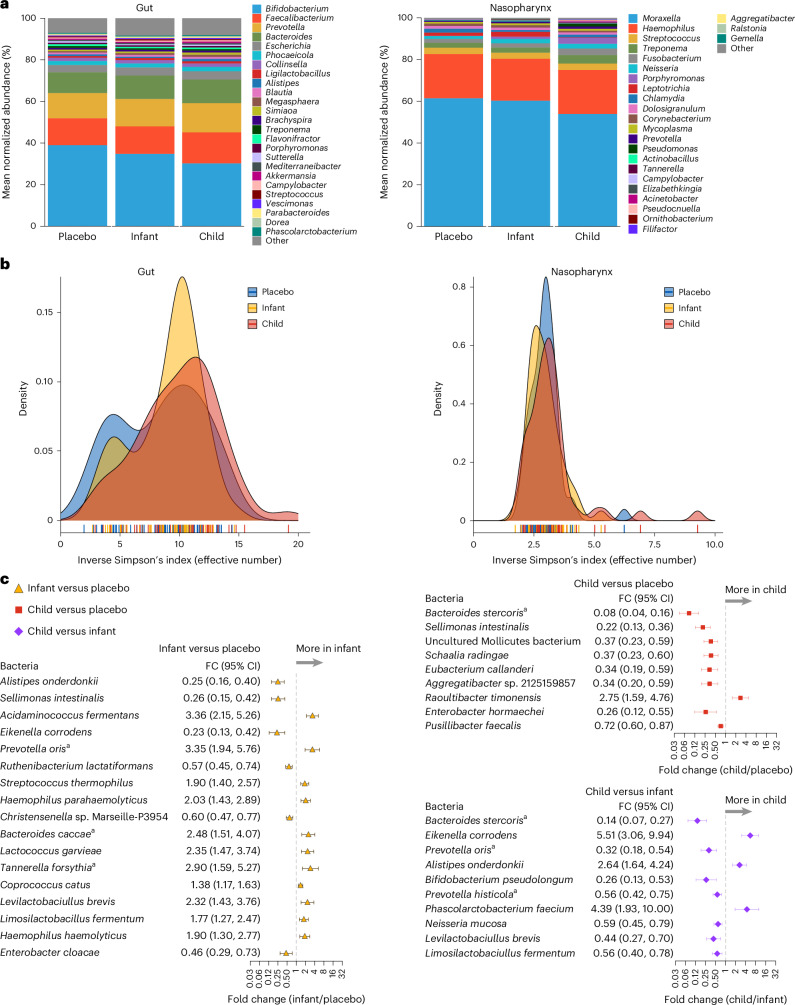
Gut and nasopharyngeal microbiomes of children in communities treated with semiannual placebo and azithromycin at 24 months. **a**, Microbiome composition with the top 25 genera for the gut and nasopharynx. **b**, Density distribution of the Simpson’s diversity indices for the gut and nasopharynx for the three treatment groups: child–azithromycin (red), infant–azithromycin (yellow) and placebo (blue). **c**, Notable differentially abundant bacterial taxa for three pairwise comparisons between treatment groups. Bars indicate mean and 95% CI derived from the Wald test. Notable differences were determined using the Benjamini–Hochberg false discovery rate <0.10 adjusted Wald test *P* values from DESeq2 and Topconfects. Child–azithromycin: communities whose 1–59-month-old participants were treated with semiannual azithromycin; infant–azithromycin (*n* = 50 communities): communities whose 1–11-month-old participants were treated with semiannual azithromycin and 12–59-month-old participants were treated with placebo (*n* = 50 communities); placebo: communities whose 1–59-month-old participants were treated with semiannual placebo (*n* = 50 communities). ^a^Bacterial species known to carry *ermF*. Source data

**Extended Data Fig. 6:**
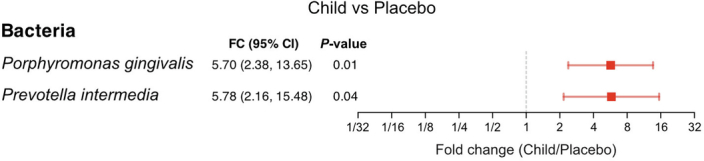
Relative Abundance of the Nasopharyngeal Microbiome Between Azithromycin- and Placebo-Treated Children Aged 1–59 Months. Top bacterial species at a 10% false discovery rate at 24 months. For each species, the dot shows the mean and 95% confidence interval. Benjamini-Hochberg correction was used to determine the adjusted P-values. No substantial differential abundances were observed for azithromycin-treated infants 1–11 months (N = 50 communities) vs. placebo (N = 50 communities) or azithromycin-treated children 1–59 months (N = 50 communities) vs azithromycin-treated infants 1–11 months. Source data

Both the gut and the nasopharyngeal microbiomes are reservoirs for antimicrobial resistance genes and are susceptible to antibiotic administration. In the Nigerien children, *Bifidobacterium*, *Faecalibacterium* and *Prevotella* were the three most abundant genera in the gut, while in the nasopharynx, *Moraxella*, *Haemophilus* and *Streptococcus* represented the three most abundant genera (Fig. 5a). The overall gut and nasopharyngeal microbiome structures were similar among treatment groups 6 months after the fourth drug distribution (Euclidean PERMANOVA, *P* = 0.49 for rectal samples; *P* = 0.10 for nasopharyngeal samples). Similarly, beta diversity using Bray–Curtis dissimilarity showed no differences in microbial composition at the species level between treatment arms for the gut (PERMANOVA, *P* = 0.62) and for the nasopharynx (PERMANOVA, *P* = 0.21). Semiannual azithromycin treatments in either children or infants for 2 years did not notably alter either the gut microbiome Shannon’s diversity index (*P* = 0.08, one-way ANOVA) or the inverse Simpson’s diversity index (*P* > 0.05) (Fig. 5b). In addition, there were no differences in alpha-diversity indices among the three arms for the nasopharyngeal samples (Shannon’s index, *P* = 0.35, one-way ANOVA; inverse Simpson’s index, *P* = 0.19) (Fig. 5b). However, differential abundance analysis of the gut micriobiome identified notable taxa between comparison arms (Fig. 5c). Among the differentially abundant taxa, *Acidaminococcus fermentans*, *Eikenella corrodens*, *Prevotella* spp. and *Bacteroides* spp. are documented to carry the *ermF* resistance gene^10–13^. In the nasopharynx, azithromycin-treated children had an increased relative abundance of *Porphyromonas gingivalis* (5.70-fold, 95% CI: 2.38–13.65-fold, *P* = 0.01) and *Prevotella intermedia* (5.78-fold, 95% CI: 2.16–15.48-fold, *P* = 0.04) compared to placebo-treated children (Extended Data Fig. 6). No differential nasopharyngeal taxa were identified for comparisons between the infant–azithromycin versus placebo arm or the infant–azithromycin versus child–azithromycin arm.

## Discussion

This cluster-randomized, masked, placebo-controlled trial supported the findings of prior cluster-randomized controlled trials in Niger, where macrolide resistance was increased with repeated azithromycin MDA^14–19^. Specifically, four semiannual azithromycin treatments to children aged 1–59 months led to a selection of macrolide resistance determinants in the gut. However, a difference in resistance genes to other classes of antibiotics was not observed in either the gut or the nasopharynx.

Azithromycin MDA in Niger has consistently shown a reduction in childhood mortality^1,3,5^. However, its effect may vary over time and geography. In the MORDOR trial (2015–2018), azithromycin MDA led to an 18.1% reduction in under-5 mortality, while in the AVENIR I mortality trial (2020–2023), the same azithromycin MDA led to a 13.9% reduction in under-5 mortality^1,5^. The observed differences in the effect of azithromycin MDA may be multifactorial. One reason is related to general secular trends of decreasing mortality in this setting and in nearby countries, such as Burkina Faso^4^. The second reason is that AVENIR I included a much wider geographic range than the prior MORDOR studies, which focused only on higher mortality districts in the Dosso region^1,3^. The AVENIR I mortality trial expanded to all of Dosso and then most of Tahoua, which has lower mortality rates^5^. Finally, AVENIR I was conducted when seasonal malaria chemoprevention had become routine. The widespread SMC distributions in the study region, covering both communities receiving placebo and those receiving azithromycin MDA, likely contribute to overall lower mortality rates and could even have modified the reduction in childhood mortality observed in AVENIR I, as compared to findings in MORDOR I in Niger^1–3,5^.

The relative reduction in the effect of azithromycin MDA on childhood mortality in AVENIR I paralleled the smaller fold difference in gut AMR observed between treatment arms. Prior AMR studies in the Dosso region of Niger showed a larger difference (2–7-fold) between communities receiving azithromycin MDA compared to placebo. Here, while the change was statistically significant, it was much more modest (16% higher in the child–azithromycin compared to placebo). This modest difference in macrolide AMR between treatment arms was likely a reflection of the higher background AMR, presumably due to broader antibiotic usage or access, and the increase in transmission of resistant organisms in these communities. Given that the last documented azithromycin MDA for trachoma in the Dosso region was in 2013, and no azithromycin MDA for childhood mortality was distributed in the study area before AVENIR I, programmatic azithromycin MDA was unlikely to predominantly drive the underlying increase in background MDA. In addition to the aforementioned factors affecting the reduction in childhood mortality, it is possible that spillover of AMR between study communities is also masking the true effect of AMR selection with azithromycin MDA in AVENIR I^20^.

Macrolide resistance in the gut of these Nigerien children was predominantly driven by an increase in *ermF* determinant abundance, an erythromycin ribosome methylation gene that was first described in colonic gram-negative anaerobic *Bacteroides* species and subsequently found in a wide range of clinical anaerobic and aerobic isolates^12,21–24^. While the overall structure of the gut microbiome was not statistically different between treatment arms, azithromycin MDA did result in specific alterations in the abundances of *Bacteroides* and *Prevotella* species, which potentially explained the changes in *ermF* detected. The prevalence of *ermF* ranges from 16% to 50% in clinical *Bacteroides* isolates in developed countries, and its presence is highly correlated with macrolide resistance^21,25^. In contrast, the prevalence of *ermF* in clinical *Prevotella* isolates is generally lower, and its presence is not consistently associated with macrolide resistance^11^. Although *Bacteroides* and *Prevotella* species are common commensal anaerobic bacteria of the gut, they are also opportunistic pathogens that can cause severe diseases in susceptible populations^26–28^. Furthermore, alterations in *Bacteroides* abundance and diversity in the gut are associated with dysbiosis^29,30^. The selection of *ermF* with azithromycin MDA has the potential to facilitate the persistence of resistant organisms within hosts to cause disease. While speculative, the potential for the spread of resistance to other organisms within and between hosts may reduce the efficacy of azithromycin MDA in communities undergoing repeated distributions.

Notably, we were unable to detect changes in *Escherichia coli*, considered an AMR reservoir in the gut and known recipients of *ermF* via horizontal transfer from *Bacteroides* and *Prevotella* species^21,31–34^. Similarly, other diarrheal-associated pathogens, such as *Salmonella* and *Campylobacter* species, were not different between treatment groups. These results suggest that infectious causes associated with mortality in the region may be changing, particularly when SMC has become routine^2,16^. Furthermore, the tracking of AMR may need to go beyond what can be easily cultured and phenotypically assessed. Both *Bacteroides* and *Prevotella* species are strict anaerobes, which generally require special handling and appropriate equipment to facilitate cultivation^35^. In countries where cold chain is inherently a challenge to maintain and the microbiology infrastructure is developing, unbiased testing of genetic materials in specimens at a centralized laboratory is a feasible approach to studying AMR. This approach, however, should not replace the appropriate phenotypic characterization coupled with the incorporation of newer technologies, such as long-read whole genome sequencing, to accurately surveil pathogen spread to guide future assessments in the setting of azithromycin MDA.

Previously we showed that a higher proportion of macrolide-resistant *Streptococcus pneumoniae* carriage was detected in preschool children whose communities were treated with four rounds of azithromycin MDA compared to those in communities treated with placebo^15^. Here we were unable to detect a significant change in macrolide resistance burden in the nasopharynx for any of the comparisons between treatment arms. In addition, the two most common AMR genes responsible for macrolide resistance in pneumococci, *ermB* and *mefA/E*, were not different between treatment arms. Consistent with this finding, microbiome analysis did not detect alterations in pneumococci with azithromycin MDA. Pathogen-directed cultivation of pneumococci, coupled with antibiotic sensitivity testing, may be necessary to demonstrate a difference. However, a recent phenotypic analysis of pneumococci isolated from children in communities treated with 6 doses of azithromycin MDA over 3 years did not reveal a statistically significant change in macrolide resistance prevalence compared to placebo^36^. These results are perhaps suggestive of an evolving temporal trend in respiratory disease-associated pathogens in the region. Here, *Porphyromonas gingivalis* and *Prevotella intermedia*, which have been shown to confer macrolide resistance in some clinical isolates, were found to be relatively enriched in communities treated with MDA compared to those treated with placebo^37–39^. Given that some of these organisms are associated with chronic periodontal disease and known to harbor *ermF* and other macrolide resistance genes, future studies may consider focusing on oral health and inflammation in the setting of MDA and childhood mortality^23,37,40^.

This study has multiple limitations inherent to simple, large, randomized controlled trials. Given that the participating rural communities were at considerable distances from the national laboratory, which prevented optimal sample handling for bacterial cultivation and phenotypic analysis, the trial relied heavily on unbiased genetic assays to quantify changes in AMR. While AMR determinants are often correlated with phenotypic resistance (for example, *ermF*), in the absence of minimum inhibitory concentration (MIC) assessment, the clinical impact on the host and the community is less clear^41,42^. The over-calling of AMR with molecular assays may result in guidelines that limit the programmatic reach of MDA for childhood mortality^43^. Thus, regional capacity building to facilitate accurate phenotypic monitoring should be an important component of any antibiotic MDA program. Indeed, phenotypic assessments of AMR in nasopharyngeal pneumococci are being conducted by Niger’s National Reference Laboratory in parallel with the molecular work done here. The cross-sectional sample collection of preschool children in the communities did not allow for insight into AMR virulence in pathogenic organisms, as our microbiome analysis was representative of changes in the colonizing organisms in the nasopharynx and the gut. The ongoing AVENIR II trial will address the potential of AMR causing disease in the setting of MDA by collecting samples from children with respiratory and gastrointestinal diseases who present to their local health posts. However, in the absence of longitudinal monitoring at the individual child-level, over the period of years and decades, latent changes in gut health and microbiome diversity, childhood development, and susceptibility to metabolic and autoimmune diseases related to repeated azithromycin MDA may escape detection^44–50^. Therefore, the improvement in childhood mortality at the present time has the potential trade-off for future deleterious health effects in an even wider susceptible population. Sample pooling at the village level, while economical for testing thousands of samples in this large-scale trial, prevented the accurate tracking of pathogenic bacterial strains between individual children^51^. An area of future work could examine changes in substitution rates within individual children via longitudinal samples using long-read sequencing platforms or combinations of short-read and long-read platforms^52–54^. Pooling also does not allow for other post hoc and subanalyses, in particular adjusting for or stratifying by age groups, or inference at the individual child-level. The inclusion of only children aged 1–59 months for sample collection and analysis may have led to the underestimation of overall AMR selection in the community, as children who received azithromycin MDA treatments already aged out of eligibility by the time sample collection was performed at 24 months. Aside from the antiparasitics and antibiotics included in SMC distributions for malaria since 2018, these communities did not undergo azithromycin MDA for childhood mortality before AVENIR I. As such, the changes in macrolide resistance observed in this study cannot be extrapolated to longer-term changes when additional MDA rounds are distributed or to regions where SMC distribution is not implemented. Of note, a large-scale AMR study of 150 mortality communities in MORDOR I–III showed that gut macrolide resistance in communities receiving 2 to 3 years of MDA was not significantly different than communities receiving a longer duration of MDA for 5 years^19^. That study, however, lacked a placebo arm for all 5 years, and AMR spillover among communities may have been a confounder and cannot be ruled out. The ongoing AVENIR II was designed to address the effect of spillover. Finally, despite the large sample included in the analysis, generalization of AMR results may be limited beyond rural communities in the Dosso region of Niger. Ongoing studies in the Resiliency through Azithromycin for Childhood Survival program are carefully surveilling AMR in Mali and Nigeria to better understand the regional effect of MDA.

In summary, several large community-randomized trials have found that semiannual administration of azithromycin to 1–59-month-old children in sub-Saharan Africa reduces childhood mortality. This study confirmed the selection of macrolide resistance and associated microbiome effects when azithromycin MDA is administered in regions concurrently receiving SMC. However, it was unable to demonstrate any co-selection of resistance to other classes of antibiotics, including betalactams, during the 6-month postadministration surveillance period. The benefits of decreased mortality in mass azithromycin programs will need to be balanced against the broad potential harm of increased macrolide-resistant bacteria, the progressive and generational loss of microbiome diversity, and other possible latent manifestations of neurodevelopmental and systemic diseases related to repeated MDA administrations in early childhood. The high likelihood of AMR spillovers from other regulated (for example, SMC) or unregulated (for example, livestock or over-the-counter dispensing through pharmacists and street vendors) uses of antibiotics at the regional level should be taken into consideration when implementing programmatic guidelines. Longitudinal and extended surveillance over a period of years, or even decades, may be necessary to establish the true societal benefit and cost of azithromycin MDA for childhood mortality. As programs are being rolled out in West African countries, extensive AMR surveillance is being embedded into program operations and decision-making to ensure the risk-benefit balance is monitored.

## Methods

### Ethics and inclusion

This trial was conducted according to Good Clinical Practice guidelines and adhered to the principles of the Declaration of Helsinki. We obtained ethical approval for the study from the University of California, San Francisco Committee for Human Research and the Comité National Éthique pour la Recherche en Santé in Niger. Before the start of the trial, the study team met in person with regional, district and community leaders to explain the nature of the study, answer questions and obtain verbal consent for the study communities to participate. This was done at all levels of the health system. Additional verbal consent was obtained from community leaders, and written informed consent was obtained from the guardians for sample collection, which was documented electronically. No incentives were provided for participation. A data and safety monitoring committee (DSMC) independently oversaw trial progress through annual meetings and quarterly progress reports.

### Study design

The AVENIR I trial was conducted from November 2020 to July 2023 in rural and peri-urban areas in the Dosso, Maradi and Tahoua regions in Niger (ClinicalTrials.gov registration: NCT04224987, registered 8 January 2020)^5^. AVENIR randomized 3,000 communities into 1 of 3 arms: 4 semiannual azithromycin distributions to participants aged 1–59 months old (child–azithromycin), 4 semiannual azithromycin distributions to participants aged 1–11 months old and placebo to participants aged 12–59 months old (infant–azithromycin) and 4 semiannual placebo distributions to participants aged 1–59 months old (placebo arm). The trial’s primary endpoints were mortality and macrolide antibiotic resistance. A random sample of 150 communities (50 per arm) from the Dosso region was included in the AMR study.

This study evaluated the direct effect of azithromycin MDA on AMR. Changes in macrolide AMR determinants at the class level in children 1–59 months of age were the prespecified main outcomes of this study. Secondary and exploratory outcomes included changes in nonmacrolide genetic resistance, microbiomes and AMR genes. Phenotypic resistance will not be reported here, as it is being investigated in parallel at Niger’s National Reference Laboratory, Centre de Recherches Médicales et Sanitaires (CERMES). Other prespecified primary outcomes include assessments for the spillover of macrolide AMR to other age groups, specifically 7–11 years, and guardians. Those results will be reported elsewhere when they are available. Details of the trial, including methods and all associated primary, secondary and exploratory outcomes, as well as protocol amendments and their dates, can be found in the protocol, as previously published, and are provided in the Protocol and Statistical Analysis Plan (SAP) sections of Supplementary Information^5^.

### Study setting, participants and eligibility criteria

The AMR study was conducted in the Dosso region of Niger. Only rural or peri-urban areas (population size between 250 and 2500) were included. Since 2018, children aged 3–59 months in communities in the study area received 2–4 monthly distributions of SMC with sulfadoxine, pyrimethamine and amodiaquine during the rainy season. The last trachoma MDA in any of the districts in the Dosso region was in 2013. The randomization unit was the grappe, which is the smallest administrative unit in Niger and approximately equivalent to a community.

### Community-level eligibility

#### Inclusion criteria

Inclusion criteria for communities were: located in Dosso (population of 250–2,499 as estimated from the most recent national census or projections), distance of more than 5 km from district headquarters town, distinguishable from neighboring communities and verbal consent from community leader(s).

#### Exclusion criteria

Exclusion criteria for communities were: inaccessible or unsafe for the study team and ‘quartier’ designation on the national census.

### Individual-level eligibility

#### Inclusion criteria

Inclusion criteria for individual participants were: age 1–59 months and weighing at least 3,000 g, primary residence in a study community and consent from their guardians.

#### Exclusion criteria

Exclusion criteria for individual participants were: children less than 1 month of age or older than 59 months, and those with known allergic reactions to macrolide antibiotics.

### Randomization and masking

The AVENIR trial used response-adaptive randomization at the community level^5^. AMR monitoring communities were randomly selected from the group that preceded response-adaptive allocation. The randomization unit was the grappe (community). The randomization sequence was generated by a biostatistician who was aware of the group assignments, using the R software (R Foundation for Statistical Computing). At the start of the trial, communities underwent randomization with equal probability (0.33) to one of the three groups:

1. Child–azithromycin: semiannual weight- or height-based dose of oral azithromycin suspension to children aged 1–59 months;
2. Infant–azithromycin: semiannual weight- or height-based dose of oral azithromycin suspension to children aged 1–11 months and oral placebo to children 12–59 months;
3. Placebo: semiannual weight- or height-based dose of oral placebo to children aged 1–59 months.

In the Dosso region of Niger, 150 communities (50 communities per arm) were randomly selected from the pool of eligible communities and followed to monitor AMR throughout the study. Aside from the UCSF biostatistician and data analyst responsible for the randomization, all other study personnel, participants, laboratory personnel and all field workers responsible for administering and sample collection were masked to treatment allocation. Allocation concealment was achieved by offering the treatment to all children in the community. The appearance and smell of the study drug were similar and packaged identically.

### Intervention

Children aged 1–59 months were identified in the door-to-door semiannual censuses. Interventions involved a single 20 mg kg^−^^1^ dose of oral azithromycin or placebo suspension offered to children aged 1–59 months at study months 0, 6, 12 and 18. Weight-based dosing was used for children aged 1–11 months, and height-based dosing was used for children 12–59 months. Children known to be allergic to macrolides were not treated.

### Sample collection

Of the communities in the Mortality Trial in the Dosso region, 150 were randomly selected for AMR monitoring. Sample collection for AMR monitoring at the 24-month time point started on 20 May 2023 and ended on 23 November 2023. In each community, 30 children aged 1–59 months were randomly chosen for sample collection at baseline and after 4 distributions. Rectal and nasopharyngeal samples stored in DNA/RNA Shield were processed as previously described^17,19^. Briefly, examiners changed into clean gloves before sample collection. Rectal samples were obtained by placing a swab (Zymo Research, flocked dry swabs with 80 mm breakpoint) 1–3 cm into the anus of each participant, rotating 360 degrees, and repeating until stool was visible on the swab and placed in DNA/RNA Shield. Nasopharyngeal swabs were obtained by placing a pediatric flocked swab with a nylon tip through the right nostril and down the nasopharynx of each participant, rotating the swab by 180 degrees as the swab was removed from the nose, and storing it in DNA/RNA Shield. In addition, a random sample of 10 children aged 1–59 months per community were selected for nasopharyngeal swab collection, which was stored in STGG for phenotypic analysis at Centre de Recherches Médicales et Sanitaires. The phenotypic results will be reported by the center when available. This study focused on the genetic resistance and microbiome-related results from samples collected and stored in nucleic acid preserving media.

### DNA-Seq, resistome and microbiome assessments

Rectal and nasopharyngeal samples stored in DNA/RNA Shield were processed as previously described^17,19^. All verified rectal and nasopharyngeal samples from each village were pooled at the village level, respectively. This resulted in 150 pooled rectal samples and 150 pooled nasopharyngeal samples. After pooling, nucleic acids were extracted, processed for DNA-seq and sequenced. Sequencing libraries were performed using the NEBNext Ultra II DNA Library Prep Kit (New England Biolabs) per manufacturer’s recommendations. Sequencing libraries were amplified with 10 PCR cycles for rectal samples and 14 PCR cycles for nasopharyngeal samples. Libraries were quantified, pooled and sequenced on the NovaSeq X (Illumina) using 150-nt paired-end sequencing. Given nasopharyngeal samples were low biomass, the sequencing library was subjected to another round of sample processing to enrich for antimicrobial resistance genes. Briefly, multiplexed DNA libraries were pooled in equimolar amounts. Pooled libraries were enriched for AMR reads using the KAPA HyperExplore probes (Roche) designed to enrich for 3,623 AMR genes (obtained from the Comprehensive Antibiotic Resistance Database and ResFinder)^55–57^. Pooled libraries were prepared for hybridization using the KAPA HyperCapture Reagent Kit (Roche). Libraries were hybridized to the enrichment probes for 20 h, and hybridized libraries were recovered using the KAPA HyperCapture Beads (Roche). Bead-bound hybridized libraries were washed, then underwent 20 cycles of postcapture PCR amplification, cleaned with KAPA HyperCapture Beads, pooled and sequenced on the NovaSeq X.

Paired-end sequencing reads were subjected to two rounds of human sequencing read removal using the human reference genome 38 (hg38) and the *Pantroglodytes* genome (panTro4, 2011, UCSC). For nasopharyngeal samples, nonhost sequencing reads obtained from the nonenriched and AMR-enriched libraries were combined for resistome analysis. The remaining nonhost reads were quality filtered and aligned to the MEGARes reference antimicrobial database (version 3.0) that included over 8,000 antimicrobial resistance gene accessions using the Burrows–Wheeler Aligner with default settings^58,59^. Antibiotic resistance determinants were included if they met 80% nucleotide identity between the query sequence and the reference AMR sequence. Any hits that failed SNP confirmation (for determinants that require it) were excluded. Each identified AMR determinant was classified at the class level and gene level using Resistome Analyzer^59^. If there were 0 hits across all samples, the respective class or gene was excluded from analysis. Kraken2 (version 2.1.2) was used for taxonomic classification for microbiome assessment for both rectal and nasopharyngeal samples, and Bracken (version 2.5) was used for estimation of genus and species abundances^60–62^.

### Sample size

For load of genetic resistance determinants in nasopharyngeal swabs, we estimated that 50 communities per group would provide 80% power to detect an approximately 2-fold difference in any pairwise comparison, assuming an alpha of 0.05 and a s.d. of the log base 2 read count of 1.88. The s.d. of the log read count was derived from the placebo arm of a previous cohort of children in Burkina Faso. For load of genetic resistance determinants in rectal swabs, the inclusion of 50 communities per group provided 80% power to detect a 2.6-fold difference in bacterial reads with resistance determinants between any two arms, assuming a s.d. of 2.4 on the log scale^15^.

### Statistical methods

All analyses specified were replicated for both rectal swabs and nasopharyngeal swabs. Normalized reads (counts per million) at the antibiotic class and AMR gene level were used for all analyses except for the microbiome analysis, where non-normalized reads at the bacteria species level were used. Antibiotic class level reads were derived from a summation of reads from all corresponding AMR genes. Before analysis, normalized reads were transformed using log_2_(*x* + 1/nonhost reads) to account for differences in sequencing depth across pooled samples for each community. To obtain fold-change difference values, the mean difference of log-transformed normalized reads was exponentiated. As the trial was conducted at the community level and all analyses targeted community effects, both sexes were included in all analyses. Data were not reported disaggregated by sex. Analyses relevant to this AMR study in the SAP were amended on 25 September 2024, to increase the sample size from 60 to 150 communities and on 27 May 2025, to clarify AMR outcome analyses (SAP in Supplementary Information). All statistical analyses were conducted in R (R Foundation for Statistical Computing, version 4.3.1).

#### Prespecified primary outcomes

For macrolide resistance determinants at the class level, pairwise Wilcoxon rank-sum tests were performed between each treatment arm. The analysis followed a fixed-sequence, hierarchical testing approach to adjust for family-wise error rate, and the results were presented in the same order: (1) child–azithromycin versus placebo, (2) infant–azithromycin versus placebo and (3) child–azithromycin versus infant–azithromycin. If a pairwise comparison was not significant with an alpha of 0.05, then subsequent comparison *P* values were not reported. We reported the fold change for each comparison, defined as the difference in means of transformed and normalized reads, along with 95% CI derived from the two-sample *t*-test and two-sided Wilcoxon *P* values.

#### Prespecified secondary outcomes

Pairwise Wilcoxon rank-sum tests were also performed for all other nonmacrolide resistance determinants at the class level (for example, betalactams), accounting for multiple comparisons using the Benjamini–Hochberg false discovery rate (FDR) adjustment with a prespecified FDR < 0.05 (ref. ^19^). To capture differences in gut and nasopharynx microbial community structures across treatment arms, permutational analysis of variance analysis (PERMANOVA) was performed to assess differences in within-group and between-group Manhattan and Euclidean distances^63^. Shannon’s Index and inverse Simpson’s Index were computed at the community level using a one-way ANOVA test^63^. Differential abundance analysis across species was performed between arms using DESeq2, Topconfects and an FDR < 0.10 (refs. ^64,65^).

#### Post hoc and exploratory outcomes

Beta diversity of microbial communities and ARGs across treatment groups was assessed using Bray–Curtis and Jaccard distances. Notable differences were evaluated by a permutation test with 10,000 permutations. Differential abundance analysis across ARGs was performed between arms using DESeq2, Topconfects and an FDR < 0.05 (refs. ^64,65^).

### Reporting summary

Further information on research design is available in the Nature Portfolio Reporting Summary linked to this article.

## Online content

Any methods, additional references, Nature Portfolio reporting summaries, source data, extended data, supplementary information, acknowledgements, peer review information; details of author contributions and competing interests; and statements of data and code availability are available at 10.1038/s41591-026-04217-9.

## Supplementary information

Supplementary Information AVENIR personnel, protocol and SAP.

Reporting Summary

## Source data

Source Data Table 1 Detailed demographics and pooling information about each sample used in the study.

Source Data Fig. 1 Geographic information of study villages. Precise GPS locations redacted for privacy reasons.

Source Data Fig. 2 Detailed information about individual samples used for pooling.

Source Data Fig. 3 Normalized count values (rM) of ARGs for gut and nasopharyngeal samples.

Source Data Fig. 4 Normalized count values (rM) of nonmacrolide AMR classes for gut and nasopharyngeal samples.

Source Data Fig. 5 Microbiome data for gut and nasopharyngeal samples. (For each sample type: kraken2 + bracken raw counts, normalized (rM) bacterial counts and metadata.)

Source Data Extended Data Fig. 1 Number of swabs collected and analyzed for each sample pool, for rectal and nasopharyngeal samples.

Source Data Extended Data Fig. 2 Raw counts of AMR determinants for all pooled rectal samples, as well as the number of input reads for normalization, and metadata with treatment groups.

Source Data Extended Data Fig. 3 Raw counts of AMR determinants for all nasopharyngeal samples, as well as the number of input reads for normalization, and metadata with treatment groups.

Source Data Extended Data Fig. 4 Gut and nasopharyngeal ARG counts in reads-per-million (rM).

Source Data Extended Data Fig. 5 Normalized count data for macrolide antibiotic resistance genes (ARGs) for all samples (rectal and NP), with treatment groups.

Source Data Extended Data Fig. 6 Kraken2/bracken derived raw counts at species and genus level for all samples, as well as the number of input reads for normalization.

Source Data Extended Data Table 1 Gut nonmacrolide AMR class counts, reads-per-million (rM) and table with full ANOVA results.

Source Data Extended Data Table 2 Nasopharyngeal nonmacrolide AMR class counts, reads-per-million (rM) and table with full ANOVA results.

Source Data Extended Data Table 3 Gut macrolide ARG counts, reads-per-million (rM) and table with full ANOVA results.

Source Data Extended Data Table 4 Nasopharyngeal macrolide ARG counts, reads-per-million (rM) and table with full ANOVA results.

## Figures and Tables

**Extended Data Table 1:**
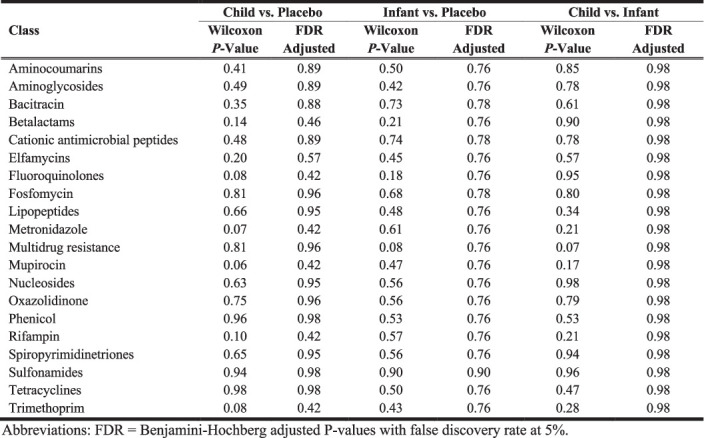
Gut Non-Macrolide Antibiotic Resistance Determinants for Comparisons Between Treatment Groups

**Extended Data Table 2:**
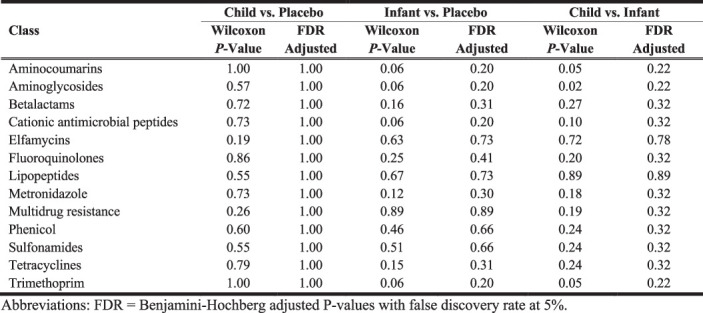
Nasopharynx Non-Macrolide Antibiotic Resistance Determinants for Comparisons Between Treatment Groups

**Extended Data Table 3:**
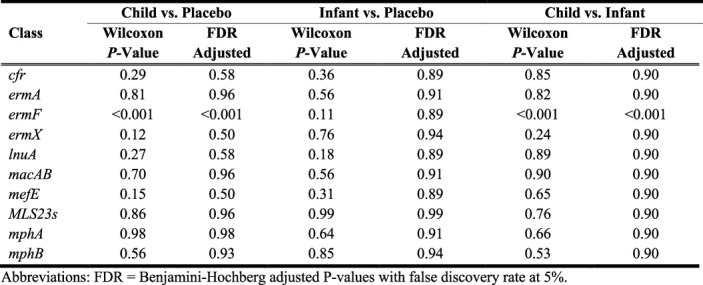
Gut Macrolide Antibiotic Resistance Genes for Comparisons Between Treatment Groups

**Extended Data Table 4:**
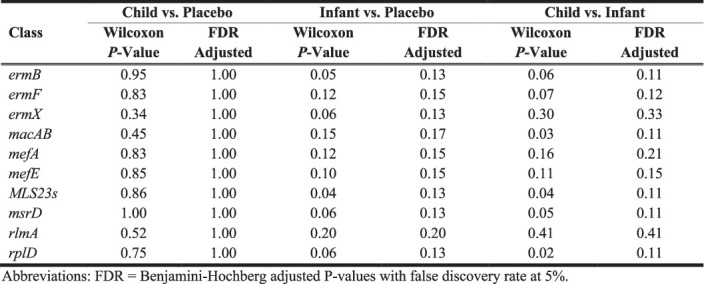
Nasopharynx Macrolide Antibiotic Resistance Genes for Comparisons Between Treatment Groups

## Data Availability

Nonhost sequencing reads for all pooled samples are available at BioProject ID PRJNA1337442. Limited deidentified individual information is available via Dryad at 10.5061/dryad.p8cz8wb48 (ref. ^66^). Requests for more information, beyond the scope of the reported results in this article, are subject to approval by the AVENIR Study Group and must comply with legal and regulatory requirements. Requests can be made to the PIs, Tom.Lietman@ucsf.edu and/or Kieran.Obrien@ucsf.edu, and will be addressed within 120 days. A data transfer agreement may be required. Source data are provided with this paper.
