# Supplementary material for: Mass azithromycin distribution and antibiotic resistance in the gut and nasopharynx: a cluster-randomized trial
Source: Nat Med. 2026 Mar 17;32(3):859–68. doi: 10.1038/s41591-026-04217-9 (PMC13004693; doi:10.1038/s41591-026-04217-9)
Supplement: Supplementary file 1 — AVENIR personnel, protocol and SAP. [file 41591_2026_4217_MOESM1_ESM.pdf]

nature medicine

Supplementary information https://doi.org/10.1038/s41591-026-04217-9

Mass azithromycin distribution and antibiotic resistance in the gut and nasopharynx: a cluster-randomized trial

In the format provided by the authors and unedited

AVENIR Study Group, Collaborators, Investigators, and Study Personnel

Centre de recherche et interventions en santé publique, Niamey, Niger – Ahmed Arzika, Ramatou Maliki, Bawa Aichatou, Ismael Mamane Bello, Ousseini Boubacar, Nasser Gallo, Karamatoulaye Hamadou, Amadou Harouna, Naser Harouna, Laminou Maliki Haroun, Alio Karamba, Mariama Keimago, Sani Mahamadou, Abarchi Moustapha, Abraham Omar, Farissatou Oumarou

Programme national de santé oculaire, Niamey, Niger – Amza Abdou, Ibrahim Almou, Nassirou Beido, Nouhou Diori Adam, Hamidou Amadou Bagna, Hassane Amadou Traore, Boubacar Kadri, Boubacar Maïdanda, Boubacar Nameywa, Boubacar Mariama, Abba Kaka Hadjia Yakoura, Youssoufou Souley Abdoul Salam

University of California, San Francisco, San Francisco, CA, USA – Benjamin Arnold, Carolyn Brandt, Cindi Chen, Emily Colby, Catherine Cook, Thuy Doan, Jeremy D Keenan, Sandrine Kyane, Victoria Le, Elodie Lebas, Thomas M Lietman, Zijun Liu, William Nguyen, Kieran S O'Brien, Catherine E Oldenburg, Brittany Peterson, Travis C Porco, George W Rutherford, Lina Zhong, Zhaoxia Zhou

Other Study Partners and Collaborators

Bill & Melinda Gates Foundation, Seattle, WA, USA – Rebecca Brander, Dennis Chao, James Heine, Rasa Izadnegahdar, Laura Lamberti, Assaf Oron, Surabhi Rajaram, Matthew Steele

Centre de recherche médical et sanitaire, Niamey, Niger – Ibrahima Issa, Ronan Jambou, Rabiou Labbo, S'Hooshim N. Lamine, Sani Ousmane, Boubakar Rakia, Maikano Sadikou

Centre de santé communautaire, Niamey, Niger – Amadou Zakari

Direction des statistics, Niamey, Niger – Aida Mounkala, Salamatou Maiga, Mahamadou Yahaya

Centre de santé de la mère et de l'enfant, Dosso, Niger – Amina Seyfoulaye

ClinEpiDB, Philadelphia, PA, USA – Brianna Lindsay, Nupur Kittur, David Roos, Sheena Tomko

Comité national éthique pour la recherche en santé, Niamey, Niger – Issa Adji, Djibo Ali, Souleymane Alzouma, Maidanda Boubacar, Diegou Boureima, Cheik Boureima Daouda, Idi Moussa Djatao, Ibrahim Jean Etienne, El Hadji Boubakar H Maiga, Amadou Oumarou, Ocquet Sakina, Sanoussi Samuila

Data and Safety Monitoring Committee – *Emory University, Task Force for Global Health* – David Addiss (chair); *Niger Ministry of Health* – Mourtala Assao; *St. George's University of*

London – Julia Bielicki, *Retired from CDC* – Allen Hightower, *Children's Hospital of Colorado* – Brian Jackson, *University of Melbourne* – Fiona Russell

Global Health Strategies, New York, NY, USA – Frances Hocking, Zied Mhirsi, Dan Pawson

International Trachoma Initiative **–** Paul Emerson, PJ Hooper

Johns Hopkins University, Baltimore, MA, USA – Agbessi Amouzou, Robert Black

Parasites Without Borders, USA – *Charles Knirsch*

Speak Up Africa, Dakar, Senegal – Maelle Ba, Yaye Sophiétou Diop, Yacine Djibo, Gráinne Hutton, Fara Ndiaye

Tampere University, Tampere, Finland **–** *Per Ashorn, Ulla Ashorn*
UCSF-UC Berkeley Center for Global Health Development, Diplomacy, and Economics, CA, USA – Stefano M. Bertozzi, Jim G. Khan, Elliot Marseille

University of Maryland School of Medicine, **Center for Vaccine Development and Global Health*, Baltimore, USA*** – Meagan Fitzpatrick

Azithromycine pour la Vie des Enfants au Niger: Implémentation et Recherche (Azithromycin for Child Survival in Niger: Implementation and Research: AVENIR) Trial Protocol

An adaptive cluster-randomized trial to determine the optimal age group for implementation of biannual oral azithromycin distribution to reduce child mortality in Niger

Investigators and Study Personnel (in alphabetical order):

Eric Adehossi[3,4]
Abdou Amza*[3,4]
Benjamin Arnold[1]
Ahmed Arzika*[2]
Nassirou Beido[3,4]
Emily Colby[1]
Adam Diori[3,4]
Thuy Doan[1]
Boubacar Kadri[3,4]
Jeremy D. Keenan[1]
Sandrine Kyane[1]
Elodie Lebas[1]
Thomas M. Lietman*[1]
Zijun Liu[1]
Boubacar Maidanda[3,4]
Ramatou Maliki[4]
Aissa Mamadou[3,4]
Kamayé Moumouni[3,4]
William Nguyen[1]
Kieran S. O'Brien*[1]
Catherine E. Oldenburg[1]
Brittany Peterson[1]
Travis C. Porco[1]
Abba Kaka Yakoura[3,4]
Amadou Zakari[3]

Trial Registration:

NCT04224987 (AVENIR Mortality and Resistance Trial)
NCT04774991 (AVENIR Delivery Trials)
NCT05288023 (AVENIR Programmatic Trial)

Funding:

Bill & Melinda Gates Foundation Planning Grant (OPP1210548);
Bill & Melinda Gates Foundation (INV-002454)

*Principal Investigator
[1]Francis I. Proctor Foundation, San Francisco, California
[2]Centre de recherche et interventions en santé publique (CRISP)
[3]Ministère de la Santé Publique du Niger
[4]Programme National de Santé Oculaire (PNSO)

List of Acronyms

AE	Adverse Event
AMR	Antimicrobial Resistance
BUA	Biological Use Authorization
CDC	Centers for Disease Control and Prevention
CERMES	Centre de recherche médicale et sanitaire
CHW	Community health worker
CNERS	Comité national ethique pour la recherche en santé
CSI	Centre de santé integré, (local health centers)
DALY	Disability-Adjusted Life Year
DCC	Data Coordinating Center
DNA	Deoxyribonucleic Acid
DSC	Division santé communautaire
DSMC	Data and Safety Monitoring Committee
GPS	Global Positioning System
IHME	Institute for Health Metrics and Evaluation
IRB	Institutional Review Board
MOH	Ministry of Health
MUAC	Mid-upper arm circumference
PNSO	Programme national de santé oculaire
RDT	Rapid diagnostic test (for malaria)
SAE	Serious Adverse Event
SAP	Statistical Monitoring Plan
SMC	Seasonal Malaria Chemoprevention
TCC	Trial Coordinating Center
UCSF	University of California, San Francisco
WHO	World Health Organization

Table of Contents

1. Executive Summary 5
1.1. Administrative Information 5
1.2. Trial Details 6
1.3. Revisions History 10
2. Background and Rationale 13
3. Specific Aims 15
4. Methods 16
4.1. Trial Design 16
4.2. Study Setting 16
4.3. Timeline 18
4.4. Mortality/Resistance Trial 20
4.4.1. Trial Design 20
4.4.2. Eligibility 20
4.4.3. Randomization, Adaptation, and Masking 21
4.4.4. Outcomes 23
4.4.5. Sample size 25
4.4.6. Recruitment 26
4.4.7. Adverse Events Monitoring 26
4.4.8. Data Collection, Management, Analysis, and Monitoring 27
4.5. Delivery Trial 29
4.5.1. Trial Design 29
4.5.2. Eligibility 30
4.5.3. Randomization, Adaptation, and Masking 30
4.5.4. Outcomes 32
4.5.5. Sample size 33
4.5.6. Recruitment 33
4.5.7. Adverse Events Monitoring 33
4.5.8. Data Collection, Management, Analysis, and Monitoring 33
4.6. Programmatic Trial 38
4.6.1. Trial Design 38
4.6.2. Eligibility 38
4.6.3. Randomization, Adaptation, and Masking 40
4.6.4. Outcomes 41
4.6.5. Sample size 42
4.6.6. Recruitment 43
4.6.7. Adverse Events Monitoring 43

4.6.8. Data Collection, Management, Analysis, and Monitoring ... 43
5. Ethics and Dissemination ... 47
5.1. Overview ... 47
5.2. Institutional Review Board Approval ... 47
5.3. Informed Consent ... 47
5.4. Potential Risks and Adequacy of Protection Against Risks ... 47
5.5. Compensation for Participants ... 48
5.6. Dissemination Plan ... 48
6. Organizational Structure and Responsibilities ... 49
6.1. University of California, San Francisco Francis I. Proctor Foundation ... 49
6.2. Programme national de santé oculaire (PNSO) ... 49
6.4 Centre de recherche medical et sanitaire (CERMES) ... 49
6.5. Centre de recherche et interventions en santé publique (CRISP) ... 50
6.6. Centres de santé intégré (CSI) ... 50
6.7. Executive Committee ... 50
6.8. Trial Coordinating Center (TCC) ... 50
6.9. Niger Coordinating Center ... 50
6.10. Data Coordinating Center (DCC) ... 51
6.11. Economic Consultants ... 51
6.12. Duties and Responsibilities of Staff ... 52
6.13. Data Safety and Monitoring Committee ... 54
6.14. Policy Matters ... 56
7. Appendices ... 58
7.5. Informed Consent Forms ... 58
7.6. Mortality/Resistance Trial: Training, Procedures, Data Collection Forms ... 58
7.7. Delivery Trial: Training, Procedures, Data Collection Forms ... 58
7.8. Sample Collection: Training, Procedures, Data Collection Forms ... 58
7.9. Laboratory Procedures ... 58
7.10. Data Monitoring Plan ... 58
7.11. Adverse Events Monitoring Plan ... 58
7.12. Integrated Management of Childhood Illness flowchart ... 58
References ... 59

1. Executive Summary
1.1. Administrative Information

Funding	The Bill & Melinda Gates Foundation (OPP1210548, INV-002454)<br>Pfizer donated azithromycin and placebo
Data and Safety Monitoring Committee	David Addiss, MD, MPH, Task Force for Global Health (chair)<br>Mourtala Assao, MD, Niger Ministry of Health<br>Julia Bielicki, MD, PhD, St. George's University of London<br>Allen Hightower, MS<br>Brian Jackson, MD, Children's Hospital of Colorado<br>Fiona Russell, MPHTM, FRACP, PhD, University of Melbourne
Medical Monitors	Dr Amina Seyfoule, MD, Centre de santé mère et enfant, Dosso, Niger<br>Jeremy Keenan, MD, MPH, University of California, San Francisco
Principal Investigators	Amza Abdou, MD<br>Programme national de santé oculaire<br>Email: dr.amzaabdou@gmail.com<br><br>Ahmed Mamane Arzika, PhD<br>Centre de recherche et interventions en santé publique<br>Email: mamaneahmed@yahoo.fr<br><br>Kieran O'Brien, PhD, MPH<br>University of California San Francisco<br>Email: Kieran.Obrien@ucsf.edu<br><br>Tom Lietman, MD<br>University of California San Francisco<br>Email: tom.lietman@ucsf.edu
Participating Institutions	Francis I. Proctor Foundation, University of California San Francisco (UCSF)<br>Ministère de la santé publique du Niger<br>Programme national de la santé oculaire (PNSO)<br>Centre de recherche et interventions en santé publique (CRISP)
Trial Site	Dosso, Tahoua, Maradi, Zinder, and Tillabéri regions in Niger
Ethical Approval	UCSF IRB Number: 19-28287<br>Niger Ethics Approval Record: DELIBERATION N°041/2020/CNERS
Study Contacts	Elodie Lebas<br>Deputy Director of International Programs<br>Francis I. Proctor Foundation, University of California San Francisco<br>Email: elodie.lebas@ucsf.edu

1.2. Trial Details

**Background**	The MORDOR trial found that biannual distribution of azithromycin to children 1–59 months old reduced child mortality. Targeting treatment to children 1–11 months old could reduce antimicrobial resistance by limiting antibiotic distributions while treating children at the highest mortality risk. However, this targeted intervention has not yet been tested.
**Objectives**	AVENIR has three main objectives:<br><br>**1) Mortality.** To assess the efficacy of age-based targeting of biannual azithromycin distribution on mortality.<br>**2) Resistance.** To determine the impact of age-based targeting on antimicrobial resistance by comparing the three arms.<br>**3) Implementation.** To demonstrate and evaluate large-scale implementation.
**Trial Design**	**Mortality/Resistance Trial:** Large simple double-masked cluster-randomized trial with response-adaptive allocation.<br><br>**Delivery Trials:** Cluster-randomized trial.<br><br>**Programmatic Trial:** Cluster-randomized trial.
**Trial Setting**	Accessible rural and peri-urban communities in Niger.
**Summary of Methods**	Eligible communities in Niger will be randomly selected to participate in the three trials:<br><br>In the Mortality/Resistance Trial, 3,000 communities will be randomized to one of three azithromycin distribution strategies: 1) **azithro 1–11:** biannual oral azithromycin to children 1–11 months old with biannual oral placebo to children 12–59 months old, 2) **azithro 1–59:** biannual oral azithromycin to children 1–59 months old, or 3) **placebo:** biannual oral placebo to children 1–59 months old. Interventions will be delivered biannually through a door-to-door census. Mortality will also be monitored through biannual census data collection, which will be used to adaptively allocate treatment assignments after the first year. Communities will retain an allocation for 4 distributions before being re-randomized. Antimicrobial resistance will be monitored using cluster sampling of treated and untreated children and adults in the Dosso region.<br><br>In Delivery Trial I, 80 communities will be randomized in a 1:1 allocation to **door-to-door** or **fixed-point** distribution of azithromycin by community health workers. Implementation outcomes, including

coverage, costs, feasibility, and acceptability, will be compared across delivery strategies.

In Delivery Trial II, 80 communities will be randomized in a 1:1 allocation to one of two arms: 1) **delivery azithro 1–11:** biannual oral azithromycin administration to children aged 1–11 months distributed by community health workers or 2) **delivery azithro 1–59:** biannual oral azithromycin administration to children aged 1–59 months distributed by community health workers. Costs and coverage will be compared across age-based strategies.

In the Programmatic Trial, CSIs will be randomized in a 2:1 allocation to one of two arms favoring the programmatic azithro 1–11 arm: 1) **programmatic azithro 1–11:** biannual oral azithromycin administration to children aged 1–11 months distributed by community health workers or 2) **no intervention:** no distribution of azithromycin. Approximately 1,500 communities from 150 CSIs will be included. All communities still receive routine health services offered by community health workers working for the Niger Ministry of Health's community health program. Antimicrobial resistance will be monitored using cluster sampling from a subset of 20 eligible CSIs per arm (40 CSIs total). Sampling will take place within communities as well as CSIs. Mortality will be monitored through birth history surveys. All infectious cause and cause-specific clinic visits will be monitored through routine CSI data collection.

Primary Outcomes

Mortality/Resistance Trial

- Mortality rate (deaths per 1,000 person-years at risk) among children 1- 59 months of age at 2.5 years from the first enrollment, comparing the azithro 1–59 and placebo arms
- Mortality rate (deaths per 1,000 person-years at risk) among children 1–11 months of age at 2.5 years from the first enrollment, comparing the azithro 1–11 and placebo arms
- Mortality rate (deaths per 1,000 person-years at risk) among children 12- 59 months of age at 2.5 years from the first enrollment, comparing the azithro 1–11 and azithro 1–59 arms

- Prevalence of genetic determinants of resistance to macrolides from nasopharyngeal swabs in children 1–59 months old after 2 years of distributions

- Load of genetic determinants of resistance to macrolides from rectal swabs in children 1–59 months old after 2 years of distributions

Delivery Trial I

- Treatment coverage as defined by the number of doses recorded by the community health workers and eligible population captured during the post-distribution census

Delivery Trial II

- Cost per dose delivered. Program costs will be captured by routine administrative data collection during the study period and by micro-costing activities. Doses delivered will be recorded by community health workers using paper data collection forms and entered electronically by the supervisor team.

Programmatic Trial

- Number of infectious clinic visits recorded by CSIs for children 1-59 months per month.
- Prevalence of genetic determinants of macrolide resistance in nasopharyngeal swabs in children aged 1–59 months after 2 years of distribution
- Load of genetic determinants of macrolide resistance in rectal swabs among children aged 1–59 months after 2 years of distribution

Sample Size

Stage I of the study includes communities enrolled through the primary mortality outcome assessment at 2.5 years from the first region enrollment. Overall, approximately 4,660 communities will be enrolled in Stage I from the Dosso, Tahoua and Maradi regions, with 3,000 communities included in the Mortality Trial, 160 communities in the Delivery Trial, and the remaining 1,500 communities in the Programmatic Trial.

To monitor resistance in the Mortality Trial, 50 communities per arm (150 total) will be randomly sampled from Mortality Trial communities in the Dosso region. Within each sampled community, 30 children aged 1–59 months, 30 children aged 7–12 years, and 30 caregiver/guardians of eligible children will be randomly selected for specimen collections.

To monitor resistance in the Programmatic Trial, 40 CSIs (20 per arm) will be randomly selected from Programmatic Trial CSIs. Of these 40 CSIs, 3 communities per CSI will be selected for sample collection. Within each selected community, 10 children aged 1–59 months will be selected for specimen collections.

Recruitment

Before implementation begins in a region, a sensitization campaign will inform local health authorities and community leaders of study design and nature of participation in the trial.

In addition to IRB and national, regional, and district approval, community-level approval will be obtained before study activities begin in any community.

In the Mortality/Resistance and Delivery Trials, all households will be recruited for participation in the census. In the Mortality/Resistance Trial, all children 1–59 months of age will be recruited for the intervention. In the Delivery Trials, all children 1–59 months of age or 1–11 months of age will be recruited for the intervention, depending on arm allocation.

In the Programmatic Trial, all eligible children aged 1–11 months of age in communities in the catchment area of selected CSIs will be recruited for the intervention.

Household-level consent will be obtained for the census and birth history activities, and caregiver/guardian consent will be obtained for interventions and sample collection.

Data Management

Most data will be collected electronically using a custom mobile application and uploaded regularly to secure, cloud-based servers. Data management teams in Niger and at UCSF will monitor data collection in real time daily, with progress and quality control reports prepared and distributed to investigators weekly.

Sample Management

During collection, samples will be stored at ambient temperature or on ice at 4°C. At the end of each collection period, samples will be transported to a location near the sampled community with facilities to store samples at -20°C as required or directly to CRISP each day. At the end of the collection period, all samples will be transferred to a central storage location at CRISP in Niamey stored at -20°C as required until processed or shipped to UCSF for processing.

1.3. Revisions History

Date	Version	Edit
13-5-20	2	Fixed adding error in Table 2 to reflect the correct grappe interval primary outcomes total of 12,300, Section 4
19-5-20	2	Added Jeremy Keenan as additional US-based Medical Monitor, Section 1
26-5-20	2	Added Elodie Lebas to list of study personnel in title page
29-5-20	2	Added UCSF IRB study number to title page
29-5-20	2	Updated mortality numbers to reflect most recent IHME data, Section 4.2
24-6-20	2	Removed Josette Vingon-Makong from list of Investigators and Study Personnel in title page
30-Jun-20	2	Updated study design schematic, Section 4 (Figure 1)
30-Jun-20	2	Updated sentence to indicate that AMR communities are included in the primary mortality outcome in the Mortality Trial, Section 4.5
30-Jun-20	2	Add sentence about addition of parallel implementation trial with arm description and rationale sentence, Section 4.1
1-Jul-20	3	Revised CRISP in list of collaborators, Section 1
2-Jul-20	4	Added detail about implementation trial, Sections 1.2, 3, 4.1, 4.4.2, 4.4.8, 4.5, 4.6, 4.10
2-Jul-20	4	Updated formatting of table of contents
15-Jul-20	5	Added detail about implementation trial, Sections 1.2, 3, 4.1, 4.4.2, 4.4.8, 4.5, 4.6, 4.10
31-Jul-20	6	Updated detail about implementation trial, Sections 1.2, 3, 4.1, 4.4.2, 4.4.8, 4.5, 4.6, 4.10 and updated formatting
3-Aug-20	7	Reordered outcomes to reflect hierarchical testing order, updated header and page numbers, added Ramatou Maliki to list of investigators, added CSI and CRISP to list of partnering organizations, Section 6, added interim analysis to study timeline, Section 4.6
10-Aug-20	8	Updated list of appendices to include implementation trial procedures, Section 7
12-Aug-20	9	Updated rollout table to include population numbers for all grappes. Section 4.6
16-Sept-20	10	Updated design to reflect DSMC feedback:<br>- Increased number of communities selected for Mortality Trial to 3,000, Section 4<br>- Delayed start of remainder of communities until 1.5 years after the first enrollment (aligned with interim analysis), Section 4.6<br>- Simplified the comparison of Delivery Trial approaches to a 2-arm trial and removed mortality as an outcome, Section 4.5
05-Oct-20	11	Updated with costing/cost-effectiveness protocol changes, Sections 4.10 and 6.10
09-0ct-20	11	Updated References Section to be consistent in formatting
09-0ct-20	11	Added IRB numbers to the Executive Summary, Section 1.1
09-0ct-20	11	Added the English study title to the title page, under the French title
09-0ct-20	11	Updated timing language so that “period” is referred to as “round” and “phase” is referred to as “stage,” Section 4.6 (Tables 1 and 2),

07-Dec-20	12	Added information on the additional 40 MORDOR 54 communities to AMR baseline, Sections 4.10.3 and 4.7.2
31-Jan-21	13	Outcome timepoints were clarified, Sections 1.2, 3, and 4.5
6-May-21	14	Removed "biannual" from the Delivery Trial arms description, Section 4.4.1
6-May-21	14	Removed footnote from Proctor Implementation Outcomes Framework that describes using census data and satellite imagery as a data source for cost, Table 3, Section 4.10.4
6-May-21	14	Changed "nutritional status" to "anthropometric indicators," Section 4.5.2
6-May-21	14	Changed "serological outcomes" to "serological markers of pathogen infection," Section 4.5.3
6-May-21	14	Clarified that M54 grappes will remain untreated until after sample collection has concluded, Sections 4.7.2 and 4.10.3
6-May-21	14	Updated intervention community-level eligibility inclusion criteria to specify that neighboring grappes must be distinguishable from one another, Section 4.3
6-May-21	14	Updated population-based sample collection community-level eligibility inclusion criteria to specify that neighboring grappes must be distinguishable from one another, Section 4.3
6-May-21	14	Updated population-based sample collection community-level eligibility exclusion criteria to specify that grappes treated in a given round prior to sample collection will be excluded from sample collection, Section 4.3
6-May-21	14	Clarified the definition of treatment coverage in the Delivery Trial primary outcomes section as the number of doses recorded by community health workers and census workers, Section 4.5.1
6-May-21	14	Clarified the definition of treatment coverage in the Delivery Trial secondary outcomes section as census projections and satellite imagery, Section 4.5.2
25-October-21	15	Updated footnote in table 2 to clarify distance-based exclusions for the Tahoua region: estimates exclude communities known to be within 5km of district headquarters or town, Section 4.6
31-January-22	16	Updated study design to incorporate details of the Programmatic Trial and the age-based comparison Delivery Trial (referred to as "Delivery Trial II" throughout this document):<br>- Addition of Programmatic Trial specific aims 3a-3c, Section 3<br>- Incorporated the Programmatic Trial and the age-based comparison Delivery Trial design description, Section 4.1<br>- Modified Figure 1, "Stage I design summary" to include Programmatic Trial and Delivery Trial II data collection, Section 4<br>- Updated individual-level eligibility criteria to specify no weight limits in the Programmatic Trial and Delivery Trials, Section 4.3<br>- Incorporated details on randomization, adaptation, masking, and dosing as they relate to the Programmatic and Delivery Trials, Section 4.4<br>- Addition of primary and secondary outcomes for the Programmatic Trial, Section 4.5

		- Included information on sample size in the and sample collections in the Programmatic Trial, Section 4.7<br>- Added detail about data collection and measurement of outcomes in the Programmatic and Delivery Trials, Section 4.10
2-April-22	17	- Inclusion of CSI-based collections in the Programmatic Trial, Sections 1.2, 4.1, 4.3, 4.7.2, and 4.10.3<br>- Addition of microbiome outcomes, Section 4.5.2<br>- Inclusion of additional 60 baseline AMR collections in Round 4, Sections 4.7.2, and 4.10.3
29-June-22	18	- Removal of grappe level eligibility restrictions in the Programmatic Trial eligibility, Section 4.6.2
17-November-22	19	- Added detail on random selection of participants for AMR collections
30-November -22	20	- Updated personnel
3-February-23	21	- Increased number of communities in programmatic AMR collections
9-May-2023	22	- Added a footnote to Figure 2: Stage I design Summary, that specifies that a random sample of 150 communities was selected for AMR monitoring, 60 of which contribute to the primary outcome
10-May-2023	23	- Updated Figure 1: Stage I design Summary and section 4.4.5.1 Overview to show that 3,000 grappes have been randomized
19-May-23	24	- Updated section 4.4.8.2 to add a description of the random census verification and verbal autopsy to measure mortality outcomes
13-Oct-23	25	- Reviewed for clarity and completeness and updated enrollment numbers
27-Oct-23	26	- Primary outcome for programmatic trial changed to all infectious clinic visits
21-Nov-23	27	- Updated Delivery II primary and secondary outcomes to match statistical analysis plan (Section 4.5.4)
27-Nov-23	28	- Updated Mortality secondary outcome to include CSI data extraction to assess morbidity (Section 4.4.4.2)
2-Feb-24	29	- Added paragraph to include the additional 60 communities (Section 4.4.5.2)<br>- Added sentence stating all same procedures will be followed for the additional 60 communities (Section 4.4.8.3)
26-Sept-24	30	- Update AMR analysis to include 150 communities
22-Dec-25	31	- Added data source for Figure 1

2. Background and Rationale

Sub-Saharan African countries have drastically reduced under-5 mortality over the past 20 years, yet in many areas, 10% of children still do not survive to the age of 5.[1,2] Although some areas are well poised to achieve the Sustainable Development Goal (SDG) target to end preventable under-5 mortality by 2030, many settings in western and central Africa must achieve unprecedented rates of decline to meet the SDG goals.[2,3] Under-5 mortality in Niger is among the highest globally, with estimates at 108 deaths per 1,000 livebirths,[1] more than 4 times the SDG target of 25 deaths per 1,000 livebirths.[3] In high mortality settings like Niger, highly effective and feasible interventions are urgently needed.

MORDOR I demonstrated that community-level biannual distribution of oral azithromycin to children 1–59 months of age reduced mortality by nearly 14% compared to placebo over 2 years in Malawi, Niger, and Tanzania.[4] The greatest effect was found in Niger, where communities receiving azithromycin experienced an 18% reduction in mortality compared to those receiving placebo.[4] Subgroup analyses suggested that the youngest children might benefit the most, with reductions of approximately 23% in children 1–11 months old.[4] In Niger, azithromycin distributions were associated with an increase in antimicrobial resistance (AMR) to macrolides after 2 years,[5] though azithromycin remained similarly effective in reducing mortality after 3 years in MORDOR II.[6]

The World Health Organization (WHO) released conditional guidelines for this intervention which include targeting azithromycin distributions to children 1–11 months of age in high mortality settings.[7] The rationale for targeting this age group is that the youngest children experience the highest risk of mortality and limiting distributions to the youngest children could minimize selection for antimicrobial resistance. However, distributing azithromycin to children 1–11 months alone remains untested, since MORDOR I treated children 1–59 months of age. A crude examination of relative mortality reductions by age group suggests a stronger effect in the younger age groups, but MORDOR was unable to demonstrate a differential effect by age group statistically ($P = 0.20$ for test of interaction of treatment and age).[4] Moreover, a herd effect could have contributed to the apparent larger effect sizes in the younger groups; treating children 12–59 months may protect children 1–11 months by reducing their exposure to infectious diseases causing mortality. Finally, beyond a possible differential effect by age group, in MORDOR I-Niger there were 5 times as many children aged 12–59 months as children 1–11 months, resulting in nearly twice as many deaths averted in the older age group.[8] The greatest societal gains may thus come from intervening on a population with a broader risk spectrum instead of targeting only the highest risk subgroups.[9] More evidence is needed to determine the difference and magnitude of the benefit of targeting different age groups, and of the risk of inducing AMR when distributing to different age groups.

The AVENIR trial was designed to test the effect of age-based targeting on mortality and resistance. Several design features allow for these assessments. First, as mortality is a rare event even in high mortality settings, a large sample size is required to detect modest intervention effects[10]. AVENIR thus uses a large simple trial design, with a simple intervention, simple data collection, and simple outcome assessment allowing for implementation in a very large population. Second, AVENIR is cluster randomized because of the community-based nature of the intervention as well as to address potential contamination within communities[11]. As the mechanism of azithromycin's effect is likely through its impact on infectious disease, community-level randomization prevents an individual's treatment assignment from affecting the outcomes of individuals with different assignments within a community. Finally, AVENIR will use response-adaptive allocation to achieve several aims. This approach allows for more ethical

allocation, as communities will have a higher probability of receiving the intervention that reduces mortality the most. By maintaining an allocation of at least 10% of communities to placebo, continued monitoring of efficacy is still possible. This includes the detection of waning effects over time which might indicate the appropriate duration of the intervention. The adaptive design also allows for greater flexibility, enabling the existing trial infrastructure to be used to evaluate other interventions. The study can adapt to changing guidelines by randomizing in new intervention arms, for example, rather than being constrained by the original design which could become irrelevant during the course of the trial. For example, unanticipated WHO recommendations could include changes to the target age range or distribution frequency. Finally, as countries consider implementation of this intervention for child survival, AVENIR provides an opportunity to demonstrate and rigorously assess large-scale implementation of this program.

3. Specific Aims

The overall objectives of trial are to determine the optimal age group to treat with biannual oral azithromycin distribution to reduce child mortality and to compare selection for antimicrobial resistance across different age-based strategies for azithromycin distribution. In a series of secondary outcomes and parallel studies, the trial will also evaluate large-scale implementation. We propose the following specific aims for the Mortality/Resistance trial:

Specific Aim 1.

1a. To compare efficacy of biannual oral azithromycin distribution to reduce mortality in children 1–59 months old in communities distributing azithromycin to ages 1–59 months (azithro 1–59) compared to placebo. *We hypothesize that the community-level mortality rate in children 1–59 months old at 2.5 years from the first enrollment will be lower in the azithromycin arm.*

1b. To compare efficacy of biannual oral azithromycin distribution to reduce mortality in children 1–11 months old in communities distributing azithromycin to ages 1–11 months (azithro 1–11) compared to placebo. *We hypothesize that the community-level mortality rate in children 1–11 months old at 2.5 years from the first enrollment will be lower in the azithromycin arm.*

1c. To compare efficacy of biannual oral azithromycin distribution to reduce mortality in children 12–59 months old in communities distributing azithromycin to ages 1–11 months (azithro 1–11) compared to 1–59 months (azithro 1–59). *We hypothesize that the community-level mortality rate in children 12–59 months old at 2.5 years from the first enrollment will be lower in the azithro 1–59 arm.*

Specific Aim 2.

2a. To compare the community-level prevalence of genetic determinants of macrolide resistance in nasopharyngeal samples from children 1–59 months old in programs targeting azithromycin to 1–11 months, 1–59 months, or providing placebo. *We hypothesize that the community-level prevalence of resistance determinants after 2 years of distributions will differ in these 3 arms. Specifically, we hypothesize that the community-level prevalence of resistance determinants will be greater in the azithromycin arms than the placebo arm. In addition, we hypothesize that the prevalence of resistance will be greater in the azithro 1–59 arm than the azithro 1–11 arm.*

2b. To compare the community-level load of genetic determinants of macrolide resistance in rectal samples from children 1–59 months old in programs targeting azithromycin to 1–11 months, 1–59 months, or providing placebo. *We hypothesize that the community-level load of resistance determinants after 2 years of distributions will differ in these 3 arms. Specifically, we hypothesize that the community-level load of resistance determinants will be greater in the azithromycin arms than the placebo arm. In addition, we hypothesize that the load of resistance determinants will be greater in the azithro 1–59 arm than the azithro 1–11 arm.*

4. Methods

4.1. Trial Design

AVENIR uses a large simple cluster-randomized placebo-controlled adaptive platform design to conduct multiple studies. Eligible communities in Niger will be randomly selected for participation in the Mortality/Resistance Trial, the Delivery Trials, or the Programmatic Trial. Figure 1, Figure 2, Table 1, and Table 2 summarize the design, eligible population, and anticipated rollout of the platform.

4.2. Study Setting

The study will take place in accessible rural and peri-urban communities in the 5 regions of Niger with the largest under-5 populations. This includes communities in the Dosso, Tahoua, Maradi, Zinder, and Tillabéri regions (Figure 2). According to Institute for Health Metrics and Evaluation (IHME) projections to 2020[12], the average regional under-5 mortality rate (number of deaths per 1,000 livebirths) is 116 in Dosso, 101 in Tahoua, 110 in Maradi, 120 in Zinder, and 116 in Tillabéri. Agadez, Diffa, and the capital area of Niamey have much smaller under-5 populations than the other regions and some areas are currently inaccessible to study teams due to security concerns and thus are not included in the trial (see Section 4.3 Eligibility).

Figure 1. Eligible regions in Niger with estimated total under-5 population and study timeline.

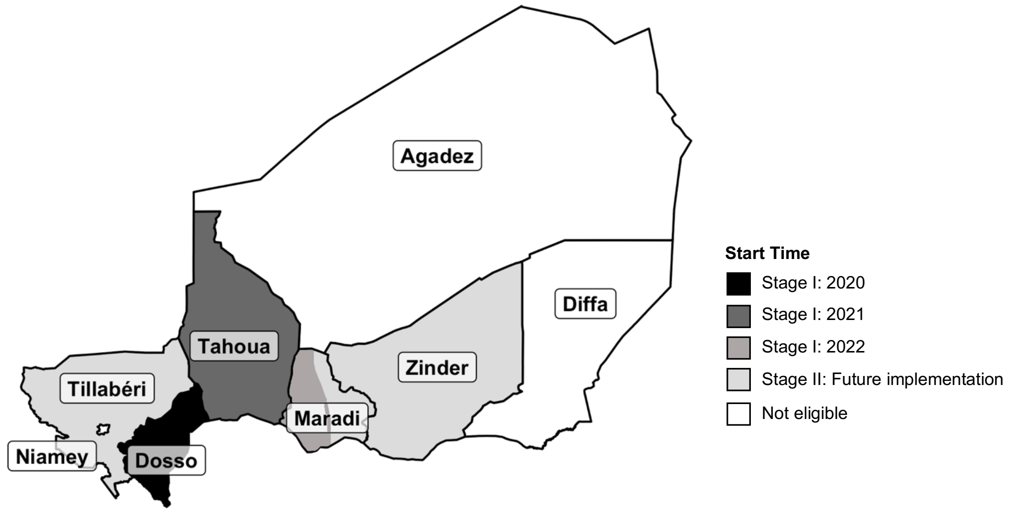

United Nations Office for the Coordination of Humanitarian Affairs (OCHA) Centre for Humanitarian Data. *Niger - Subnational Administrative Boundaries (COD-AB-NER)*. HDX (Humanitarian Data Exchange). https://data.humdata.org/dataset/cod-ab-ner.

Figure 2. Stage I design summary

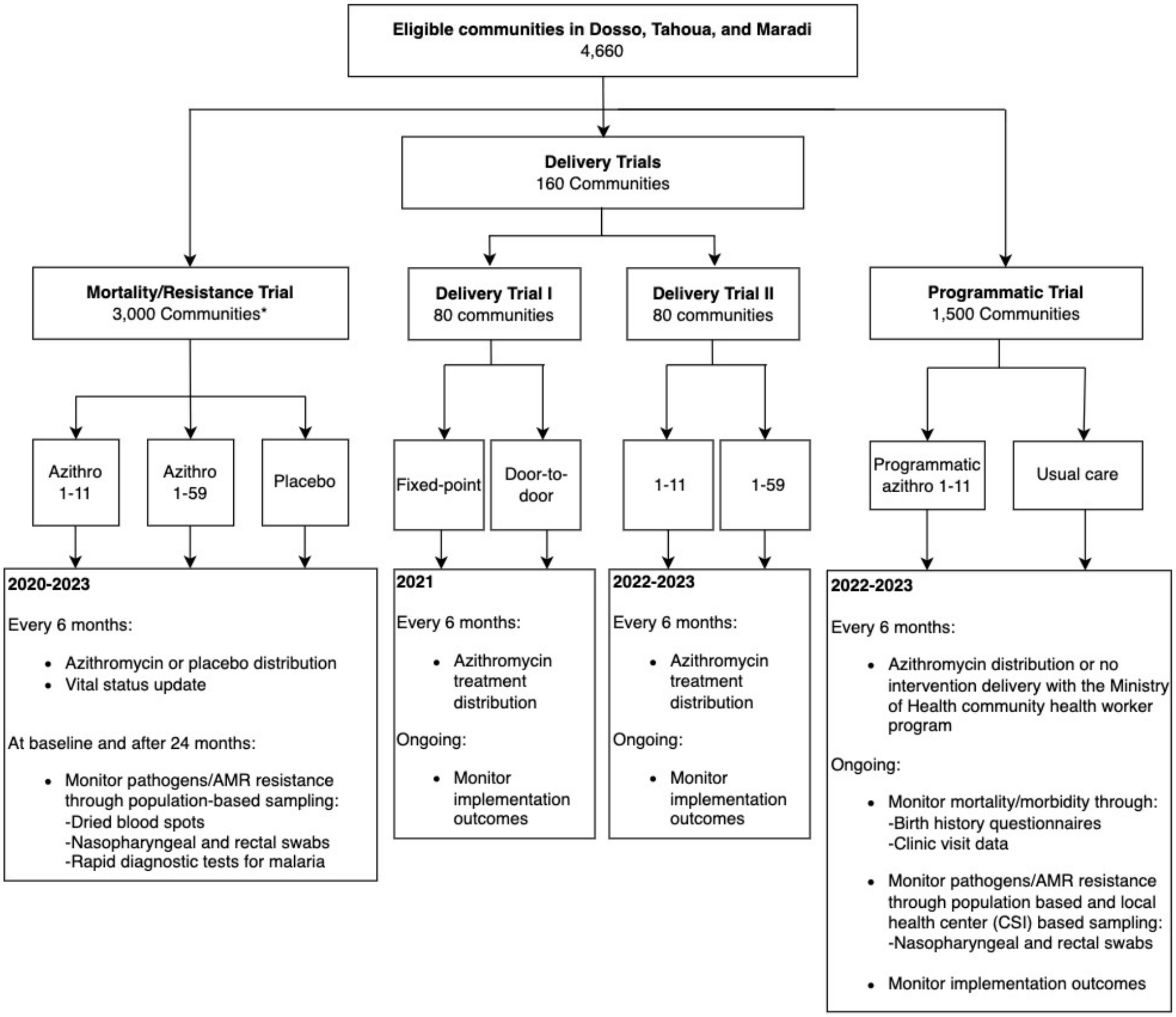

*A random sample of 150 communities was selected for AMR monitoring, 150 of which contribute to the primary

2

4.3. Timeline

The study will take place over a 4-year period beginning Fall 2020 (Table 1). Implementation will be phased in by region. Study progress is defined in “census rounds” which refer to a single 6-month census data collection and treatment period, and “inter-census intervals”, which refer to the interval between two census rounds, required to assess the primary outcome of mortality in the Mortality Trial. Dosso will be enrolled in the first year and Tahoua in the second year. Parts of Tahoua and Maradi will be added to the Programmatic Trial in the second and third years.

Table 1. Estimated eligible study population and communities with intervention roll out in Stage I and II.

			Stage I[1]					Stage II[2]		
			Year 1		Year 2		Year 3	Year 3	Year 4	
**Region**	**Estimated eligible under-5 population[3]**	**Estimated eligible communities**	**Round 1**	**Round 2[4]**	**Round 3[5]**	**Round 4**	**Round 5**	**Round 6**	**Round 7**	**Round 8**
Dosso	271,273	1,800	X*	X	X	X*	X*	X	X	X
Tahoua (1)	245,944	1,225			X	X*	X	X	X	X
Tahoua (2)	245,944	1,225				X	X	X	X	X
Maradi (1)	342,889	1,000					X	X	X	X
Maradi (2)	342,890	2,000						X	X	X
Zinder	452,241	3,860							X	X
Tillabéri	440,791	3,070								X
**Total:**	**2,341,972**	**14,180**								

X = census and/or treatment; X* = census and/or treatment + resistance collections in a sample of communities
[1]Stage I through primary outcome analysis at 2.5 years from the first enrollment.
[2]Stage II dependent upon primary outcome, resources, and security restrictions.
[3]As estimated from projections by the Institute for Health Metrics and Evaluation multiplied by fraction of eligible grappes in each region.
[4]Delivery trial will begin at the beginning of round 2 in year 1
[5]Interim analysis at approximately 1.5 years (end of round 3). At this time, intervention will begin in Programmatic Trial Communities.

Table 2. Estimated eligible population by trial and region for Stage I.

**Region**	**Estimated eligible under-5 population[1]**	**Estimated eligible communities[2]**	**Mortality/Resistance trial communities[3,4]**	**Delivery trial communities**	**Programmatic communities**
Dosso	271,273	1,744	1,584	160	0
Tahoua	491,888	1,916	1,416	0	500
Maradi (1st half)	342,889	1,000	0	0	1,000
**Total**	**1,106,050**	**4,660**	**3,000**	**160**	**1,500**

[1]As estimated from projections by the Institute for Health Metrics and Evaluation multiplied by fraction of eligible grappes in each region.
[2]Communities with total populations between 250 and 2,499 not designated as urban quartiers according to the 2012 Niger national census. Dosso and Tahoua region estimates further exclude communities known to be located within 5 km of district headquarters town. Distance-based exclusions pending for Maradi.
[3]150 AMR communities will be selected from among the Dosso Mortality/Resistance Trial communities to undergo collections for resistance monitoring

4.4. Mortality/Resistance Trial

4.4.1. Trial Design

In the Mortality Trial, communities in the Dosso region in Niger will be randomized in a 1:1:1 fashion to: **1) azithro 1–11:** biannual azithromycin to children 1–11 months old with placebo to children 12–59 months old, **2) azithro 1–59:** biannual azithromycin to children 1–59 months old, or **3) placebo:** biannual placebo to children 1–59 months old. For regions enrolled after the one-year run-in period, the randomization allocation will be updated based on the probability of mortality in children 1–59 months in each arm. Communities will retain their allocation for 4 distributions. A dedicated study team will conduct a biannual door-to-door census to deliver the intervention and monitor mortality. Within the Mortality Trial, 150 communities in the Dosso region will be randomly selected from among the eligible communities, similarly randomized 1:1:1, and followed to monitor antimicrobial resistance (Figure 1).

The initial design compares the efficacy of different age-based azithromycin distribution strategies to reduce child mortality. After the primary outcome analysis additional treatment arms may be added to the trial or arms may be dropped from the trial. With such changes, protocol details, power calculations, and analysis plans will be updated accordingly in the protocol and statistical analysis plan.

4.4.2. Eligibility

<u>**Intervention**</u>

At the community-level, eligibility includes:

<u>Inclusion criteria:</u>

- Location in Dosso, Tahoua, Maradi, Zinder, or Tillabéri regions
- Population 250 to 2,499*
- Distance > 5 km from district headquarters town
- Distinguishable from neighboring communities
- Verbal consent of community leader(s)

<u>Exclusion criteria:</u>

- Inaccessible or unsafe for study team
- “Quartier” designation on national census

*Population size as estimated from the most recent national census or projections

At the individual-level, eligibility includes:

<u>Inclusion criteria:</u>

- Age 1–59 months
- Primary residence in a study community
- Verbal consent of caregiver/guardian for study participation
- Weight ≥ 3.0 kg

<u>Exclusion criteria:</u>

- Known allergy to macrolides

<u>**Population-based sample collections**</u>

At the community-level, eligibility includes:

<u>Inclusion criteria:</u>

- Location in study region
- Distinguishable from neighboring communities
- Verbal consent of community leader(s)

<u>Exclusion criteria:</u>

- Inaccessible or unsafe for study team
- Included in MORDOR trials
- Not randomly selected
- Received treatment prior to sample collection

At the individual-level, eligibility includes:

<u>Inclusion criteria:</u>

- Age 1–59 months or 7-12 years* or caregiver/guardian of a child eligible for treatment
- Primary residence in a study community selected for sample collections
- Written consent of caregiver/guardian for study participation

**7-12 years at baseline or under 12 years and not previously eligible for treatment at follow-up*

<u>Exclusion criteria:</u>

- Not on list of randomly selected participants from the census

4.4.3. Randomization, Adaptation, and Masking

4.4.3.1. Randomization

The randomization unit for the Mortality/Resistance Trial will be the grappe, which is the smallest health care unit administratively defined in Niger, hereafter referred to as “community.”

Communities will be randomized to the following 3 arms:

- **Azithro 1–11:** biannual weight- or height-based dose of oral azithromycin suspension to children 1–11 months old and oral placebo to children 12–59 months old
- **Azithro 1–59:** biannual weight- or height-based dose of oral azithromycin suspension to children 1–59 months old
- **Placebo**: biannual weight- or height-based dose of oral placebo to children 1–59 months old

4.4.3.2. Adaptation

The randomization sequence will be generated by the trial biostatistician at UCSF.

In the Mortality/Resistance Trial, Dosso communities will undergo the first randomization in a 1:1:1 allocation to the 3 arms and will retain their allocation throughout the first stage of the study. For communities in other regions enrolled after Dosso, the allocation will adapt based on prior mortality outcomes from the Mortality Trial and each cluster’s assignment will be retained

through the primary outcome assessment. After the primary outcome assessment, allocation probabilities will be updated after every 4 distributions.

After the Mortality Trial's primary outcome analysis, the study team and key stakeholders will determine whether the Programmatic Trial continues. If the study continues, the Mortality Trial communities will be included in the Programmatic Trial and additional regions will be added over time (Figure 2). In this platform design, additional treatment arms may be added to the trial or arms may be dropped from the trial based on additional information from other studies, updates to the WHO guidelines, or preferences of programs or policymakers in Niger. With such changes, protocol details, power calculations, and analysis plans will be updated accordingly in the protocol and statistical analysis plan.

Refer to the trial's Statistical Analysis Plan (SAP) for complete details of the adaptive randomization algorithm.

4.4.3.3. Masking

In the Mortality/Resistance Trial, we will use a matching placebo to mask study arm allocation. Placebo will be identical to azithromycin in appearance, smell, and packaging. Treatment assignment will be masked by assigning a series of upper- and lower-case letters to each trial, age group, and treatment arm. Those masked to study arm allocation include participants, investigators, most study personnel including census personnel administering treatment and collecting mortality outcome data and the data analysis team, and laboratory personnel processing samples for resistance outcomes. Unmasked personnel include the trial biostatistician and data analyst responsible for implementing the randomization sequence and key members of Pfizer staff. Key members of the Pfizer staff must be unmasked to implement the study drug treatment allocation, and to review SAE reporting. Masking will minimize bias introduced through knowledge of treatment assignment and will ensure that the census and outcome assessments are identical in all study communities.

4.4.3.4. Interventions

Description of Study Medicine

Azithromycin (Zithromax®) for oral suspension is supplied in bottles containing azithromycin dehydrate powder equivalent to 1200 mg per bottle and the following inactive ingredients: sucrose; tribasic anhydrous sodium phosphate; hydroxypropyl cellulose; xanthan gum; FD&C Red #40; and flavoring including spray dried artificial cherry, crème de vanilla, and banana. After constitution, a 5 ml suspension contains 200 mg of azithromycin.

Placebo is identical in appearance, with identical labeling, and contains no active ingredient.

4.4.3.5. Dosage Information

Azithromycin or placebo will be administered as a single dose, in oral suspension form for children, of 20 mg/kg in children (up to the maximum adult dose of 1 g). In the Mortality/Resistance Trial, weight-based dosing will be used via hanging scale for children 1–11 months of age. For children 12–59 months of age, height-based dosing will be used via height-stick approximation as currently performed by Niger's trachoma program.

Both dosing cups and syringes will be used to administer treatment. For children too young to

drink out of a dosing cup, a 1 ml or 5 ml syringe will be used, and the calculated dose will be rounded upwards to the nearest 0.2 ml.

4.4.3.6. Side Effects

Azithromycin is usually well tolerated. The most common side effects are diarrhea, nausea, abdominal pain, and vomiting. These side effects occur in fewer than 5% of persons who receive azithromycin and have been rarely reported in studies of the mass distribution of azithromycin for trachoma or child mortality. Adverse events will be monitored as described in Section 4.10.

There is a possibility of an increased risk of infantile hypertrophic pyloric stenosis in neonates, who are excluded from this trial.

Pfizer, Inc. will provide the donation of azithromycin and placebo, which will be shipped directly to Niger and received by a representative of the Nigerien Ministry of Health, who will manage the customs process and transport the medication from the port to a storage site. The exemption of duties and taxes will be settled by the Nigerien Customs Authorities and the Nigerien Ministry of Health.

4.4.3.7. Study Medication Storage and Accountability

Azithromycin and placebo will be stored between 15° to 30°C (59° to 86°F), as recommended by Pfizer. A record of the exact quantity of oral suspension dispensed will be kept by the treatment distribution teams.

4.4.3.8. Medication Quality Control

Study medication will be stored in project offices prior to use. The study team will regularly check and record the temperature of the drug storage rooms and the study medication expiry dates. The expiration dates on the medication containers will be strictly monitored and all expired study medicine will be discarded appropriately.

4.4.4. Outcomes

4.4.4.1. Primary Outcomes

- Mortality rate (deaths per 1,000 person-years at risk) among children 1- 59 months of age at 2.5 years from the first enrollment with a window of up to 3 months, comparing the azithro 1–59 and placebo arms
- Mortality rate (deaths per 1,000 person-years at risk) among children 1- 11 months of age at 2.5 years from the first enrollment with a window of up to 3 months, comparing the azithro 1–11 and placebo arms
- Mortality rate (deaths per 1,000 person-years at risk) among children 12- 59 months of age at 2.5 years from the first enrollment with a window of up to 3 months, comparing the azithro 1–11 and azithro 1–59 arms

- Prevalence of genetic determinants of resistance to macrolides from nasopharyngeal swabs in children 1–59 months old after 2 years of distributions
- Load of genetic determinants of resistance to macrolides from rectal swabs in

children 1–59 months old after 2 years of distributions

4.4.4.2. Secondary Outcomes

- Mortality rate (deaths per 1,000 person-years at risk) among children ages 12–59 months over 2.5 years from the first enrollment with a window of up to 3 months, comparing the azithro 1–11 and placebo arms
- Mortality rate (deaths per 1,000 person-years at risk) among children ages 1–11 months over 2.5 years from the first enrollment with a window of up to 3 months, comparing the azithro 1–11 and azithro 1–59 arms
- Mortality rate over 2.5 years from the first enrollment with a window of up to 3 months by subgroups:
    - Region (Dosso, Tahoua, Maradi, Zinder, and Tillabéri)
    - Number of previous community distributions
    - Timing of distribution/seasonality
    - Other interventions
        - Participation in MORDOR trials
        - Participation in and coverage of Seasonal Malaria Chemoprevention (SMC)
        - Participation in trachoma distributions of azithromycin
    - Anthropometric indicators as defined by weight, MUAC, height interval
    - Distance to nearest healthcare facility

- Prevalence of genetic determinants of resistance to macrolides from nasopharyngeal swabs and load of genetic determinants of resistance to macrolides from rectal swabs after two years of distributions in:
    - Children 7-12 years old
    - Caregivers/guardians of eligible children
- Prevalence of genetic determinants of resistance to other antibiotic classes from nasopharyngeal swabs and rectal swabs in all samples after 2 years of distributions
- Proportion of macrolide resistant pneumococcal isolates from nasopharyngeal swabs in children 1–59 months after 2 years of distributions
- Prevalence of malaria as assessed by RDT during sample collections at baseline and after two years of distributions

- Program costs as captured by routine administrative data collection during the study period and by micro-costing activities
- Cost-effectiveness measured by estimated program costs and using measured mortality and estimated DALYs as the effectiveness outcome, compared across arms.

- Change in anthropometric indicators over time as defined by weight, mid-upper arm circumference (MUAC), and height interval
- All infectious clinic visits among children 1–59 months of age in the study area during the study period as assessed through passive surveillance of CSI records
- Cause-specific clinic visits among children 1–59 months of age in the study area during the study period as assessed through passive surveillance of CSI records, including visits with diagnoses of respiratory infection, diarrheal disease, and malaria

Microbiome outcomes for rectal swab collections

- Gamma diversity, estimated as the alpha diversity of the pooled community sample using the Shannon diversity index
- Overall microbial structure using Manhattan distance
- Relative abundance of individual microbial taxa at genus and species level

4.4.4.3. Other Outcomes

- Cause-specific mortality by treatment arm as defined by verbal autopsy* over 2.5 years from the first enrollment
- Serological markers of pathogen infection as assessed by dried blood spots after 2 years of distributions
- Normalized read number of genetic determinants of resistance by antibiotic class in environmental samples after 2 years of distributions

*Depending upon resources, verbal autopsy might be conducted by UCSF or another group

4.4.5. Sample size

4.4.5.1. Overview

We estimate that there are approximately 14,180 eligible communities encompassing approximately 2.3 million children under 5 across the 5 eligible regions (Table 1). For the first stage of the study, we estimate we will include 4,660 eligible communities in Dosso, Tahoua, and part of Maradi, with a total of 1.1 million eligible children under 5 (Table 2).

Stage I includes data collected through the primary outcomes. In Stage I, 3,000 communities will be selected for the Mortality/Resistance Trial, 160 for the delivery trials, and 1,500 will receive for the Programmatic Trial. The remaining eligible communities in Maradi, Zinder and Tillabéri will be included in the study during Stage II. Table 2 details the Stage I study population by region.

Refer to SAP for details of power calculations based on this sample size.

4.4.5.2. Sample Collections

150 of the communities in the Mortality Trial in the Dosso region will be randomly selected to be followed to monitor antimicrobial resistance. In each community, 30 children aged 1–59 months, 30 children 7-12 years, and 30 caregivers/guardians of eligible children will be randomly selected for sample collections at baseline and after 4 distributions. Samples will be collected in a random subset of 60 communities at baseline, and in all 150 AMR communities after 4 rounds (approximately 2 years).

An additional 40 communities that participated in the MORDOR 54 trial will be randomly selected for inclusion in sample collections at baseline. Of these 40 communities, 20 will be randomly selected from among those who received 5 rounds of azithromycin during MORDOR, and the other 20 communities be randomly selected from among those who received 3 rounds of azithromycin and 2 rounds of placebo during MORDOR. In each community, 30 children 1–59 months will be randomly selected to receive a rectal swab.

Furthermore, 60 communities whose children aged 1-59 months have received ~11 biannual

distributions of azithromycin (as opposed to placebo) during the MORDOR trial will be selected randomly: 20 randomized in AVENIR to two more years of biannual azithromycin to 1-59 month olds, 20 randomized to biannual azithromycin to 1-11 month olds, and 20 randomized to biannual placebo. 40 children per grappe, aged 1-59 months, will be randomly selected from the prior census. Examiners will perform a nasopharyngeal swab on each child, stored in STGG media.

In all communities selected for sample collections, treatment distribution for that round will be conducted after sample collections.

Refer to SAP for details of power calculations.

4.4.6. Recruitment

Before implementation begins in a region, a sensitization campaign will inform local health authorities and community leaders of the study design and nature of participation in the trial. In addition to IRB and national-, regional-, and district-level approval, community-level approval will be obtained before study activities begin in any community. All households will be recruited for participation in the census, and all children 1–59 months of age will be recruited for the intervention. Household-level consent will be obtained for the census activities, and caregiver/guardian consent will be obtained for interventions and sample collection. Details of informed consent are found in Section 5.3.

4.4.7. Adverse Events Monitoring

During the consent process, the common adverse reactions that may occur will be explained to caregivers/guardians. At the time of treatment, caregivers will be instructed to report to the study team any adverse event experienced by the treated child within 28 days of treatment. Children will be referred for follow up care on a case-by-case basis.

4.4.7.1. Adverse Events Reporting

Serious adverse events (SAE) will be reported to Pfizer. An IIR SAE Form (Investigator-Initiated Research Serious Adverse Events Form) will be completed for each event.

According to Pfizer, an SAE is any adverse event that:
Results in death
Is life-threatening (i.e., causes an immediate risk of death)
Requires inpatient hospitalization or prolongation of existing hospitalization
Results in persistent or significant disability or incapacity
Results in a congenital anomaly or birth defect or that is considered to be:
An important medical event

All caregivers will be advised to alert a community health worker or study team member if their child experiences an adverse event within 28 days of distribution. The study team will be trained to identify serious adverse events, which must be submitted to UCSF within 24 hours. UCSF will review and submit SAEs to Pfizer and the Medical Monitor, and to the DSMC as required.

Deaths that are reported to the study team outside of the biannual census (primary Mortality Trial outcome) will be reported as an SAE to Pfizer. Note that deaths identified via the biannual

census, which constitute the primary outcome, will not be reported as SAEs. Deaths that are reported to the study team as part of the biannual census will be reported to Pfizer in aggregate, not by arm, quarterly.

See the Adverse Events Reporting procedures for further details

4.4.8. Data Collection, Management, Analysis, and Monitoring

4.4.8.1. Data Collection Overview

All Mortality/Resistance Trial data will be collected electronically on mobile devices with a custom-designed mobile application using the CommCare mobile data collection platform by Dimagi.

4.4.8.2. Measurement of Mortality Outcomes

Mortality will be monitored through biannual census data collection in the Mortality/Resistance Trial. Trained census workers will use a mobile application on smartphones to collect data. Census workers will visit each household in assigned study communities to obtain household consent for participation, and to collect demographic data on the head of household, all mothers in the household, and all children 1–59 months in the household. For eligible children, dose will be determined via weight using a hanging scale for children under 12 months of age or height-stick approximation for children 12 months and older using the Niger trachoma program's height stick. MUAC will also be assessed on all eligible children. The census worker will administer the dose and record the child's treatment status. At subsequent census rounds, the census workers will return to each household and update the vital status of all children, indicating whether a child is alive, has died, has moved, or has an unknown status. New children and households will be added. The census will be updated at 6, 12, 18, 24, 30 and 36 months from baseline. A subset of communities will be randomly selected to receive a repeat census at the same time point to validate data collection.

For Mortality Trial communities randomly sampled for additional monitoring, census data collection will include children 7-12 years of age (see Section 4.10.3). In these communities, treatment will be conducted after samples have been collected.

Verbal and social autopsies may be conducted on deaths that occur during the study and are identified during the census, depending on the availability of resources. Verbal autopsies will be performed on children who were 1-2 months of age at the time of treatment and were recorded as died on the subsequent census.

A subset of Mortality Trial communities will also receive a birth history survey in addition to the regular census.

4.4.8.3. Measurement of Sample Based Outcomes

In the Mortality and Resistance Trial, 150 AMR communities from the Dosso region will be randomly selected from the pool of eligible communities and followed to monitor antimicrobial resistance. Samples will be collected in a random subset of communities at baseline and in all 150 AMR communities after 2 years of distributions. For primary outcomes, samples will be processed from a subset of 150 communities.

In each of the selected Mortality/Resistance Trial communities, 30 children aged 1–59 months,

30 children aged 7–12 years, and 30 caregivers/guardians of eligible children will be randomly selected for additional monitoring. Nasopharyngeal samples will be collected from all sampled individuals. From children aged 1–59 months, rectal swabs, RDT, and dried blood spots will also be collected. In the sample of children aged 1–59 months, 10 children will be randomly selected for an additional nasopharyngeal swab which will be used for phenotypic testing. Samples will be collected at baseline and after 2 years of distributions.

To randomly select individuals for population-based monitoring in the Mortality/Resistance Trial, census data will be used. In the 150 Dosso communities selected for additional monitoring, the baseline and 24-month census will be separate from treatment and will include data collection on children aged 7–12 years old. The random samples of children in both age groups and caregivers/guardians will be selected from the census. To ensure that samples are collected on 30 individuals in each group, 50 children in both age groups and 50 caregivers/guardians will be randomly selected from the census data.

60 previously untreated communities will be incorporated into the Mortality/Resistance Trial in Round 4 and will receive rectal swab baseline collections. This is to account for rectal swab baseline samples collected in Round 1 that were damaged and unusable. 1 rectal swab will be collected from 30 children aged 1–59 months per community. Samples from community latrines and chicken feces will also be collected when available from the homes of the 30 children selected for this sample collection.

Environmental samples will be taken from communities and households from which participants were selected for data collection at baseline and 24 months. Latrine and water samples will be taken at the community level, and animal feces, stored water, and dirt samples will be taken at the household level.

Once the census is complete and the sample is generated, the study team will work with a local mobilizer to invite the sampled group to a central location in the community for sample collection. Samples will be collected by trained personnel and linked to census data through bar codes. Once sample collection is complete, the census team or community health workers will visit the study community to complete treatment for that round.

During collection, samples will be stored at ambient temperature or on ice at 4°C. At the end of each collection period, samples will be transported to a location near the sampled community with facilities to store samples at -20°C as required or directly to CRISP each day. At the end of the collection period, all samples will be transferred to a central storage location at CRISP in Niamey stored at -20°C as required until processed or shipped to UCSF for processing. Refer to Sample Collections Procedures and the Laboratory Procedures for full details.

All the same procedures will be followed for the additional communities that will be added from MORDOR.

4.4.8.4. Measurement of Additional Outcomes

Data on other programs that include the distribution of antibiotics in the study area will be collected. Seasonal Malaria Chemoprevention (SMC) implementation and coverage data will be collected from SMC implementers at the district and community level during the study. Trachoma distributions will be tracked, and prior distributions recorded for each community through communications with PNSO.

4.4.8.5. Data Collection and Management

All study personnel who assess outcomes will attend intensive, two-day training sessions prior to conducting study procedures. Training will include operation of the mobile device, data collection through the mobile application or on paper, and the study protocol and procedures.

Where possible, data will be collected electronically using a custom-designed mobile census application (CommCare, Dimagi) on mobile devices and uploaded regularly to a secure, cloud-based server. Where possible, data will be uploaded in real time using the cellular network, or weekly or monthly at a central site with strong Wi-Fi or cellular connection. In the Delivery Trial and Programmatic Trial communities, data on intervention administration will be recorded by community health workers on paper forms, and later entered electronically.

Mobile devices will be accessed via unique usernames and passwords. Mobile devices will be stored in locked offices with electricity for charging.

Validation will be used in development of the data collection forms to ensure to a large extent that there are no inconsistencies or invalid data. Data management teams in Niger and at UCSF will monitor data collection in real time daily. Progress and quality control reports will be prepared and distributed to investigators weekly.

Study participants will be assigned unique identification numbers that do not contain identifying information, which will be used to link data across databases and will be the only identifier shared in de-identified datasets.

The total number of all infectious and cause-specific CSI visits will be collected using data available from the Niger Ministry of Health.

Refer to the Data Monitoring Plan for full details.

4.4.8.6. Statistical Analysis Plan

Refer to the trial's Statistical Analysis Plan (SAP) for complete details

4.4.8.7. Quality Control and Monitoring

All study personnel will be trained on standardized study procedures for data collection before each round of study activities begin. A team of supervisors in Niger will monitor study personnel collecting data weekly, and the UCSF team will conduct biannual study monitoring visits. Weekly data monitoring reports will be distributed to the study team, with a detailed section on inconsistencies, errors, and missing data to be resolved by the data teams.

4.5. Delivery Trial

4.5.1. Trial Design

From the same pool of eligible communities, 160 communities in Dosso will be randomly selected for participation in one of two Delivery Trials:

In Delivery Trial I, 80 communities in Dosso will be randomly selected for participation in a 2-arm trial that will compare coverage, costs, and other implementation outcomes between fixed-point delivery and door-to-door delivery of azithromycin by community health workers to children

1–59 months of age.

In Delivery Trial II, 80 communities in Dosso will be randomly selected for participation in a 2-arm trial that will compare costs and other implementation outcomes between treating eligible children 1–11 months of age to treating eligible children 1–59 months of age. The intervention delivery method for all communities will be informed by Delivery Trial I.

4.5.2. Eligibility

Intervention

At the community-level, eligibility includes:

Inclusion criteria:

- Location in Dosso
- Population 250 to 2,499*
- Distance > 5 km from district headquarters town
- Distinguishable from neighboring communities
- Verbal consent of community leader(s)

Exclusion criteria:

- Inaccessible or unsafe for study team
- “Quartier” designation on national census

*Population size as estimated from the most recent national census or projections

At the individual-level, eligibility includes:

Inclusion criteria:

- Age 1–59 months or 1–11 months depending on arm allocation
- Primary residence in a study community
- Verbal consent of caregiver/guardian for study participation

Exclusion criteria:

- Known allergy to macrolides

4.5.3. Randomization, Adaptation, and Masking

4.5.3.1. Randomization

The randomization unit for these trials will be the *grappe*, which is the smallest health care unit administratively defined in Niger, hereafter referred to as “community.”

Delivery Trial I communities will be randomized in a 1:1 fashion to the following 2 arms:

- **Azithro 1–59 fixed point:** azithromycin distribution to children 1–59 months of age using a fixed-point delivery approach via existing community health workers
- **Azithro 1–59 door-to-door:** azithromycin distribution to children 1–59 months of age using a door-to-door delivery approach via existing community health workers

Delivery Trial II communities will be randomized in a 1:1 fashion to the following 2 arms:

- **Delivery azithro 1–11:** azithromycin distribution to children 1–11 months of age using a door-to-door delivery approach via existing community health workers
- **Delivery azithro 1–59:** azithromycin distribution to children 1–59 months of age using a door-to-door delivery approach via existing community health workers

4.5.3.2. Adaptation

The randomization sequence will be generated by the trial biostatistician at UCSF.

The Delivery Trial communities will not undergo adaptation during the Delivery Trial period and may be included in the adaptation or programmatic trial after the Delivery Trial is completed.

Refer to the trial's Statistical Analysis Plan (SAP) for complete details of the adaptive randomization algorithm.

4.5.3.3. Masking

In the Delivery Trial, participants, implementers, and outcome assessors will not be actively masked from the delivery approach given the nature of the intervention. Data analysts will be masked.

4.5.3.4. Interventions

Please refer to Section 4.4.3.4.

4.5.3.5. Dosage Information

Azithromycin or placebo will be administered as a single dose, in oral suspension form for children, of 20 mg/kg in children (up to the maximum adult dose of 1 g).

In the Delivery Trials and Programmatic Trial, the dose will be calculated by age for children aged 1-11 months. Aged-based dosing will be implemented as follows:
- 2 ml of azithromycin for children aged 1-2 months
- 4 ml of azithromycin for children aged 3-11 months

For children 12-59 months of age, height-based dosing will be used via height-stick approximation as currently performed by Niger's trachoma program.

Both dosing cups and syringes will be used to administer treatment. For children too young to drink out of a dosing cup, a 1 ml or 5 ml syringe will be used, and the calculated dose will be rounded upwards to the nearest 0.2 ml.

4.5.3.6. Side Effects

Please refer to Section 4.4.3.6.

4.5.3.7. Study Medication Storage and Accountability

Please refer to Section 4.4.3.7.

4.5.3.8. Medication Quality Control

Please refer to Section 4.4.3.8.

4.5.4. Outcomes

4.5.4.1. Delivery Trial I Primary Outcomes

- Treatment coverage as defined by the number of doses of azithromycin administered and recorded by community health workers divided by the eligible population as determined by a post-distribution census in each community after a single distribution.

4.5.4.2. Delivery Trial I Secondary Outcomes

- Program costs as captured by routine administrative data collection during the study period and by micro-costing activities
- Cost-effectiveness measured by estimated program costs and using measured mortality and estimated DALYs as the effectiveness outcome, compared across arms.
- Estimated national-scale effects (health and cost) of scaling up each strategy.
- Other implementation outcomes defined in the Proctor Outcomes Framework,[13] including feasibility and acceptability, as captured during routine administrative and monitoring data collection during the study period and by stakeholder interviews at the participant, community, and health system levels after one round of distributions (Table 3)
- Treatment coverage as defined by projections using national and study census data collection as well as satellite imagery

4.5.4.3. Delivery Trial II Primary Outcomes

- Cost per dose delivered. Program costs will be captured by routine administrative data collection during the study period and by micro-costing activities. Doses delivered will be recorded by community health workers using paper data collection forms and entered electronically by the supervisor team.

4.5.4.4. Delivery Trial II Secondary Outcomes

- Treatment coverage as defined by the number of doses of azithromycin administered in each community divided by the total eligible population, which will be determined in several ways: using the recorded number of eligible children from the community health workers, using a pre-treatment census of eligible children, using self-reported coverage recorded during the post-treatment survey, and the administrative estimates of the eligible population from the health centers. The number of eligible children recorded by community health workers will be used for the main analysis with the others included as sensitivity analyses, and a separate analysis will be done to compare the 4 methods.
- Other implementation outcomes defined in the Proctor Outcomes Framework, including feasibility and acceptability, as captured during routine administrative and monitoring data collection during the study period and by stakeholder interviews at the participant, community, and health system levels after one round of distributions.

4.5.5. Sample size

Please refer to Section 4.4.5.1.

4.5.6. Recruitment

Before implementation begins in a region, a sensitization campaign will inform local health authorities and community leaders of the study design and nature of participation in the trial. In addition to IRB and national-, regional-, and district-level approval, community-level approval will be obtained before study activities begin in any community. All households will be recruited for participation in the census, and all children 1–11 or 1–59 months of age will be recruited for the intervention depending on the study arm their community has been randomized to. Household-level consent will be obtained for the census activities, and caregiver/guardian consent will be obtained for interventions and sample collection. Details of informed consent are found in Section 5.3.

4.5.7. Adverse Events Monitoring

Please refer to Section 4.4.7.

4.5.8. Data Collection, Management, Analysis, and Monitoring

4.5.8.1. Data Collection Overview

Delivery trial data will be collected electronically and on paper as described below.

4.5.8.2. Measurement of Implementation Outcomes

In the Delivery Trial, existing community health workers will be trained to distribute azithromycin to children 1–59 months of age or 1–11 months of age every 6 months. In communities randomized to fixed-point delivery, community health workers (CHWs) will work with community leaders to identify a central location, date, and time for delivery. Mobilizers in each community will be engaged to publicize the intervention. In communities randomized to door-to-door delivery, CHWs will visit every household in the community. In both sets of communities, CHWs will determine eligibility, obtain informed consent, administer treatment, and record treatment administration on paper data collection forms. For eligible children in Delivery Trial I, the dose will be determined via weight using a hanging scale for children under 12 months of age or height-stick approximation for children 12 months and older using the Niger trachoma program's height stick. For eligible children in Delivery Trial II, the dose will be determined via age for children under 6 months of age or height-stick approximation for children 6 months and older using the Niger trachoma program's height stick. Treatment coverage will be monitored by tracking the total number of children treated in each community as recorded by CHWs during distributions. Denominators for treatment coverage will be estimated through a post-distribution census conducted by trained census workers.

Program costs
Program costs will be captured through micro-costing (units of resources used multiplied by the

unit costs thereof). The economic approach will assign value to all items used, regardless of the payer, and regardless of whether the resources consumed are fully monetized. A macro-costing approach will be used for reconciliation and for understanding cost efficiency: overall program spending and outputs will be compared to the totals found via micro-costing. Costs will be collected using both routine project administrative data collected for all study communities (e.g., expenditure reports for services delivered, operational reports for distances travelled) with “program costs” being defined as costs needed to deliver the program in the absence of any research efforts. Research costs will be measured as part of the overall project but removed when comparing different age and delivery options to more accurately reflect an azithromycin MDA program. The *micro* and *macro* approaches are described in turn below. Finally, ‘start-up’ costs will be distinguished from ongoing running costs.

In addition to routine project administrative data collection, targeted additional data collection in the form of time and motion (T&M) studies for a small subset of communities will be implemented. As described below, the T&M studies will adapt and use forms that have been successfully used in other studies. Forms and data entry and analysis systems needed for routine program accounting, management and reporting purposes will be developed concurrently with the cost data collection needed for economic analysis and piloted using pre-implementation expenditures.

Program micro-costing

Cost information collected through regular project records will include the following categories:

- **Personnel:** Salaries and benefits (and any other emoluments, such as bonuses, etc.) for all staff, including service delivery (azithromycin distribution), program administration, transportation, etc.
- **Consumables:** Medications, office supplies, dosing cups, syringes, water, scales, height sticks, fuel, and supplies needed for AMR surveillance, including swabs, tubes, media, storage, shipment, and processing, etc.
- **Services:** Transportation, equipment maintenance, utilities, publicity, transport.
- **Equipment:** Vehicles, laboratory equipment, and other significant capital items.
- **Training:** Salaries, per diems, transport, food, etc.
- **Space:** Rental cost and/or purchase depreciation.

The last three items, equipment, training, and space will be appropriately amortized.

Costs will be allocated appropriately in collaboration with local project staff in order to ensure accurate division of project and research costs. Should the economics team find discrepancies, in whether a cost is research or project oriented, the team alongside local project staff will discuss until consensus is achieved. Services-related, non-research costs will then be allocated to the categories listed above and cross–categorized by particular activities. These activities include direct service delivery excluding supervision; field supervision; transportation, and overhead, among others. Expenditure data will be allocated to these activities by recall via structured interviews and, for a small subset of sites, via T&M data. These costs can then be allocated to the community level according to a schema consistent with the one developed for the provisional cost model and which reflects current budget items. Cost allocation will distinguish payer (government / program, volunteer/contributed) and location (community, district, region).

Time & Motion (T&M) studies

T&M will be conducted at least twice per region and distribution, for one week each time, for a sample of communities. The T&M activity has three purposes. First, it is one of the approaches we will use to compare the resources needed at the community level between the 1 – 11 month and 1 – 59-month distribution strategies and the fixed-point versus door-to-door strategies in the Delivery Trial. Second, the T&M data will inform modeling of intervention cost in settings with different characteristics. Third, the T&M data will help to disaggregate field costs associated with delivery of a real-world intervention from those related to the research setting. The T&M will quantify:

- Time spent on program activities vs. other responsibilities (if any).
- Within program activities, time spent at the community level (e.g. with a local leader), moving around the community, addressing household-level issues such as explanation and consent, and administering azithromycin to individual children.
- At both the community and the household level it will allow us to distinguish between fixed and variable costs.

Sampling for T&M will be conducted in order to provide robust estimates for important subgroups, including geographic regions, village size, difficult-to-reach villages, and other factors to be decided.

Cost Differentiation

Start-up costs. Large expenditures often occur in the early phases of a project when sizable one-time costs are incurred. This includes vehicle purchases, other equipment, and furniture purchases, building refurbishment, and some training costs.

Focus on items contributing to majority of costs. Previous experience suggests that total costs are disproportionately attributable to a relatively small number of items. Data collection will therefore be calibrated via a formal “forest and trees” process of identifying the limited number of inputs constituting 90 - 95% of costs. We will reduce efforts to obtain high precision for items comprising the other 5 - 10%.

Tracking costs over time. Unit costs may vary over time. For example, both administrative and field teams may become more efficient as they gain experience. We will generate graphs which display trends in major cost categories over time. We will also conduct structured interviews that aim to identify the proximate causes for observed changes in efficiency.

Program macro-costing

After removing research-related costs, program costs will be divided by the total number of outputs to arrive at a set of unit cost estimates. If these approximate the array of unit cost estimates obtained from the micro-costing approach described above, this would reconcile the micro-costing approach.

Cost-effectiveness analyses

Program costs and mortality outcomes will be used to develop incremental cost-effectiveness ratios (ICERs) for age group comparisons as detailed in the SAP.

Table 3 summarizes the collection of additional process indicators to track key implementation

outcomes using the Proctor Implementation Outcomes and RE-AIM frameworks, which will be applied across work streams for program monitoring and evaluation.

Table 3. Proctor Implementation Outcomes and RE-AIM Frameworks Applied to AVENIR

Outcome	Definitions	Sources of Data
Acceptability/Appropriateness	Perception of and satisfaction with intervention, facilitators and barriers to participation and implementation, relevance of intervention; at participant/community, implementer, and governmental levels	Semi-structured interviews, surveys
Reach/Penetration	Census coverage	Census, NSVRS
	Refusals and reasons for refusals	Census, administrative data
	Treatment coverage, equity of coverage by sex, age, geography	Census
Cost	Program costs	Administrative data, micro-costing
	Cost-effectiveness	Administrative data, census, micro-costing
Feasibility/Fidelity	Timeliness and fidelity of intervention rollout	Administrative data, site visits
	Training and supervision metrics, staff retention	Administrative data, site visits
	Drug storage, temperature, inventory, delivery monitoring	Administrative data
	AMR sample viability, rejection rates, processing speed	Administrative data
Sustainability	Above metrics compared over time	See above

4.5.8.3. Data Collection and Management

Please refer to section 4.4.8.5.

4.5.8.4. Statistical Analysis Plan

Please refer to section 4.4.8.6.

4.5.8.5. Quality Control and Monitoring

Please refer to section 4.4.8.7.

4.6. Programmatic Trial

4.6.1. Trial Design

The remaining 1,500 eligible communities will participate in a Programmatic Trial that utilizes existing community health workers working with the Niger Ministry of Health community health program. The distribution of biannual azithromycin will begin approximately 1.5 years after the first Mortality Trial enrollment and will be offered to children aged 1–11 months in selected CSIs. CSIs will be used as the randomization unit. CSIs will be randomized in a 2:1 allocation to azithromycin distribution to children aged 1–11 months by community health workers or no azithromycin distribution / usual care by community health care workers, with the allocation favoring azithromycin. AMR after 1 year will be monitored at the community level using similar methods to the Mortality/Resistance Trial as well as in children presenting to selected CSIs with diarrheal disease, respiratory infection, or for a well-child visit. Mortality will be monitored by birth histories after 2 years of treatment distribution.

After 1 year of distributions, the results from the Mortality/Resistance trial will be available and will be used to inform the next steps for the Programmatic Trial. The trial may continue based on the Mortality trial results, the availability of funding, and stakeholder preferences. If the study continues, additional regions will be included in the Programmatic Trial (Figure 2), using adaptive randomization as described for the Mortality/Resistance Trial. The original Mortality/Resistance Trial communities would also be included in the Programmatic Trial during this expansion. Mortality, resistance, and clinic visits will be monitored every 2 years. In this platform trial, additional treatment arms may be added to the trial or arms may be dropped from the trial based on additional information from other studies, updates to the WHO guidelines, and preferences from programs and policymakers in Niger. With such changes, protocol details, power calculations, and analysis plans will be updated accordingly in the protocol and statistical analysis plan.

4.6.2. Eligibility

<u>**Intervention**</u>

At the CSI-level, eligibility includes*:

<u>*Inclusion criteria:*</u>

- Location in Dosso, Tahoua, Maradi, Zinder, or Tillabéri regions
- Verbal consent of community leader(s)

Exclusion criteria:

- Inaccessible or unsafe for study team
- “Quartier” designation on national census

* Community-level eligibility for the programmatic trial will be based on Ministry of Health community health worker program guidelines and may change from what is currently listed above.

At the individual-level, eligibility includes:

Inclusion criteria:

- Age 1–11 months
- Primary residence in a study CSI
- Verbal consent of caregiver/guardian for study participation

Exclusion criteria:

- Known allergy to macrolides

Population-based sample collections

At the community-level, eligibility includes:

Inclusion criteria:

- Location in study region
- Distinguishable from neighboring communities
- Verbal consent of community leader(s)

Exclusion criteria:

- Inaccessible or unsafe for study team
- Not randomly selected
- Received treatment prior to sample collection

At the individual-level, eligibility includes:

Inclusion criteria:

- Age 1–59 months
- Primary residence in a study community selected for sample collections
- Written consent of caregiver/guardian for study participation

Exclusion criteria:

- Not on list of randomly selected participants from the census

CSI-based sample collections

At the CSI-level, eligibility includes*:

Inclusion criteria:

- Randomly selected CSI for inclusion within the Programmatic Trial
- Verbal consent of CSI leader(s)

Exclusion criteria:

- Not randomly selected for participation in the Programmatic Trial

*CSI-level eligibility for the programmatic trial will be based on Ministry of Health community health worker program guidelines and may change from what is currently listed above.

At the individual-level, eligibility includes:

Inclusion criteria:

- Presents to CSI with symptoms of diarrheal disease, respiratory illness, or visiting for routine healthcare (e.g. vaccination)
- Verbal consent of caregiver/guardian for study participation

Exclusion criteria:

- Presents to CSI with disease symptoms not related to diarrheal or respiratory disease
  Received study treatment prior to sample collection

4.6.3. Randomization, Adaptation, and Masking

4.6.3.1. Randomization

The randomization unit for these trials will be the CSI. Each CSI includes a catchment area of approximately 10 communities.

CSIs will be randomized in a 2:1 allocation favoring azithromycin to the following 2 arms:

- **Programmatic azithro 1–11:** all children aged 1–11 months will be offered a biannual dose of azithromycin. This distribution will be provided by existing community health workers as part of the Ministry of Health's community health program.
- **Programmatic no intervention:** All children aged 1–11 months will not receive azithromycin within the framework of this study. However, all children in this study will receive other health services provided by community health workers as part of the Ministry of Health's community health program.

4.6.3.2. Adaptation

The randomization sequence will be generated by the trial biostatistician at UCSF

The Programmatic Trial will use an adaptive allocation modeled after the adaptive allocation employed in the Mortality/Resistance Trial. Communities will undergo the first randomization in a 2:1 allocation favoring the Programmatic azithro 1–11 arm. If the program continues after the first year, new communities will be enrolled using the same adaptation methods used for the Mortality Trial.

After the Mortality Trial's primary outcome analysis, the study team and key stakeholders will determine whether the Programmatic Trial continues. If the study continues, the Mortality Trial communities will be included in the Programmatic Trial and additional regions will be added over time (Figure 2). In this platform design, additional treatment arms may be added to the trial or arms may be dropped from the trial based on additional information from other studies, updates to the WHO guidelines, or preferences of programs or policymakers in Niger. With such

changes, protocol details, power calculations, and analysis plans will be updated accordingly in the protocol and statistical analysis plan.

Refer to the trial's Statistical Analysis Plan (SAP) for complete details of the adaptive randomization algorithm.

4.6.3.3. Masking

In the Programmatic Trial, participants, and implementers will not be actively masked from the study arm allocation given that no placebo will be used. Outcome assessors and data analysts will be masked.

4.6.3.4. Interventions

In the Programmatic Trial, existing community health workers employed by the Minister of Health will be utilized. Community health workers from randomly selected, eligible communities will be trained to distribute azithromycin to children 1–11 months of age from within randomly selected CSIs every 6 months for a period of 1 year, with distributions potentially continuing beyond 1 year dependent on funding.

In all communities in the Programmatic Trial, community health workers will continue to distribute routine care services, including prevention, screening, and treatment of diseases (such as malnutrition, malaria, diarrhea, and pneumonia), referral to the health center, nutritional advice, vaccination, or administration of vitamin A, as per the Ministry of Health community health program.

Please refer to Section 4.4.3.4 for more details on the intervention.

4.6.3.5. Dosage Information

Please refer to Section 4.5.3.5.

4.6.3.6. Side Effects

Please refer to Section 4.4.3.6.

4.6.3.7. Study Medication Storage and Accountability

Please refer to Section 4.4.3.7.

4.6.3.8. Medication Quality Control

Please refer to Section 4.4.3.8.

4.6.4. Outcomes

4.6.4.1. Primary Outcomes*

- Number of infectious cause CSI visits per month for children aged 1–59 months over 1 year
- Number of cause-specific CSI visits (diarrhea, respiratory infections, and malaria) per

month for children 1–59 months of age over 1 year

*If the Programmatic Trial continues, outcomes will be assessed every 2 years

4.6.4.2. Secondary Outcomes

- Prevalence of genetic determinants of macrolide resistance in nasopharyngeal swabs in children aged 1–59 months from population-based samples after 2 years of distribution
- Load of genetic determinants of macrolide resistance in rectal swabs among children aged 1–59 months from population-based samples after 2 years of distribution
- Prevalence of genetic determinants of macrolide resistance in nasopharyngeal swabs in children aged 1–59 months with respiratory illness from CSI-based samples after 2 years of distribution
- Load of genetic determinants of macrolide resistance in rectal swabs among children aged 1–59 months with diarrheal disease from CSI-based samples after 2 years of distribution
- Infant- and under-5 mortality rate (deaths per 1,000 livebirths) 2 years after the first treatment as assessed via birth history

  Program costs as captured by routine administrative data collection during the study period and by micro-costing activities
- Estimated national-scale effects (health and cost) of scaling up each strategy.

  Other implementation outcomes defined in the Proctor Outcomes Framework,[13] including feasibility and acceptability, as captured during routine administrative and monitoring data collection during the study period and by stakeholder interviews at the participant, community, and health system levels after one round of distributions (Table 3)
- Treatment coverage as defined by projections using national and study census data collection as well as satellite imagery

Microbiome outcomes for rectal swab collections

- Gamma diversity, estimated as the alpha diversity of the pooled community sample using the Shannon diversity index
- Overall microbial structure using Manhattan distance
- Relative abundance of individual microbial taxa at genus and species level

4.6.5. Sample size

4.6.5.1. Overview

Please refer to Section 4.4.5.1.

4.6.5.2. Sample Collections

In the Programmatic Trial, sample collection will take place in communities in the catchment area of 40 randomly selected CSIs (20 CSIs per arm) and at the same 40 selected CSIs themselves. Additional details about this collection can be found in Section 4.10.3, *Measurement of Sample Based Outcomes.*

In all communities selected for sample collections, treatment distribution for that round will be conducted after sample collections.

Refer to SAP for details of power calculations.

4.6.6. Recruitment

Before implementation begins in a region, a sensitization campaign will inform local health authorities and community leaders of the study design and nature of participation in the trial. In addition to IRB and national-, regional-, and district-level approval, community-level approval will be obtained before study activities begin in any community or CSI. All children aged 1–11 residing in a CSI catchment area randomized to receive treatment will be recruited for the intervention. Household-level consent will be obtained for the census activities, and caregiver/guardian consent will be obtained for interventions and sample collection. Details of informed consent are found in Section 5.3.

4.6.7. Adverse Events Monitoring

Please refer to Section 4.4.7.

4.6.8. Data Collection, Management, Analysis, and Monitoring

4.6.8.1. Data Collection Overview

Programmatic Trial data will be collected electronically and on paper as described below.

4.6.8.2. Measurement of Mortality Outcomes

For communities randomized to participate in the Programmatic Trial, mortality will be monitored through an annual door-to-door birth history survey in a subset of randomly selected communities using trained census workers and following similar methods as described above. All women aged 12-65 will be invited to participate and asked about the status of each live birth they experienced over the past 10 years, including the name, age, sex, date of birth, current status, and as needed, date of death.

4.6.8.3. Measurement of Sample Based Outcomes

In the Programmatic Trial, sample collection will take place within communities in the catchment area of 40 selected CSIs (20 CSIs per arm) and at the same 40 selected CSI locations.

- For samples collected within communities (population-based):
  - 3 communities per CSI from each of the 40 CSIs (120 communities total) will be randomly selected from the pool of eligible communities and followed to monitor antimicrobial resistance. In each community, 10 children aged 1–59 months will be selected to participate in nasopharyngeal and rectal swab sample collections. One rectal swab and one NP swab will be collected from each child.

- For samples collected at CSI locations (CSI-based):
  - 1 rectal swab will be collected from the first 30 children who present to the CSI with symptoms of diarrheal disease.
  - 2 nasopharyngeal swabs will be collected from the first 30 children who present to the CSI with symptoms of respiratory disease. The second nasopharyngeal swab collected will be sent to the CERMES lab for processing.

- 1 rectal swab and 2 nasopharyngeal swabs will be collected from the first 30 children who for routine medical care (e.g. vaccination) and present with no disease symptoms. The second nasopharyngeal swab collected will be sent to the CERMES lab for processing.

In the Programmatic Trial, population-based sample collections will follow similar procedures to those described above for the Mortality/Resistance Trial. CSI-based collections will take place in the same month as the community-based collections for that CSI, with a window of + or – 1 month. The presence of diarrheal or respiratory illness will be determined using the UNICEF Integrated Management of Childhood Illness flowchart currently used in Niger. Eligible children will be consecutively enrolled until the target sample sizes are achieved. A copy of the Integrated Management of Childhood Illness flowchart that will be used can be found in the Section 7.12 of the appendix.

To randomly select individuals for population-based monitoring in the Programmatic Trial, a "random walk" approach will be used.

Random walk selection protocol

1. Start at the home of the chef du village.
2. Use the application in CommCare to determine how many steps to go and in which direction to walk.
3. Approach the household that is closest to you when you finish your steps.
4. Ask the household if there are children 1-59 months of age and if the household is willing to participate. If not, choose the next closest household and repeat step 4.
5. Enter the number of eligible children 1-59 months in the household into the app, and use the app to select which child from which to take samples.
6. Take samples and complete data collection form.
7. Repeat steps 2-6 until you have completed 10 households.

For both trials with sample collections (the Mortality/Resistance Trial and Programmatic Trial), once the census is complete and the sample is generated, the study team will work with a local mobilizer to invite the sampled group to a central location in the community for sample collection. Samples will be collected by trained personnel and linked to census data through bar codes. Once sample collection is complete, the census team or community health workers will visit the study community to complete treatment for that round.

During collection, samples will be stored at ambient temperature or on ice at 4°C. At the end of each collection period, samples will be transported to a location near the sampled community with facilities to store samples at -20°C as required or directly to CRISP each day. At the end of the collection period, all samples will be transferred to a central storage location at CRISP in Niamey stored at -20°C as required until processed or shipped to UCSF for processing. Refer to Sample Collections Procedures and the Laboratory Procedures for full details.

4.6.8.4. Measurement of Clinic Visit Outcomes

Clinic visits will be monitored through passive surveillance at CSIs in Programmatic Trial study regions. All infectious cause visits and cause-specific visits for diagnoses of respiratory infection, diarrheal disease, and malaria will be recorded among children under 5 based on the age groups routinely reported in Niger. Visits will be measured continuously by CSI staff using Niger's DHIS2 national system for health information database. Records will be regularly reviewed for eligible entries and data including child age, community of residence, date of visit,

diagnosis at visit, and treatment given will be extracted electronically.

The presence of respiratory infection, diarrheal disease, and malaria are determined using the UNICEF Integrated Management of Childhood Illness flowchart currently used in Niger. A copy of this Integrated Management of Childhood Illness flowchart can be found in the Section 7.12 of the appendix.

4.6.8.5. Measurement of Implementation Outcomes

Implementation-focused outcomes include costs, cost-effectiveness, and the additional outcomes outlined in the Proctor Implementation Outcomes and RE-AIM frameworks (Table 3).

4.6.8.6. Data Collection and Management

Please refer to Section 4.4.8.5.

4.6.8.7. Statistical Analysis Plan

Refer to the trial's Statistical Analysis Plan (SAP) for complete details

4.6.8.8. Quality Control and Monitoring

Please refer to Section 4.4.8.7.

5. Ethics and Dissemination

5.1. Overview

The trial will be conducted according to Good Clinical Practice guidelines (ICH-GCP) and will adhere to the principles of the Declaration of Helsinki. Ethical approval will be obtained at all levels described below before study activities begin.

5.2. Institutional Review Board Approval

5.2.1. UCSF IRB

The University of California, San Francisco Committee on Human Research will annually review the study protocol for ethical approval. The UCSF IRB number on record is, UCSF IRB 19-28287

5.2.2. Comité national éthique pour la recherche en santé (CNERS)

The study protocol will be reviewed and granted ethical approval by CNERS before any study activities begin. The CNERS IRB number on record is DELIBERATION N°041/2020/CNERS.

5.3. Informed Consent

In addition to IRB approval at the national-, regional-, district-, and community-level approval will be obtained before study activities begin. The study team will meet in person with regional, district, and community leaders to explain the nature of the study, answer questions, and obtain verbal or written consent, which will be documented electronically.

After verbal consent from the regional, district, and community leaders is obtained, the study team will recruit participants by going door to door in each community enrolled in the study. The team will approach the head of household, explain the nature of the study, answer questions, and obtain verbal consent to perform the census. If the head of household agrees to participate, caregivers/guardians of children under 5 years old will be approached for verbal consent to treat the child. For children 30 to 42 days old, written consent will be obtained via caregiver thumbprint. The consent process will be conducted by trained personnel in relevant local languages and witnesses will be used to verify the process for illiterate community members. The consent process will also include the possibility for the household to be randomly selected for examination if the household resides in one of the randomly selected communities in Dosso region.

5.4. Potential Risks and Adequacy of Protection Against Risks

Azithromycin is usually well tolerated. The most common side effects are diarrhea, nausea, abdominal pain, and vomiting. These side effects occur in fewer than 5% of persons who receive azithromycin and have been rarely reported in studies of the mass distribution of azithromycin for trachoma or child mortality. Adverse events will be monitored as described in Section 4.10.

Azithromycin is thought to be safe in children under 6 months of age and is recommended by the CDC as the treatment of choice for prophylaxis against whooping cough (Bordetella pertussis) in this young age group. There is a possibility of an increased risk of infantile hypertrophic pyloric stenosis in neonates. To mitigate this risk, the trial will exclude children under 1 month of age.

5.5. Compensation for Participants

There is no cost to the participant and there is no reimbursement for overall participation in this study.

5.6. Dissemination Plan

Trial results will be presented at local and international meetings and submitted to peer-reviewed journals for publication.

6. Organizational Structure and Responsibilities

6.1. University of California, San Francisco Francis I. Proctor Foundation

The Proctor Foundation is an organized research unit at the University of California, San Francisco. Proctor has a 60-70-year history of research in ocular infectious and inflammatory diseases and runs one of the leading corneal and uveitis fellowship training programs in the United States. Proctor Foundation faculty have been involved in the prevention of blindness research in developing countries since the foundation's inception. The impetus for establishing the foundation in 1947 was to eradicate trachoma in the American Southwest and other parts of the world.

From this initial inception, the Foundation has expanded research efforts to include the other major causes of blindness worldwide, with a continuing emphasis on infectious and inflammatory eye diseases. The Proctor Foundation will be the main coordinating center for the study. Thomas Lietman, the Principal Investigator of this study at the Proctor Foundation, will be assisted by Kieran O'Brien, the co-Principal Investigator, several co-investigators, two biostatisticians, and several study coordinators, and data analysts.

6.2. Programme national de santé oculaire (PNSO)

Le Programme National de Santé Oculaire PNSO, was established in December of 2012. Before this time, the program existed under the name of the Programme National de Lutte Contre la Cécité (PNLCC), which was created in December of 2004 as part of the *Direction de la lutte contre la maladie lié à la Direction Générale de la Santé Publique*. PNSO is currently associated with the *Direction des Etudes et de la Programmation*, a part of the larger Ministry of Health in Niger (Secrétariat Général du Ministère de la Santé Publique [MSP].

PNSO is located in Niamey, Niger. However, it also has operations in the East under the Direction Nationale des Immunisations, in the West under the Lycée Bosso in the North under the quartier recasement and in the South under the Mairie Commune I.

PNSO is tasked with the coordination and evaluation of Nigerien national ocular health and has support mass drug administration efforts for decades.

6.3. Division santé communautaire (DSC)

The goal of the DSC is to contribute to the reduction of infant, child and maternal mortality through health promotion, improved access to care for remote communities and improved utilization of health services with community participation. The DSC is currently associated with the *Direction de l'organisation des soins*, a part of the larger Ministry of Health in Niger. The current director is Dr. Amadou Zakari.

The DSC will work with the study team to integrate the Programmatic Trial into the existing community health worker framework being implemented throughout Niger.

6.4 Centre de recherche medical et sanitaire (CERMES)

The CERMES laboratory is a Nigerien public establishment for science and technology. It has been a part of the international network of Pasteur Institutes since 2003. The center has a three-fold mission of promoting research, public health and education.

6.5. Centre de recherche et interventions en santé publique (CRISP)

CRISP is a business entity founded by Ahmed Arzika based in Niamey, Niger. CRISP will work with UCSF and PNSO to implement the study.

6.6. Centres de santé intégré (CSI)

CSIs are community health posts put in place by the Nigerien Ministry of Health. Agents employed by the CSIs will work at the grappe level to deliver treatment and record adherence in the delivery trial.

6.7. Executive Committee

The Executive Committee will consist of Thomas Lietman and Kieran O'Brien from the Proctor Foundation and Ahmed Arzika from CRISP. This committee will act as the administrative and executive arm of the clinical trial and will meet in person twice a year to provide overall oversight for the study and make decisions on day-to-day operation issues, including:

- Monitor study progress and data collection progress
- Discuss quality control issues that have arisen in the Trial Coordinating Center (TCC) and Data Coordinating Center (DCC)
- Evaluate and adopt changes in study procedures as necessary
- Communicate with and implement recommendations from the Data and Safety Monitoring Committee (DSMC)
- Make executive decisions on the allocation of resources
- Establish policies on publications and authorship
- Approve and oversee ancillary studies

6.8. Trial Coordinating Center (TCC)

The TCC will be located at the Proctor Foundation. Thomas Lietman will lead the center, which will also include Kieran O'Brien (co-PI), Travis Porco (biostatistician), Benjamin Arnold (biostatistician), Jeremy Keenan (co-investigator), Catherine Oldenburg (co-investigator), Victoria Lee (data analyst), Zijun Liu (data analyst), and Catherine Cook (study coordinator). The role of the TCC will be to oversee and coordinate the overall implementation of the trial. Specifically, this includes maintaining an up-to-date study protocol and manual of procedures, obtaining ethical approvals from all involved parties (UCSF, Niger MOH), conducting training and certification of all study personnel, ensuring proper masking of outcome assessment, and monitoring to ensure that protocols of the study intervention are applied correctly in the field. The TCC will organize site visits at least once per year before each monitoring visit to conduct training sessions with outcome assessors and monitor the quality of data collection. The Proctor study coordinator(s), with assistance from the CRISP study coordinators, will organize the training sessions. The TCC will meet officially as a group at least four times per year. All members of the TCC are currently working on studies of childhood mortality in Niger and Burkina Faso. The group has close working relationships with the CRISP staff in Niger and expertise with cluster-randomized trials in resource- poor settings.

6.9. Niger Coordinating Center

The Niger Coordinating Center will be located at the CRISP headquarters in Niamey, Niger. The center will be led by the study site principal investigator, Ahmed Arzika, and also include the CRISP study coordinator. Ahmed Arzika will oversee the study activities that take place in Niger

and will manage the day-to-day activities of the study coordinator. He will also assist with obtaining ethical approval from appropriate federal, regional, and zonal agencies. The CRISP study coordinator will work with the local health officials to find census workers for the census activities and nurses to work as data collectors. This person will supervise all data collection activities and will be responsible for proper storage and transport of specimens.

6.10. Data Coordinating Center (DCC)

The DCC will be located at the Proctor Foundation and led by Benjamin Arnold and Travis Porco. The Proctor Foundation has served as a DCC for several other trials, including the cluster-randomized trials of trachoma in Ethiopia (TANA, NEI U10 EY016214) and Niger (MORDOR; Gates OPP 1032340), and individual- and cluster-randomized trials of corneal ulcer treatment and prevention in India (SCUT, NEI U10 EY015114; MUTT, NEI U10 EY018573) and Nepal (VIEW, NEI U10 EY022880). Ben Arnold and Travis Porco, along with Zijun Liu and Victoria Le (data analysts) will be responsible for data management, data quality control, event adjudication, and training and certification of enumeration personnel. The DCC will be responsible for drafting the trial's statistical analysis plan, generating the trial's randomization sequence, analyzing data according to the plan, and providing data requested for publications. The DCC will be responsible for coordinating and supervising the activities of the Data and Safety Monitoring Committee, including preparing interim and final data reports. The DCC will also be responsible for coordinating the use of the electronic data capture system, including maintenance of the software application and data backup monitoring visits. The DCC will meet monthly. The DCC will be in close contact with the Proctor study coordinator(s), who will help address any issues with counterparts in Niger.

6.10.1. Data Safety and Monitoring Committee (DSMC)

The DSMC is comprised of independent experts in bioethics, biostatistics, epidemiology, antimicrobial resistance, and global health. The DSMC will meet at least once per year. Ad hoc meetings may be convened as necessary. The DSMC will approve the trial protocol at the first meeting or recommend modifications to the protocol as necessary. At annual meetings, the DSMC will review data on efficacy and safety outcomes. They will also monitor for unanticipated events.

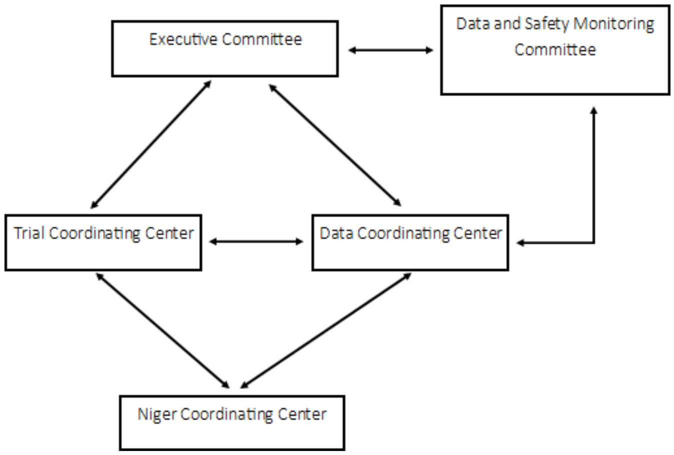

6.11. Economic Consultants

The AVENIR team will work with Dr. Meagan C. Fitzpatrick from the University of Maryland School of Medicine. Dr. Fitzpatrick will support the development of the sampling strategy,

protocol, data collection forms, and analysis plan for all economic-focused outcomes.

6.12. Duties and Responsibilities of Staff

6.12.1. Principal Investigator and Co-Principal Investigators (Proctor Foundation)

- Develop study design and specific aims with help of biostatisticians, study coordinators, and partners
- Obtain grant funding and develop grant budget with help of partners
- Ensure execution of the study according to the protocol
- Ensure that ethical approvals are maintained
- Write or add major contributions to all study-related publications
- Ensure proper masking is maintained
- Supervise training and implementation of study procedures for all study staff
- Assume responsibility for the study in the absence of the Principal Investigator (Co- Principal Investigators)

6.12.2. Biostatisticians (Proctor Foundation)

- With the Investigators, create the Statistical Analysis Plan (SAP)
- Conduct all randomization allocations
- Communicate randomization allocations to Pfizer
- Receive all study data and review for quality control purposes
- Ensure appropriate masking
- Prepare data analysis plan for DSMC meetings; oversee analysis and prepare all presented data for DSMC meetings, DSMC reports, and all study publications

6.12.3. Study Coordinators (Proctor Foundation)

- Ensure the execution of the study per protocol
- Coordinate with implementing partners, particularly with the CRISP Study Coordinators, in execution of the study
- Manage correspondence between all collaborating organizations and parties
- Maintain all ethical approvals for the study, including IRB renewals, the authorization of the CNERS, the authorization of the BUA and DSMC-related approvals
- Prepare all forms and documents necessary for fieldwork (training materials, data collection forms, etc)
- Train census workers in the use of electronic data capture system
- Train both Proctor and Nigerien teams for each site visit
- Arrange logistics and itineraries for traveling team members in Niger
- Purchase, maintain, and organize transport of all necessary study supplies to/within Niger
- Maintain communication with Principal Investigators regarding all study activities and plans

6.12.4. Study Coordinators (Niger)

- Prior to the start of the study, secure support from the Ministry of Health and necessary ethical approvals in Niger
- At baseline, obtain permission from the district health leaders; meet with and obtain permission/consent from all community leaders and local stakeholders
- Supervise and train all study team members

- Charge all mobile devices and battery packs each night of the census
- Maintain all fieldwork documents and records
- Complete collection and intervention reports
- Oversee transport of study samples
- Assist Proctor Coordinators in coordinating logistics in Niger
- Work with DCC and data teams to maintain database
- Analyze and provide data when requested by co-investigators or staff from Proctor
- Appropriately back up all data

6.12.5. Data Analysts (Proctor Foundation)

- Assist with preparation of the Statistical Analysis Plan
- Develop data collection forms and database(s)
- Assist with training of study teams on using data collection forms
- Assist with training of Niger data team on monitoring data entry for quality and accuracy
- Conduct daily review of incoming data to monitor for quality and completeness
- Communicate with study coordinators and Niger data team about data discrepancies
- Prepare weekly data monitoring and study progress reports to share with study team
- Prepare reports summarizing data to be distributed to Data and Safety Monitoring Committee
- Conduct analyses and prepare tables and figures for reports, publications, and presentations

6.12.6. Data Analysts (Niger)

- Conduct daily review of incoming data to monitor for quality and completeness
- Work with Proctor Foundation study team to resolve discrepancies
- Assist data collection team with preparation and maintenance of mobile devices and application for use in data collection and with troubleshooting the application

6.12.7. Census and Treatment Team (PNSO)

- Before each census and treatment round, complete training
- During census and treatment rounds, travel to the enrolled communities for data collection
- Obtain informed consent from households and caregivers for study participation
- Administer study medications to eligible subjects according the the protocol
- Record consent, census, and treatment data in the mobile application
- Return to households until they have distributed study medications to at least 80% of individuals
- Counsel and motivate participants for follow-up and monitoring visits
- Inform participants of available health care facilities and procedures
- Collect information on the nature of adverse events experienced by study participants, and report this information immediately to CRISP investigators

6.12.8. Sample Collection Team

- Before each collection, complete training
- Prepare all study-related materials before travel to study sites
- In each cluster, mobilize and identify all randomly selected participants
- Obtain informed consent for enrollment from each participant or participant guardian
- Under supervision of CRISP Study Coordinator and/or trained Proctor staff, collect all

participant study samples according to protocol; store and record all samples correctly for transport, organization, and processing
- Counsel and motivate participants for follow-up and monitoring visits
- Inform patients of available health care facilities and procedures
- Collect information on the nature of any adverse events experienced by study participants, and immediately report to CRISP investigators

6.12.9. Members of the Programme national de santé oculaire (PNSO)

- Import study drug (azithromycin and placebo)
- Import study supplies as a donation from the United States to Niger
- Oversee a pre-study mapping activity for the purpose of determining the location of study communities
- Support pre-study sensitization and obtain community consent for the trial
- Assist with training and supervision of study staff located in Niger, including census workers and AMR sample collection team
- Liaise with relevant regulatory bodies and stakeholders, including the Nigerien ethics committee (CNERS), including securing ethical approval to conduct study activities throughout the duration of the study
- Oversee the building of study drug storage locations at the district level
- Assist with training, supervision and implementation of pre-study and pilot activities including baseline community-based sample collection in Dosso Region
- Oversee and provide administrative support for shipping of environmental sewage samples to test for antimicrobial resistance
- Manage hiring of data analysts/managers and agents for pre-study and pilot activities

6.13. Data Safety and Monitoring Committee

6.13.1. Primary Responsibilities of the DSMC

The DSMC will be responsible for safeguarding the interests of trial participants, assessing the safety and efficacy of the interventions during the trial, and monitoring the overall conduct of the trial. The DSMC will provide recommendations about stopping or continuing the trial. To contribute to the integrity of the trial, the DSMC may also formulate recommendations relating to the selection/recruitment/retention of participants, to the study protocol, and the procedures for data management and quality control.

The DSMC will be advisory to the Executive Committee. The Executive Committee will be responsible for promptly reviewing the DSMC recommendations and determining whether to continue or terminate the trial, and whether amendments to the protocol are required. If needed, the DSMC may seek the advice of a content expert outside of the committee.

6.13.2. DSMC Membership

The DSMC is an independent multidisciplinary group consisting of epidemiologists, biostatisticians, bioethicists, and clinicians that collectively has experience in pediatric infectious diseases and mortality, antimicrobial resistance, and in the design, implementation, and analysis of randomized controlled trials.

6.13.3. Conflict of Interest

The DSMC membership has been restricted to individuals free of apparent conflicts of interest. The source of these conflicts may be financial, scientific, or regulatory. Thus, neither study investigators nor individuals employed by the sponsor, nor individuals who might have regulatory responsibilities for the trial products, are members of the DSMC.

The DSMC members will disclose to fellow members any consulting agreements or financial interests they have with the sponsor of the trial, with the contract research organizations, or with other sponsors having products that are being evaluated or that are competitive with those in the trial. The DSMC will be responsible for deciding whether these consulting agreements or financial interests materially impact their objectivity.

The DSMC members will be responsible for advising fellow members of any changes in any of the membership requirements that occur during the course of the trial. It may be appropriate for DSMC members who develop significant conflicts of interest resign from the DSMC.

DSMC membership is to be for the full duration of the trial. If any members leave the DSMC, the Executive Committee, in consultation with the DSMC, will promptly appoint a replacement.

6.13.4. Timing and Purpose of DSMC Meetings

The initial meeting of the DSMC will be held before study activities begin. The committee will provide an advisory review of scientific and ethical issues relating to study design and discuss the standard operating procedures and analysis plan, as well as the format and content of the reports that will be used to present trial results.

The initial meeting will be attended by all DSMC members, lead trial investigators, and the trial biostatisticians. The DSMC will review drafts of the trial protocol, the Statistical Analysis Plan, and the DSMC Charter. At subsequent meetings, committee members will receive reports on study conduct, data collection, and adverse events.

6.13.5. Procedures to Ensure Confidentiality and Proper Communication

To enhance the integrity and credibility of the trial, procedures will be implemented to ensure the DSMC has access to emerging information from the trial regarding efficacy and safety as required.

6.13.6. Closed Sessions

Sessions involving only DSMC members (and, once primary outcome results become available, those unmasked trial investigators on the Data Coordinating Committee who will generate the unmasked reports), will be held to allow discussion of confidential data from the trial, including information about the efficacy and safety of interventions, as required. As this study uses an adaptive allocation design, there will be no interim unmasked data presented before primary outcome data become available. At each meeting after the initial meeting, at least two Closed Sessions will be scheduled.

At the final Closed Session of each meeting, the DSMC will develop a consensus on its list of recommendations, including whether the trial should continue.

6.13.7. Open Sessions

The study team will review masked information relating to study progress and data collection with

the DSMC members between the Closed Sessions.

6.13.8. Open and Closed Reports

As needed, Open and Closed Reports will be provided for each DSMC meeting. Open Reports will include data on recruitment and baseline characteristics, eligibility, and completeness of follow-up and compliance in aggregate. The DCC will prepare these Open Reports.

Closed Reports, available only to those attending the Closed Sessions of the meeting, will include analyses of primary and secondary efficacy endpoints, including subgroup and adjusted analyses, adverse events, and Open Report analyses that are displayed by intervention group. Closed Reports will be prepared by the unmasked study biostatistician and data analyst. No unmasked interim data will be reviewed during closed reports until primary analysis data has become available.

The Open and Closed Reports should summarize data collection that has been completed by at most two months before the date of the DSMC meeting. The Reports should be provided to DSMC members at least three days prior to the date of the meeting.

6.13.9. Minutes of the DSMC Meetings

The research team will prepare minutes for the open portion of the meeting, including the DSMC's recommendations.

6.12.10. Recommendations to the Executive Committee

At each meeting, the DSMC will make a recommendation to the Executive Committee to continue or terminate. This recommendation will be based primarily on safety and efficacy considerations and will be guided by statistical monitoring guidelines defined in this Charter.

Recommendations to amend the protocol or conduct of the study made by the DSMC will be considered and accepted or rejected by the Executive Committee. The Executive Committee will be responsible for deciding whether to continue or to stop the trial based on the DSMC recommendations.

The DSMC will be notified of all changes to the study protocol or to study conduct. The DSMC concurrence will be sought on all substantive recommendations or changes to the protocol or study conduct prior to implementation.

The Executive Committee may communicate information in the Open Report to the sponsor and may inform them of the DSMC recommended alterations to study conduct or early trial termination in instances in which the Executive Committee has reached a final decision agreeing with the recommendation. The Executive Committee will maintain confidentiality of all information it receives other than that contained in the Open Reports until after the trial is completed or until a decision for early termination has been made.

6.14. Policy Matters

Any changes to the protocol made during the course of the study will be incorporated into the revised study protocol and procedures and recorded in a change log. Any new forms will be incorporated as an addendum. All protocol changes will be submitted and approved by the IRB

of both collaborating centers as required. Major changes will also be submitted to DSMC.

6.14.1. Presentations and Publications

All presentations and publications will include acknowledgement of funding sources and give credit to the collaborating organizations and/ individuals involved.

Acknowledgements will include grant source(s) as well as the DSMC.

7. Appendices

Refer to Procedures for full documentation:

7.5. Informed Consent Forms
7.6. Mortality/Resistance Trial: Training, Procedures, Data Collection Forms
7.7. Delivery Trial: Training, Procedures, Data Collection Forms
7.8. Sample Collection: Training, Procedures, Data Collection Forms
7.9. Laboratory Procedures
7.10. Data Monitoring Plan
7.11. Adverse Events Monitoring Plan
7.12. Integrated Management of Childhood Illness flowchart

References

1. GBD 2016 Mortality Collaborators. Global, regional, and national under-5 mortality, adult mortality, age-specific mortality, and life expectancy, 1970-2016: a systematic analysis for the Global Burden of Disease Study 2016. *Lancet*. 2017;390(10100):1084-1150. doi:10.1016/S0140-6736(17)31833-0.

2. Golding N, Burstein R, Longbottom J, Browne AJ, Fullman N, Osgood-Zimmerman A, Earl L, Bhatt S, Cameron E, Casey DC, Dwyer-Lindgren L, Farag TH, Flaxman AD, Fraser MS, Gething PW, Gibson HS, Graetz N, Krause LK, Kulikoff XR, Lim SS, Mappin B, Morozoff C, Reiner RC, Sligar A, Smith DL, Wang H, Weiss DJ, Murray CJL, Moyes CL, Hay SI. Mapping under-5 and neonatal mortality in Africa, 2000-15: a baseline analysis for the Sustainable Development Goals. *Lancet*. 2017;390(10108):2171-2182. doi:10.1016/S0140- 6736(17)31758-0.

3. United Nations. Transforming our World: The 2030 Agenda for Sustainable Development .:. Sustainable Development Knowledge Platform. https://sustainabledevelopment.un.org/post2015/transformingourworld/publication. Accessed October 18, 2019.

4. Keenan JD, Bailey RL, West SK, Arzika AM, Hart J, Weaver J, Kalua K, Mrango Z, Ray KJ, Cook C, Lebas E, O'Brien KS, Emerson PM, Porco TC, Lietman TM. Azithromycin to Reduce Childhood Mortality in Sub-Saharan Africa. *N Engl J Med.*2018;378(17):1583-1592. doi:10.1056/NEJMoa1715474.

5. Doan T, Arzika AM, Hinterwirth A, Maliki R, Zhong L, Cummings S, Sarkar S, Chen C, Porco TC, Keenan JD, Lietman TM. Macrolide Resistance in MORDOR I — A Cluster- Randomized Trial in Niger. *N Engl J Med*. 2019;380(23):2271-2273. doi:10.1056/NEJMc1901535.

6. Keenan JD, Arzika AM, Maliki R, Boubacar N, Elh Adamou S, Moussa Ali M, Cook C, Lebas E, Lin Y, Ray KJ, O'Brien KS, Doan T, Oldenburg CE, Callahan EK, Emerson PM, Porco TC, Lietman TM. Longer-Term Assessment of Azithromycin for Reducing Childhood Mortality in Africa. *N Engl J Med*. 2019;380(23):2207-2214. doi:10.1056/NEJMoa1817213.

7. WHO, 2020. Guideline on Mass Drug Administration of Azithromycin to Children under Five Years of Age to Promote Child Survival. Available at: https://www.who.int/publications/i/item/9789240009585. Accessed October 9, 2020.

8. Oldenburg CE, Arzika AM, Maliki R, Lin Y, O'Brien KS, Keenan JD, et al. Optimizing the Number of Child Deaths Averted with Mass Azithromycin Distribution. *Am J Trop Med Hyg*. February 2020:1-3. doi:10.4269/ajtmh.19-0328.

9. Rose G. Sick individuals and sick populations. *International Journal of Epidemiology*. 2001;30(3):427-432. doi:10.1093/ije/30.3.427.

10. Yusuf S, Collins R, Peto R. Why do we need some large, simple randomized trials? *Stat Med*. 1984;3(4):409-422. doi:10.1002/sim.4780030421.

11. Hayes RJ, Moulton LH. *Cluster Randomized Trials.* Boca Raton: CRC/Taylor and Francis; 2009.

12. Burstein R, Henry NJ, Collison ML, Marczak LB, Sligar A, Watson S, Marquez N, Abbasalizad-

Farhangi M, Abbasi M, Abd-Allah F, Abdoli A, Abdollahi M, Abdollahpour I, Abdulkader RS, Abrigo MRM, Acharya D, Adebayo OM, Adekanmbi V, Adham D, Afshari M, Aghaali M, Ahmadi K, Ahmadi M, Ahmadpour E, Ahmed R, Akal CG, Akinyemi JO, Alahdab F, Alam N, Alamene GM, Alene KA, Alijanzadeh M, Alinia C, Alipour V, Aljunid SM, Almalki MJ, Al-Mekhlafi HM, Altirkawi K, Alvis-Guzman N, Amegah AK, Amini S, Amit AML, Anbari Z, Androudi S, Anjomshoa M, Ansari F, Antonio CAT, Arabloo J, Arefi Z, Aremu O, Armoon B, Arora A, Artaman A, Asadi A, Asadi-Aliabadi M, Ashraf-Ganjouei A, Assadi R, Ataeinia B, Atre SR, Quintanilla BPA, Ayanore MA, Azari S, Babaee E, Babazadeh A, Badawi A, Bagheri S, Bagherzadeh M, Baheiraei N, Balouchi A, Barac A, Bassat Q, Baune BT, Bayati M, Bedi N, Beghi E, Behzadifar M, Behzadifar M, Belay YB, Bell B, Bell ML, Berbada DA, Bernstein RS, Bhattacharjee NV, Bhattarai S, Bhutta ZA, Bijani A, Bohlouli S, Breitborde NJK, Britton G, Browne AJ, Nagaraja SB, Busse R, Butt ZA, Car J, Cárdenas R, Castañeda-Orjuela CA, Cerin E, Chanie WF, et al. Mapping 123 million neonatal, infant and child deaths between 2000 and 2017. *Nature*. 2019;574(7778):353-358. doi:10.1038/s41586-019-1545-0

13. Proctor E, Hiie Silmere, Raghavan R, Hovmand P, Aarons G, Bunger A, Griffey R, Hensley M. Outcomes for implementation research: conceptual distinctions, measurement challenges, and research agenda. *Administration and Policy in Mental Health*. 2011;38(2):65-76. doi:10.1007/s10477-010-0319-7

Final Statistical Analysis Plan

L'azithromycine pour la vie des enfants au Niger: implémentation et recherche (AVENIR)

An adaptive cluster-randomized trial to determine the optimal age group for implementation of biannual oral azithromycin distribution to reduce child mortality in Niger

Statistical Analysis Plan — Mortality and Resistance Trial

1. Administrative Information

**Trial registration:**	NCT04224987
**Funder:**	Bill & Melinda Gates Foundation (grant OPP1210548)
**SAP Version:**	Version 17, Updated 2025-05-27
	A revision history for this document is included at the end.
**Protocol Version:**	Refers to Protocol Version 30
**Contributors:**	Persons contributing to the SAP:
	Zijun Liu [1], Victoria Le [1], William Nguyen [1], Brittany Peterson [1]
	Statisticians responsible:
	Travis C. Porco [1], Benjamin F. Arnold [1]
	Principal Investigators:
	Thomas M. Lietman [1], Kieran O'Brien [1], Abdou Amza [2,3], Ahmed Arzika[4]

[1] Francis I. Proctor Foundation for Research in Ophthalmology, University of California, San Francisco, USA
[2] Ministère de la Santé Publique du Niger
[3] Le Programme National de Santé Oculaire (PNSO)
[4] Centre de recherche et interventions en santé publique (CRISP)

SAP Table of Contents

1. ADMINISTRATIVE INFORMATION ... 1
[4] CENTRE DE RECHERCHE ET INTERVENTIONS EN SANTÉ PUBLIQUE (CRISP) ... 1
2. OVERVIEW ... 3
Phase I Summary of the design ... 3
Trial masking ... 4
3. MORTALITY TRIAL ... 4
3.1. INTRODUCTION ... 4
3.1.1. Background and rationale ... 4
3.1.2. Objectives ... 4
3.2. STUDY METHODS ... 4
3.2.1. Trial design ... 4
3.2.2. Randomization ... 5
3.2.3. Sample size ... 6
3.2.4. Statistical framework ... 9
3.2.5. Statistical interim analyses and stopping guidance ... 9
3.2.6. Timing of the primary analysis ... 9
3.2.7. Timing of outcome assessments ... 9
3.3. STATISTICAL PRINCIPLES ... 10
3.3.1. Confidence intervals and P-values ... 10
3.3.2. Protocol deviations ... 10
3.3.3. Analysis Populations ... 11
3.4. TRIAL POPULATION ... 11
3.4.1. Screening data ... 11
3.4.2. Eligibility ... 11
3.4.3. Recruitment ... 12
3.4.4. Withdrawal/follow-up ... 12
3.4.5. Baseline patient characteristics ... 12
3.5. ANALYSIS ... 12
3.5.1. Outcome definitions ... 12
3.5.2. Analysis methods ... 17
3.5.3. Missing data ... 19
3.5.4. Additional analyses ... 19
Model Assessment ... 19
Subgroup Analyses ... 19
Bayesian Analysis ... 20
3.5.5. Harms ... 21
3.5.6. Statistical software ... 21
4. REFERENCES ... 21
5. REVISION HISTORY ... 22

The content of this Statistical Analysis Plan meets the requirements stated by the US Food and Drug Administration and conforms to the American Statistical Association's Ethical Guidelines.This SAP was organized following guidelines proposed by Gamble et al. [1]

The companion computational notebooks with underlying sample size calculations presented herein is entitled: `(1) AVENIR_Power_Calculation_v8.Rmd / .html`, `(2) AVENIR-addendum-AMR-`

`direct-power-calc_052025.Rmd / .pdf`, (3) `AVENIR-addendum-spillover-power-calc_stool_NP_052325.Rmd / .html`; they are saved in the same directory as this document.

2. Overview

The AVENIR study includes multiple different trials, each with different objectives (Figure 1). This Statistical Analysis Plan (SAP) includes details for the Mortality and Resistance Trial. Separate SAPs cover the Delivery and Programmatic trials.

Phase I Summary of the design

Figure 1. Phase I summary of design.

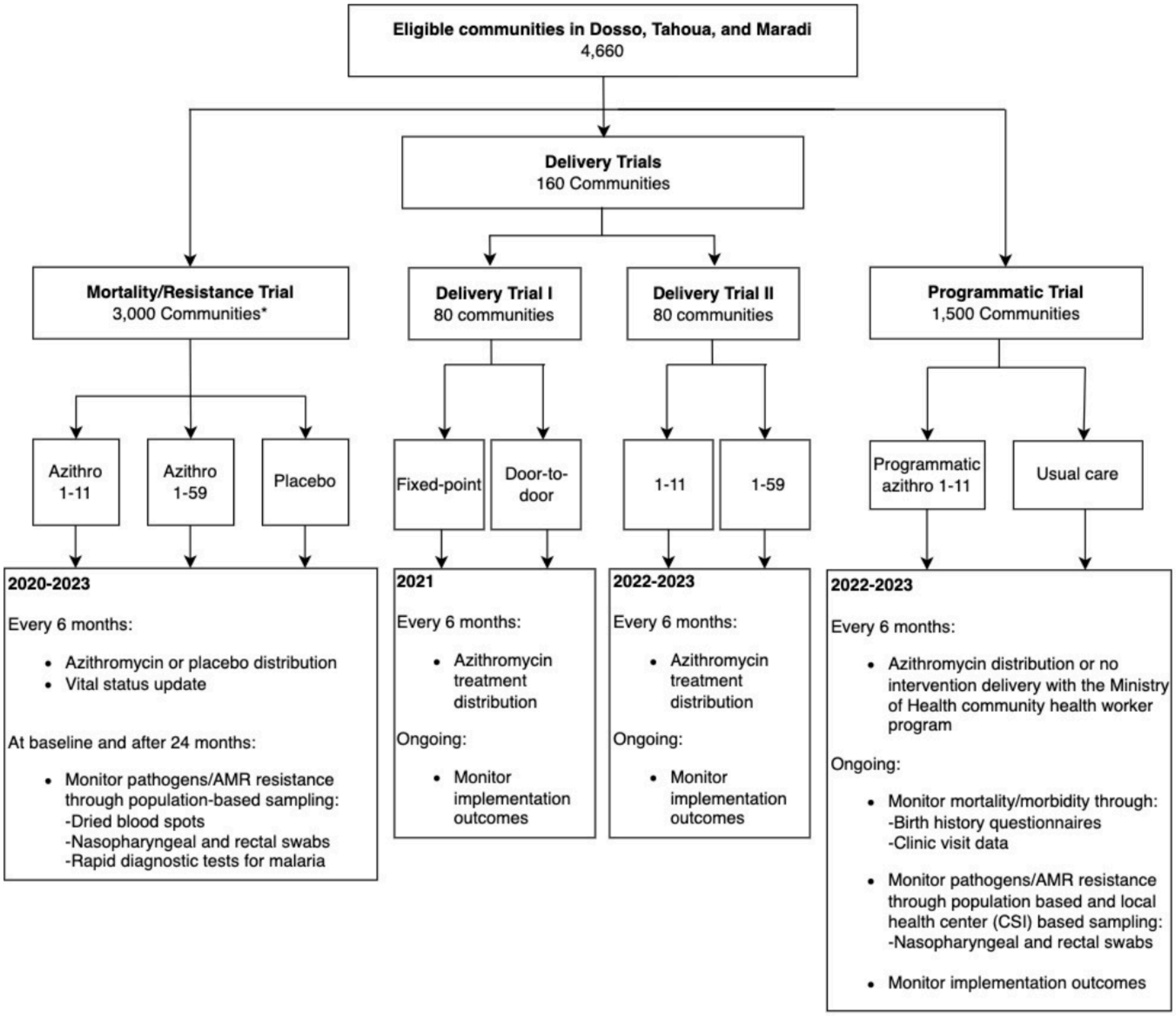

*A random sample of 150 communities was selected for AMR monitoring, 60 of which contribute to the primary

Trial masking

The Mortality/Resistance trial is a double-masked, placebo controlled trial. All participants are masked to their treatement. Study investigators and field staff are masked to treatment with the exception of the unmasked portion of the trial analysis team at UCSF. The unmasked portion of the analysis team will conduct the response adaptive allocation and pre-specified interim analyses. Unmasked members include one of the four PIs for the trial (Thomas Lietman), one of the two biostatisticians for the trial (Travis Porco), and one analyst (Zijun Liu). These members were designated in January 2022 before any adaptation or interim analyses would commence. The rationale for including this composition of the unmasked analysis team was to ensure sufficient statistical and subject matter expertise to guide the critical interim discussions and decisions for the response-adaptive trial, in coordination with the trial's Data and Safety Monitoring Committee.

The Delivery Trials and Programmatic Trial are unmasked and do not include a placebo.

3. Mortality Trial

3.1. Introduction

3.1.1. Background and rationale

This document (Statistical Analysis Plan, SAP) describes the planned analysis and reporting for the clinical trial, L'azithromycine pour la vie des enfants au Niger: implémentation et recherche (AVENIR). It includes specifications for the statistical analyses and tables to be prepared for the final Clinical Study Report. This study is a Phase IV clinical trial to compare methods to reduce childhood mortality using mass administration of azithromycin (Pfizer, CAS 83905-01-5) compared to placebo.

3.1.2. Objectives

AVENIR is designed to assess the efficacy of mass distribution of azithromycin to prevent mortality among children.

The overall objectives of this study are to:

1. Determine the optimal age group to treat with biannual oral azithromycin distribution to reduce child mortality
2. To compare selection for antimicrobial resistance across different age-based strategies for biannual azithromycin distribution
3. Demonstrate large-scale implementation of this program.

3.2. Study Methods

3.2.1. Trial design

AVENIR is designed as a large simple double-masked cluster-randomized trial with response-adaptive allocation (a platform, perpetual trial). The rationale for adaptive

randomization is to allow all communities to have the opportunity to be treated, and to allow the program to evolve into the optimal treatment. If new treatment arms are added to AVENIR in the future, or if any of the current treatment arms are discontinued, the Protocol and SAP will reflect those updates.

Eligible communities in Niger will be randomly selected to participate in a mortality/resistance trial, a delivery trial or a delayed intervention.

In the mortality/resistance trial, communities will be randomized to one of three azithromycin distribution strategies: 1) **azithro 1-11:** biannual oral azithromycin to children 1-11 months old with biannual oral placebo to children 12-59 months old, 2) **azithro 1-59:** biannual oral azithromycin to children 1-59 months old, or 3) **placebo:** biannual oral placebo to children 1-59 months old. Mortality will be monitored through biannual census data collection, which will be used to adaptively allocation treatment assignments after the first year. Communities will keep their group assignment for 4 distributions. Antimicrobial resistance will be monitored in a random subsample of treated and untreated children and adults in the Dosso region. See Figure 1 for a summary of the deisgn.

Details are provided in the Protocol document, INV_002454_2019.

Mortality/Resistance Trial Interventions

Communities will be randomized to the following 3 arms, with the allocation updated after a run-in period of 1 year according to the effect of each on the mortality rate among children aged 1-59 months as determined by inter-census intervals:

- Azithromycin 1-11: biannual weight- or height-based dose of oral azithromycin suspension to children 1-11 months old and oral placebo to children 12-59 months old
- Azithromycin 1-59: biannual weight- or height-based dose of oral azithromycin suspension to children 1-59 months old
- Placebo: biannual weight- or height-based dose of oral placebo to children 1-59 months old

3.2.2. Randomization

AVENIR includes two cluster randomized trials (mortality/resistance and delivery), with grappes as the unit of randomization. Throughout we will refer to grappes as “communities”.

Mortality/Resistance Trial

At the start of the trial, communities will be allocated equally across the three arms, 1:1:1. After a run-in period of 1 year, the trial will update allocation probabilities every 6 months, corresponding to each census. Allocation probabilities between the arms will adapt based on the probability of that arm being the superior arm with respect to the mortality rate among children 1-59 months, so that newly enrolled communities will have a higher probability of being allocated to the arm with the lowest mortality rate. To minimize the impact of a possible carry-over effect of treatment in the event that a community changes arms through adaptive allocation, each community will stay with its randomized assignment for 4 distributions (approximately 2 years). Note that no community will be re-randomized by the time of the primary outcome.

Response Adaptive Allocation Algorithm

We will use the following algorithm for the adaptive allocation. Given all outcome measurements available at a given time, we will fit a negative binomial model to the data with indicators for each arm (see section 6.2 for analysis details). From the model, we will estimate each arm's log mortality rate and its standard error. We will then draw 10,000 replicates of log mortality rates from the estimated distributions, assuming they are normally distributed, and in each draw of the joint distribution we will determine which arm has the lowest mortality rate. We will estimate the probability that each arm has the lowest mortality rate as the proportion of 10,000 replicates in which the arm has the lowest mortality rate. Following calculation of the probability that each arm has lowest mortality, we will then use a root transform of the probabilities to yield the allocation probabilities. Specifically, if p_i is the probability that arm i ($i = 1{,}2{,}3$) yields the best outcomes, then compute the allocation probabilities $a_i = p_i^{\gamma} / \sum_{j=1}^{3} p_j^{\gamma}$. We pre-specified a tempering value known only to the unmasked team to ensure masking: γ = 1/4 or 1/6 or 1/8. This transformation will help mitigate violent swings in allocation due to chance alone. Finally, we will not allow any arm's allocation probability to fall below 10% to preserve our ability to compare it against alternatives through the primary endpoint.

3.2.3. Sample size

We anticipate conducting the study in five regions of Niger: Dosso, Tahoua, Maradi, Zinder, Tillabéri; full details are given in the Protocol.

For the first two years, we expect approximately 1,400 communities in Dosso to participate for two full years (4 inter-census intervals of six month duration) and approximately 975 communities from Tahoua to participate for one year (2 inter-census intervals of six month duration) and 975 more communities from Tahoua to participate for half a year (1 inter-census interval). The sample size calculations below were informed by mortality rates and standard deviations measured in the MORDOR Niger trial [2].

Mortality rate measured as deaths per 1,000 person-years at risk will be the primary mortality outcome. The use of mortality rate protects against delays in start time across communities due to security or logistical issues, as person-time at risk naturally accounts for different follow-up times. Delayed implementation or exclusion of additional communities for security concerns could result in fewer inter-census intervals. *The sample size calculation assumes 8,525 inter-census intervals and 1,116 communities per arm.*

Mortality primary outcomes

Mortality primary outcome 1: mortality in 1-59 month olds, assessed at 2.5 years (based on at most 2 years of follow-up per community), comparing the 1-59 month and the placebo arms. Assuming a standard deviation of approximately 0.023, we find that 1,116 communities per arm will provide approximately 80% power to detect a 10% relative reduction, assuming a baseline mortality rate of 27 per thousand person-years among children 1-59 months old and an alpha of 0.05. The absolute effect size is a mortality rate difference of 2.7 deaths per 1000 person-years. This calculation is based on the standard Z-test formula for power (treating the mortality rate in each community as a continuous variable). The calculation does not involve a correction for multiple comparisons since the analysis uses a hierarchical testing approach for the three mortality outcomes, as described below.

Mortality primary outcome 2: mortality in 1-11 month olds, assessed at 2.5 years (based on at most 2 years of follow-up per community), comparing the 1-11 month arm and the placebo arm. Assuming a standard deviation of approximately 0.074, we find that 1,116 communities per arm will provide approximately 80% power to detect a 19% relative reduction, assuming a baseline mortality rate of 45 deaths per thousand person-years among children 1-11 months old and an alpha of 0.05. The absolute effect size is a mortality rate difference of 8.8 per 1000 person-years. This calculation is based on the standard Z-test formula for power (treating the mortality rate in each community as a continuous variable). The calculation does not involve a correction for multiple comparisons since the analysis uses a hierarchical testing approach for the three mortality outcomes, as described below.

Mortality primary outcome 3: mortality in 12-59 month olds, assessed at 2.5 years (based on at most 2 years of follow-up per community), comparing the 1-11 month and 1-59 treatment arms. Assuming a standard deviation of approximately 0.022, we find that 1,116 communities per arm will provide approximately 80% power to detect a 11% relative reduction, assuming a baseline mortality rate of 24 deaths per thousand person-years among children 12-59 months old and an alpha of 0.05. This corresponds to an absolute reduction of 2.7 in the death rate per 1000 person-years. This calculation is based on the standard Z-test formula for power (treating the mortality rate in each community as a continuous variable).

Sensitivity analyses for primary outcomes based on reduced enrollment caused by security or logistical concerns: if only the first half of Tahoua communities are enrolled by 2.5 years, with 791 communities per arm, we anticipate the following detectable relative detectable effect sizes (respectively): 12%, 23% and 13%, for 1-59 months, 1-11 months and 12-59 months.

Mortality secondary outcomes

Secondary outcome 1 : mortality rate among 12-59 months comparing the 1-11 and Placebo arms. The calculation is similar to *Primary outcome 3*. So assuming a standard deviation of approximately 0.022, we find that 1,116 communities per arm will provide approximately 80% power to detect a 11% relative reduction, assuming a baseline mortality rate of 24 per thousand person-years and an alpha of 0.05. This corresponds to an absolute reduction of 2.7 in the death rate per 1000 person-years.

Secondary outcome 2: mortality rate among 1-11 months comparing the 1-11 month and 1-59 month arms. The calculation is similar to *Primary outcome 2*. So assuming a standard deviation of approximately 0.074, we find that 1,116 communities per arm will provide approximately 80% power to detect a 19% relative reduction, assuming a baseline mortality rate of 45 per thousand person-years and an alpha of 0.05. This corresponds to an absolute reduction of 8.8 in the death rate per 1000 person-years.

Sensitivity analyses for secondary outcomes based on reduced enrollment caused by security or logistical concerns: With 791 communities per arm, we anticipate the following relative detectable effect sizes (respectively): 13% and 23%, for 12-59 month olds and 1-11 month olds.

Antimicrobial Resistance (AMR) Monitoring Outcomes

AMR primary outcome 1: Load of genetic determinants of resistance to macrolides including those determinants known to be found in *Streptococcus pneumoniae*, *Streptococcus pyogenes*,

and *Staphylococcus aureus* from nasopharyngeal swabs in children 1-59 months old at 24 months from baseline. We anticipate inclusion of 50 communities per arm (150 total) provides approximately 80% power to detect an approximately 2-fold difference in bacterial reads that are resistant in all age groups between any two arms, assuming:

- Swabs are pooled at the community level
- SD of resistance load (on log scale) equal to 1.88 (CHAT)
- Alpha of 0.05 (two-tailed)

AMR primary outcome 2: Load of genetic determinants of resistance to macrolides including those determinants known to be found in *Campylobacter, Salmonella* spp.*, Shigella* spp., and *Escherichia coli* from rectal swabs in children 1-59 months old at 24 months from baseline. We anticipate inclusion of 50 communities per arm (150 total) provides approximately 80% power to detect a 2.6-fold difference in bacterial reads that are resistant all age groups between any two arms, assuming:

- Swabs are pooled at the community level
- SD of resistance load (on log scale) equal to 2.43 (MORDOR I)
- Alpha of 0.05 (two-tailed)

AMR secondary outcome 1 (as an example of detectable effects for other AMR secondary outcomes): For proportion of pneumococcal isolates from nasopharyngeal swabs in children 1-59 months resistant to macrolides on phenotypic testing, we anticipate inclusion of 50 communities per arm (150 total) provides approximately 80% power to detect a 8% absolute increase between any two arms, assuming:

- Baseline resistance of 10% (from MORDOR I)
- ICC of 0.045 (from multiple studies)
- 10 specimens from each of 20 communities from each arm (8 after accounting for specimen nongrowth)
- Alpha of 0.05 (two-tailed)

AMR spillover outcome 1: For the comparison of a summary measure in the mean load of genetic determinants in caregiver/guardian stool samples, we estimated that 20 communities per arm have 80% power to detect a 1.25-fold difference, assuming:

- Swabs are pooled at the community level
- SD of resistance load (on log scale) equal to 0.35 (approximated using the normalized macrolide reads in the placebo arm of the rectal sample collected in this study)
- Alpha of 0.05 (two-tailed)

AMR spillover outcome 2: For the summary measure of the mean load of genetic determinants in NP samples (collected from caregiver/guardians and, separately, children 7-12 years old), we estimated that 50 communities per arm have 80% power to detect a 2.09-fold difference, assuming:

- Swabs are pooled at the community level
- SD of resistance load (on log scale) equal to 1.88 (from the CHAT trial)
- Alpha of 0.05 (two-tailed)

3.2.4. Statistical framework

The overall analysis is divided into two aspects: 1) A single frequentist analysis, conducted at the time 2.5 years after enrollment, will test the hypothesis that azithromycin MDA reduces childhood mortality. The hypothesis tests will be superiority tests (as opposed to equivalence or noninferiority analyses) and will be two-sided. 2) Ongoing analyses beyond the primary endpoint, reflecting the perpetual nature of the trial, will be conducted using a Bayesian perspective.

3.2.5. Statistical interim analyses and stopping guidance

The adaptive design will naturally change allocation, evolving toward higher allocation probabilities for arms most likely to have lowest mortality. From a frequentist perspective, we propose a interim analysis at 18 months after the first enrollment (12 months of data in each community). The analysis will be based on the Haybittle-Peto boundary which results in an interim alpha of 0.001. The alpha of the final analysis will continue to be 0.05 according to the rule.[3] The Data and Safety Monitoring Committee will be notified of the results, and if the p-value (hierarchical approach, same as primary outcome) is <0.001, then the DSMC and if appropriate, one or more PIs will meet to decide a course of action. Factors in any decision would include interest in both the 1-59 month and 1-11 month MDAs, and AMR. Early discontinuation for other reasons (political, ethical, lack of resources, etc) would mandate final frequentist analysis at alpha of the full final prespecified value based on all available data (incorporating any alpha spending, as needed). We note that the DSMC may advise discontinuation based on evidence of harm, even in the absence of statistical significance. Bayesian inferences are unchanged by the application of interim analyses. Continuous Bayesian monitoring will be conducted.

Interim reporting

Reports will be planned for the Data and Safety Monitoring Committee. We will report the number of treatments delivered, the number of communities enrolled at each phase, and the coverage by community (i.e., process indicators). Overall mortality rates by age group (1-11, 12-59, and 1-59) will be tabulated overall in open reports.

We note that response adaptive randomization for the mortality trial will begin for communities enrolled at and after the one year mark.

3.2.6. Timing of the primary analysis

Mortality. The primary analysis will occur 2 years (rounded to the day) after the completion of enrollment. The analysis will use all the data available at that time; i.e., we do not propose to wait until phase completion for individuals enrolled and being currently followed at the 2-year mark, but rather, to use all complete data records at that moment.

AMR. Analysis of resistance outcomes will occur after the 24-month community-based samples are analyzed in the lab.

3.2.7. Timing of outcome assessments

Mortaltiy outcomes are measured at the end of each prespecified inter-census interval, intended to be six months in duration. The primary analysis will include outcomes assessed up

to 2.5 years after the first enrollment. AMR outcomes are assessed at baseline and after 24 months of intervention.

3.3. Statistical Principles

3.3.1. Confidence intervals and *P*-values

The primary analysis for the mortality trial is prespecified to occur at alpha of 0.05, as follows. We propose to use a fixed-sequence, hierarchical testing approach for the three mortality primary outcomes [4]:

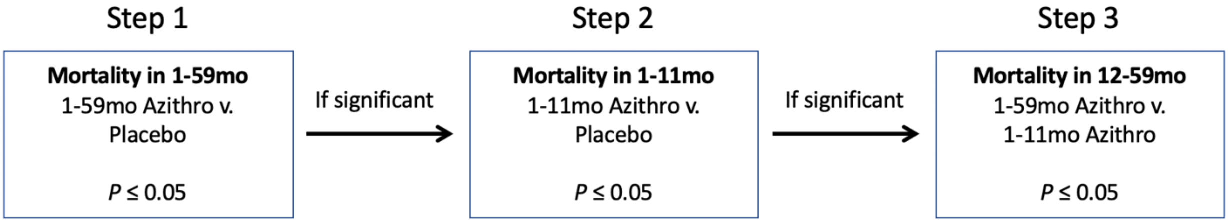

The staged testing approach will maximize the trial's power. Our rationale for a fixed-sequence testing approach is if there is no benefit of treating all children 1-59 mo compared with placebo, then the trial would be unlikely to demonstrate benefit between treatment in the narrower age subgroups. Note that the hypothesis testing in the mortality trial and implementation trial will proceed independently for primary outcomes along the same sequence of tests. Regardless of the hypothesis testing results, we will report 95% confidence intervals around estimates of the incidence rate ratio for the comparisons of the trial arms.

In the mortality trial AMR substudy, we propose a second set of tests. There are two primary outcomes measured among children 1-59 months: load of genetic determinants of macrolide resistance measured in NP swabs, and load of genetic determinants of macrolide resistance measured in rectal swabs. Each primary outcome in the substudy will be examined separately at alpha of 0.05. For each outcome, we propose three pair-wise comparisons between the study groups (Placebo v 1-59mo, Placebo v 1-11mo, 1-59mo v 1-11mo). Additional secondary comparisons between groups in the substudy will include proportion of pneumococcal isolates resistant to macrolides on phenotypic testing (NP swabs) and load of genetic determinants to non-macrolide antibiotics (NP and rectal swabs). Within each outcome, we will report *P*-values adjusted to control the false discovery rate at 5% the Benjamini-Hochberg approach [5]. For all AMR endpoints, we will additionally report unadjusted *P*-values, which will be less conservative to detect undesirable outcomes resulting from mass distribution of azithromycin.

3.3.2. Protocol deviations

The fraction of eligible censused children who accept the intervention will be reported. Communities for which this fraction falls below 60% at a specific census will be tabulated and reported.

In addition, if the fraction of children identified on a subsequent census falls below 75%, an investigation will be conducted. The number of such communities will be tabulated and reported, and sensitivity analyses omitting such communities will be reported.

Formal protocol deviations of two types are noted: (1) individual-level deviation, in which an ineligible individual is mistakenly treated, and (2) community-level deviation, in which a community is mistakenly treated with the wrong intervention. Any such events will be tabulated

and reported. Note that any community which receives the wrong treatment will be analyzed according to the original randomization assignment (intention to treat).

3.3.3. Analysis Populations

Community-level data will be analyzed on an intention-to-treat basis, covering all inter-census intervals for which follow-up data are available. The random sample of communities selected for the AMR substudy will contribute to the mortality analyses.

Since AVENIR will randomize grappes before formal enrollment at baseline, the trial will analyze only grappes that are enrolled at baseline according to their assigned treatment. Grappes that are randomized but do not meet the trial's enrollment criteria will not be included in the analysis.

Modified per protocol analyses will be conducted that exclude communities with below 60% antibiotic coverage in the targeted age group. The number of such communities and their mean coverage will be reported, and such analyses will be sharply distinguished from the prespecified primary and secondary analyses.

3.4. Trial Population

3.4.1. Screening data

AVENIR will report the number of communities screened by region and the reasons communities were not enrolled based on eligibility criteria described in the next section.

3.4.2. Eligibility

This information repeats Protocol v10 section 4.3 Eligibility - Intervention.

At the community-level, eligibility includes:

Inclusion Criteria:
- Location in Dosso, Tahoua, Maradi, Zinder, or Tillabéri regions
- Population 250 to 2,499*
- Distance > 5 km from district headquarters town
- Verbal consent of community leader(s)

Exclusion criteria:
- Inaccessible or unsafe for study team
- "Quartier" designation on national census

*Population size as estimated from the most recent national census or projections

At the individual-level, eligibility includes:

Inclusion criteria:
- Age 1-59 months
- Primary residence in a study community
- Verbal consent of caregiver/guardian for study participation

- Weight ≥ 3.0 kg

Exclusion criteria:

- Known allergy to macrolides

3.4.3. Recruitment

The CONSORT flow diagram will follow the model of the MORDOR I trial [2] and will include a tabulation of the number of (a) available communities, (b) eligible communities, and (c) enrolled communities by arm.

3.4.4. Withdrawal/follow-up

Additional information will be provided. Specifically, (d) average number of individuals per community, (e) coverage and number of treatments given, and (f) number of individuals for whom end of intercensus mortality data are available.

As a large simple trial, AVENIR will not collect information regarding the specific reasons or timing for any loss to follow-up at the individual level.

3.4.5. Baseline patient characteristics

Since AVENIR is a large simple trial it will not collect extensive information about individual participants. We propose to summarize the community size (number of eligible children), child age distribution, and percentage female for enrolled communities.

3.5. Analysis

3.5.1. Outcome definitions

Mortality Trial Primary Outcomes

The trial has three primary outcomes, each a different comparison of the mortality rate (deaths per 1,000 persons at risk) at 2.5 years from the first child enrolled, with a window of 3 months.

- Mortality rate (deaths per 1,000 person-years at risk) among children 1-59 months of age compared between communities that receive azithromycin delivered to children ages 1-59 months and communities that receive placebo at 2.5 years
- Mortality rate (deaths per 1,000 person-years at risk) among children 1-11 months of age compared between communities that receive azithromycin delivered only to children ages 1-11 months and communities that receive placebo at 2.5 years
- Mortality rate (deaths per 1,000 person-years at risk) among children 12-59 months of age compared between communities that receive azithromycin delivered to children ages 1-11 months and communities that receive azithromycin delivered to children ages 1-59 months at 2.5 years

Mortality will be monitored through biannual census data collection. Trained census workers will use a custom-designed mobile application on smartphones to conduct the census. Census workers will visit each household in assigned study communities to obtain household consent

for participation, and to collect demographic data on the head of household, all mothers in the household, and all children 1-59 months in the household. For eligible children, dose will be determined via weight or height and administered by the census worker. At subsequent census periods, the census workers return to each household and update the vital status of all children, indicating whether a child is alive, has died, has moved, or has an unknown status. New children and households will be added.

A child will be included as a death in the primary outcome analysis if they are present on one census and absent on the subsequent census due to death (inter-census interval). Person-time at risk will be calculated as time alive and eligible for treatment while living in the study area, with children who died contributing half of the person-time for their last inter-census interval. Children who moved, have an unknown status, or are missing from one census will contribute no person-time.

If the study team fails to measure a community for a single census due to security or logistical reasons, but re-visits the community in the following census, then person-time and outcomes for the 1-year period (2 inter-census intervals) will be counted toward the primary outcome. In this case, as with the 6-month inter-census interval, person-time at risk will be calculated as time alive and eligible for treatment for the double inter-census interval (approximately 12 months), and children who moved, have an unknown status, or are missing from one census contributing no person-time. Children who died will contribute half of the person-time between the first census date of the double inter-census interval and their death date. Death date will be used to determine to which inter-census interval the death will belong.

Mortality Trial Secondary Outcomes

Two secondary outcomes include mortality rate comparisons at 2.5 years (±3 months) from the first child enrolled for the remaining comparisons between different age groups and arms

- Mortality rate (deaths per 1,000 person-years at risk) among children ages 12-59 months, with rates compared between communities that receive azithromycin delivered only to children ages 1-11 months and communities that receive placebo
- Mortality rate (deaths per 1,000 person-years at risk) among children ages 1-11 months, with rates compared between communities that receive azithromycin delivered only to children ages 1-11 months and communities that receive azithromycin delivered to children ages 1-59 months

Antimicrobial Resistance Substudy Primary Outcomes

The trial will have two primary outcomes for antimicrobial resistance (AMR):

- Load of genetic determinants of resistance to macrolides including those determinants known to be found in *Streptococcus pneumoniae*, *Streptococcus pyogenes*, and *Staphylococcus aureus* from nasopharyngeal swabs in children 1-59 months old at 24 months from baseline
- Load of genetic determinants of resistance to macrolides including those determinants known to be found in *Campylobacter*, *Salmonella* spp., *Shigella* spp., and *Escherichia coli* from rectal swabs in children 1-59 months old after 2 years of distributions

Antimicrobial Resistance Substudy Secondary Outcomes

- Load of genetic determinants of resistance to other antibiotic classes in all swabs and samples listed above
- Proportion of macrolide resistant pneumococcal isolates from nasopharyngeal swabs collected from children 1-59 months

Antimicrobial Resistance Age-group Spillover Outcomes

- Load of genetic determinants of resistance to macrolides and other antibiotic classes from stool samples in caregivers/guardians of eligible children after 2 years of distributions (post-test only)
- Load of genetic determinants of resistance to macrolides from nasopharyngeal swabs collected from children 7-12 years old after 2 years of distributions
- Load of genetic determinants of resistance to macrolides from nasopharyngeal swabs collected from caregivers/guardians of eligible children after 2 years of distributions

Microbiome Substudy Outcomes.

Nasopharyngeal samples will be collected from children 1-59 months of age, children 7-12 years of age, and caregivers/guardians of children 1-59 months of age as described above and in the protocol. Rectal swabs will be collected from children 1-59 months of age as described above and in the protocol. Each age group and sample type will be used to assess the following outcomes:

- Overall microbial structure after 2 years of distribution using Manhattan distance, adjusted for baseline and analyzed with PERMANOVA to compare by arm. Euclidean distance will be used in a secondary analysis.
- Shannon's diversity index (expressed as effective number) of the microbiome after 2 years of distribution, analyzed in a linear model with a covariate for arm. Inverse Simpson's index (expressed as effective number) will be used in a secondary analysis.
- Relative abundance of individual microbial taxa at the genus and species level after 2 years of distribution and analyzed with DESeq2 to compare by arm with a Benjamini-Hochberg correction to account for the number of comparisons using a false discovery rate ranging from 5% to 10%.

Additional Secondary Outcomes

- Mortality rate over 2.5 years by subgroups:
  - Region (Dosso, Tahoua, Maradi, Zinder, and Tillabéri)
  - Child age (1-11m, 12-24m, 24-59m)
  - Child sex
  - Community distance to the nearest Centre de Santé Intégré (CSI)
  - Number of previous community distributions
  - Timing of distribution/seasonality
  - Other interventions
    - Participation in MORDOR trials
    - Participation in and coverage of Seasonal Malaria Chemoprevention (SMC)
    - Participation in trachoma distributions of azithromycin

- Nutritional status as defined by weight, MUAC, height interval

- Program costs as captured by routine administrative data collection during the study period and by micro-costing activities (T&M)
  - *Measured cost per dose* of azithromycin MDA in Niger for the two age groups (1 – 11 months of age and 1 – 59 months of age)
  - *Variation in measured cost per dose* of azithromycin MDA as a function of community characteristics.
  - *Modeled cost per dose* of an azithromycin MDA in new but similar settings. Values will be assigned to each *input cost category* (personnel, consumables, equipment, services, training, space), *activity* (eg, publicity, transport), and level (communities / household / child) using a fixed and variable cost perspective adjusted to the level, and adjusted for community-level characteristics.
  - Total observed azithromycin MDA program costs, by age group (1 – 11 month versus 1 – 59 month)

- Cost-effectiveness measured by estimated program costs and using measured mortality and estimated DALYs as the effectiveness outcome, compared across arms.
  - ICER for administering azithromycin to the different age groups, comparing the cost and effectiveness of 1 – 59 month vs. 1-11 month.

- Estimated 10 year national-scale effects (health and cost) of scaling up each strategy.

- Other implementation outcomes defined in the Proctor Outcomes Framework,[6][2] including feasibility and acceptability, as captured during routine administrative and monitoring data collection during the study period and by stakeholder interviews at the participant, community, and health system levels at 2.5 years (Table 3).

- Change in nutritional status over time as defined by weight, mid-upper arm circumference (MUAC), and height interval

- All-cause clinic visits among children 1-59 months of age in the study area during the study period as assessed through passive surveillance of Centre de Santé Integré records.

- Cause-specific clinic visits among children 1-59 months of age in the study area during the study period as assessed through passive surveillance of Centre de Santé Integré records, including visits with diagnoses of respiratory infection, diarrheal disease, and malaria.

- Respiratory microbiome by arm in all age groups (children 1-59 month old, children 7-12 year old and caregivers/guardians) as specified microbiome substudy primary outcomes

Other Outcomes

- Cause-specific mortality by treatment arm as defined by verbal autopsy* over 2.5 years

- Serology outcomes as assessed by dried blood spots during sample collections at baseline and 24 months
- Normalized read number of genetic determinants of resistance by antibiotic class in environmental samples at baseline and 24 months

*Depending upon resources, verbal autopsy might be conducted by UCSF or another group

MORDOR trial subgroup continuation outcomes

The AVENIR trial will include the 594 villages in Dosso region that were previously enrolled in the MORDOR I, II and III trials. This creates an opportunity to study the longer-term effects of mass azithromycin treatment on and on antimicrobial resistance.

- Longer-term carry-over effects on mortality
    - MORDOR III, beginning at month 42, re-randomized villages to receive bi-annual mass distribution of azithromycin or placebo to children 1-59 months, and followed the communities for 18 additional months (in three inter-census intervals 42-48m, 48-54m, 54-60m). From MORDOR communities allocated to the placebo arm of AVENIR, we will compare the mortality rate among children ages 1-59 months over 2.5 years in communities that received azithromycin during MORDOR III versus those that received placebo during MORDOR III. Our primary comparison of interest is among communities allocated to AVENIR's placebo arm because it will enable us to study longer-term carry-over effects of previous azithromycin treatment after discontinuation. We hypothesize that communities that previously received azithromycin during MORDOR phase 3 will have lower mortality compared to communities that continually received placebo for the entire period. The analysis approach will follow the primary analysis, estimating incidence rate ratios using a negative binomial regression. Comparisons within additional AVENIR arms (1-59m, 1-11m) could be estimated as an exploratory analysis.

- Longer-term carry-over effects on antimicrobial resistance
    - During MORDOR III, 160 communities were tested for genetic determinants of antimicrobial resistance at month 54. This creates the opportunity to study effects of treatment history on longer-term effects for antimicrobial resistance selection. From the 160 communities in the MORDOR trial with resistance measures, the AVENIR trial will randomly select 40 communities, evenly distributed (20/20) between two treatment histories (AAAAA, AAAPP, each letter refers to a year of Azithromycin or Placebo). Within each community, we will collect a rectal swab from 30 children 1-59 months, and test the samples for genetic determinants of antimicrobial resistance using metagenomic deep sequencing, as in the resistance primary analysis. This additional substudy will enable two analyses of primary interest:
        - At enrollment in AVENIR, approximately 1 year after the end of MORDOR, we will compare the load of genetic determinants of antimicrobial resistance to macrolides (primary) and other classes of antibiotics (secondarily) between the two treatment histories. We hypothesize that load of genetic determinants will be higher among communities with the AAAAA history compared with AAAPP history. The analysis approach will follow the primary analysis for the antimicrobial resistance substudy
        - Among the 20 communities with AAAPP treatment history in MORDOR, we will compare the the load of genetic determinants of antimicrobial resistance to macrolides (primary) and other classes of antibiotics (secondarily) longitudinally

over time from MORDOR 54 to AVENIR enrollment (approximately 1 year later), and again to the final measurement in AVENIR phase 1 (approximately 3 years after discontinuation of azithromycin). We hypothesize that load of genetic determinants will decline with time from last treatment.

3.5.2. Analysis methods

Mortality in the mortality/resistance trial

Our parameter of interest for comparisons of mortality between arms is the relative reduction in mortality measured by the incidence rate ratio (IRR). The primary analysis will be a Poisson regression that includes the count of deaths as the outcome and the community-level person-time at risk as an offset, with observations for each community's inter-census interval. The model will include an indicator variable for each active treatment arm. The adaptive allocation will be accounted for using pooled regression, including in the model additional indicator variables for each of three allocations in the trial (the initial, equal allocation plus 2 adaptations). For community *i* and randomization *j* (*j* = 1, 2, 3), the model will be:

$$\log(Y_{ij}) - \log(T_{ij}) = \alpha + \beta A_{ij} + \sum_{j=2}^{3} \gamma_j P_j,$$

where Y_{ij} is the number of deaths and T_{ij} is the person-time at risk in that community; A_{ij} is a treatment indicator that will vary by each comparison (details below), and P_j is an indicator for the two adaptive random allocations (the first equal allocation is the omitted category). We will estimate 95% confidence intervals for the IRRs using bootstrap resampling (10,000 replicates) that resamples communities with replacement and re-estimates the IRRs.

For primary outcome 1 that compares mortality among 1-59 month olds, we will include in the analysis children ages 1-59 months in the 1-59 month treatment arm and placebo arm. A_{ij} will be an indicator of allocation to the 1-59 month treatment arm, and $\exp(\hat{\beta})$ will estimate the IRR.

For primary outcome 2 that compares mortality among children ages 1-11 months, we will subset the analysis to children ages 1-11 months enrolled in the 1-11 month treatment arm and placebo arm. A_{ij} will be an indicator of allocation to the 1-11 month treatment arm, and $\exp(\hat{\beta})$ will estimate the IRR.

Similarly, for primary outcome 3 that compares mortality among children ages 12-59 months, we will subset the analysis to children ages 12-59 month in the 1-11 month treatment arm and 1-59 month treatment arm. A_{ij} will be an indicator of allocation to the 1-59 month treatment arm, and $\exp(\hat{\beta})$ will estimate the IRR.

Secondary mortality analyses will be estimated similarly from the model subset to the appropriate age range.

We will estimate permutation *P*-values for each comparison using a randomization test that permutes community treatment labels within each adaptive allocation group according to the probabilities used in each allocation, holding community outcomes fixed, and will assess statistical significance using the fixed sequence, hierarchical testing procedure described above in section 3.3.

We will examine effect heterogeneity by pre-specified effect modifiers on additive and multiplicative scales [8]. We will do so by including interaction terms between effect modifiers and treatment indicators in the regression models.

Antimicrobial Resistance Substudy

The primary analysis for nasopharyngeal swabs among pooled community-level samples will be a series of pairwise comparisons to compare the mean load of macrolide resistant genetic determinants at 24 months across the relevant arms (Placebo v. 1-59mo, Placebo v. 1-11mo, 1-59mo v. 1-11mo). The analysis will be hierarchical and follow a gatekeeping order to control the family-wise error rate to 5% in the following priority 1) Placebo v. 1-59 months Azithromycin, 2) Placebo v. 1-11 months Azithromycin and 3) 1-11 months Azithromycin v. 1-59 months Azithromycin.

For both nasopharyngeal and rectal swabs, prior to analysis, normalized reads were transformed using $log2(x + (\frac{1}{Non-Host\ Reads})$ to account for differences in sequencing depth across each community. *P*-values for all resistance analyses will be computed using the Wilcoxon Rank Sum test, with the T-test as a validation check, and using a Benjamini-Hochberg correction to control the false discovery rate (FDR) across additional (non-macrolide) endpoints, described above in section 3.3.1. The primary analysis for rectal swabs among pooled community-level samples will follow the same procedure as specified for nasopharyngeal swabs. We will report confidence intervals for all pair-wise comparisons, and p-values only in the specific gatekeeping order for macrolides. For non-macrolides outcomes, we will report both the unadjusted and FDR-corrected Wilcoxon p-values for the pairwise comparisons.

Antimicrobial Resistance Age-group Spillover Outcomes

Stool samples will be collected from caregivers/guardians of children 1-59 months of age among 20 communities from each arm (a total of 60 communities). The analysis will compare the mean of 24-month results between arms using a permutation p-value approach, and T-test as a validation check. The analysis will be hierarchical and follow a gatekeeping order to control the family-wise error rate to 5% in the following priority 1) Placebo v. 1-59 months Azithromycin, 2) Placebo v. 1-11months Azithromycin and 3) 1-11 months v. 1-59 months Azithromycin.

We will normalize the pooled genetic load reads at the grappe level, and log-transform the data. Then, we will add a pseudo count, a constant computed by normalizing the total non-human reads, to the dataset in order to mitigate observations with zero reads to ensure fold differences can be computed. We will report confidence intervals for all pair-wise comparisons, and p-values only in the specified gatekeeping order for macrolides. For non-macrolides, we will also perform an false discovery rate (FDR) correction on the p-values obtained through the permutation p-value approach.

For nasopharyngeal (NP) samples collected from children 7-12 years old as well as caregivers/guardians of children 1-59 months of age after 2 years of distribution from 20 individuals per community, the NP guardians and youth analysis will be analyzed identical to the guardian stool analysis process detailed above. We will analyze macrolide in a hierrarichal gatekeeping order based on the p-values obtained through permuting the treatment assignment randomly without replacement. As for the non-macrolides, we will report the FDR corrected permuted p-values for the pairwise comparisons.

3.5.3. Missing data

Individuals with missing outcome data are assumed to contribute one half of one phase of person time to the denominator, and no counts to the numerator. Individuals are counted for each phase in which they are confirmed present at the beginning of that phase.

Communities that completely drop out during a phase will be reported, but their measurements in that phase will not contribute to the primary analysis. Earlier inter-census intervals to which communities contributed complete data will be included in the primary analysis.

3.5.4. Additional analyses

Model Assessment

The results of additional regressions will be reported and will be clearly labeled as such and will be sharply distinguished from the primary prespecified outcome. These analyses serve two purposes: (a) assessment of how sensitive the final analytic result is to the prespecified analytic choices, and (b) facilitation of future meta-analytic or review studies. In particular, results will be reported in both absolute and relative scales. Model adequacy will be checked by examination of residuals or other goodness of fit tests as needed. Inadequate model fit will prompt us to report alternative models. As a supplemental, sensitivity analysis, we will estimate permutation *P*-values for each comparison using a randomization test proposed by Simon and Simon, which re-estimates the allocation probabilities within each permutation rather than treating them as fixed [7].

Subgroup Analyses

Pre-specified subgroup analyses for mortality constitute secondary outcomes for the trial. They include:

- Region (Dosso, Tahoua, Maradi, Zinder, Tillaberi). Rationale: examine effect heterogeneity by geographic region.
- Child age (1-11m, 12-24m, 24-59m). Rationale: examine effect heterogeneity by age within each treatment regimen
- Child sex. Rationale: consistent with US NIH reporting of sex as a biological variable.
- Community distance to the nearest Centre de Santé Intégré (CSI). Rationale: previous analysis of the MORDOR trial in Dosso, Niger found larger mortality reductions in communities further from CSIs.
- Number of previous community distributions. Rationale: examine whether effects wane with number of treatments.
- Timing of distribution / seasonality. Rationale: examine effect heterogeneity by season, as mortality could vary by season.
- Other Interventions
    - Participation in the MORDOR trials (Dosso region)
    - Seasonal Malaria Chemoprevention (SMC) in the most recent malaria season if it occurred within the three months prior to the current treatment period. Rationale: examine if there is effect heterogeneity depending on co-delivery with SMC. SMC

coverage. Rationale: examine if there is effect heterogeneity depending on co-delivery with high coverage SMC.

- Trachoma distributions of azithromycin: any distribution received by other programs during the AVENIR trial. Rationale: effect could be attenuated among communities that receive additional treatment. Number of distributions received before and during the AVENIR trial. Rationale: examine effect heterogeneity by the quantity of past and concurrent azithromycin treatments outside of the trial.
- Nutritional status as defined by weight, mid-upper arm circumference (MUAC), height interval. Rationale: determine if there is effect heterogeneity by child nutritional status measured by anthropometry.

Bayesian Analysis

To complement the primary Frequentist analysis, a Bayesian analysis of the trial will be conducted. Ongoing analyses beyond the primary endpoint, reflecting the perpetual nature of the trial, will be conducted using a Bayesian perspective. The details of the Bayesian analyses will be added to a future update of this SAP.

Costing and Cost-Effectiveness

Cost-effectiveness analysis will assess the added net costs, added health outcomes (e.g. deaths and DALYs averted), and the ICER (incremental cost-effectiveness ratio): cost per health outcome achieved by going from no intervention to 1 – 11 months, and then to 1 – 59 months as well as comparing the ICERs for census door to door, CHW door to door and CHW fixed point distribution strategies using cost estimates from the Delivery Trials described below. The health outcomes will be mortality (measured in the trial) and DALYs (calculated from mortality). Cost-effectiveness will be assessed from different perspectives: households, community, government health system, and society. Analyses will produce measures of incremental cost-effectiveness: net cost per dose delivered, per death averted, and net cost per DALY averted. The economic analysis will include two-time frames: the study period of 1 – 4 years and a longer time frame highlighting potential long-term effects such as morbidity, mortality and antimicrobial resistance. All results will be subject to sensitivity analyses: one-way (deterministic), two-way, and multi-way (probabilistic) to assess dependence of outcomes on uncertainties in input values.

The cost-effectiveness analysis will incorporate costs and effectiveness as described above. Determination of cost-effectiveness will be according to a Willingness to Pay criterion, e.g. $150/DALY averted. Costs and DALYs will be discounted at 3% per year. The study will have two time frames: the observed study period (1 – 4 years) and a longer time frame highlighting expected downstream effects and out-of-study effects (see Protocol for further discussion).

Strategies that are more costly but less or equally effective as the next alternative will be considered “dominated”, and removed from consideration, as per best practices. Strategies will be compared by cost from “no intervention” as the base case, going forward by increasing cost. Projected potential benefits in the second time frame may include delayed benefits in the intermediate term based on an observed decay curve, and potentially long-term benefits of the improved health observed during the trial (e.g., nutrition, disease episodes).

To ensure that the model and comparisons are robust, comprehensive sensitivity analyses will be conducted, including one-way, two-way, scenario, threshold and multivariate/stochastic.

3.5.5. Harms

Passive reporting of adverse effects has been used in the past for azithromycin trials. More than 800 million doses have been distributed as part of the trachoma program, and this study will be unlikely to unveil new side effects. We intend to be vigilant, and serious adverse events suspected of being drug-related will be reported to the DSMC within 24 hours of our notification.

We propose no formal statistical analysis of safety events but will report adverse events by arm.

3.5.6. Statistical software

Statistical analysis will be conducted using R (R Foundation for Statistical Computing, Vienna, Austria).

4. References

1. Gamble C, Krishan A, Stocken D, Lewis S, Juszczak E, Doré C, et al. Guidelines for the Content of Statistical Analysis Plans in Clinical Trials. JAMA. 2017;318: 2337–2343. doi:10.1001/jama.2017.18556

2. Keenan JD, Bailey RL, West SK, Arzika AM, Hart J, Weaver J, et al. Azithromycin to Reduce Childhood Mortality in Sub-Saharan Africa. N Engl J Med. 2018;378: 1583–1592. doi:10.1056/NEJMoa1715474

3. Haybittle JL. Repeated assessment of results in clinical trials of cancer treatment. Br J Radiol. 1971;44: 793–797. doi:10.1259/0007-1285-44-526-793

4. Dmitrienko A, D'Agostino RB. Multiplicity Considerations in Clinical Trials. N Engl J Med. 2018;378: 2115–2122. doi:10.1056/NEJMra1709701

5. Benjamini Y, Hochberg Y. Controlling the False Discovery Rate: A Practical and Powerful Approach to Multiple Testing. J R Stat Soc Ser B Methodol. 1995;57: 289–300. doi:10.1111/j.2517-6161.1995.tb02031.x

6. Proctor E, Hiie Silmere, Raghavan R, Hovmand P, Aarons G, Bunger A, et al. Outcomes for implementation research: conceptual distinctions, measurement challenges, and research agenda. Adm Policy Ment Health. 2011;38: 65–76. doi:10.1007/s10477-010-0319-7

7. Simon R, Simon NR. Using randomization tests to preserve type I error with response adaptive and covariate adaptive randomization. Stat Probab Lett. 2011;81: 767–772. doi:10.1016/j.spl.2010.12.018

8. VanderWeele TJ, Knol MJ. A Tutorial on Interaction. Epidemiol Method. 2014;3: 33–72. doi:10.1515/em-2013-0005

5. Revision history

Version	Date	Summary of Changes, Justification, and Timing vis-à-vis key trial events (enrollment completion, interim analyses, unmasking, etc)
1	2020-03-10	• First draft
2	2020-03-30	• Revision to sample size calculations (harmonization across work to date)<br>• Revision to adaptive allocation algorithm text<br>• Revision to analysis details (phase-stratified estimator)
3	2020-05-01	• Harmonized the order of outcomes, and list of secondary outcomes with protocol v1. Added a complete list of pre-specified secondary analyses.<br>• Added language to allow for communities to contribute to the primary outcome if they were missed in a single census but revisited after ~12 months from the last census.
4	2020-07-27	• Added implementation trial to the SAP<br>• Updated sample size and detectable effect calculations based on the revised design, which now includes a mortality trial and a parallel implementation trial.<br>• Described the resistance monitoring as nested within the mortality trial.<br>• Changed the hypothesis testing framework from Bonferroni-Holm to a fixed sequence, hierarchical testing framework to maximize study power.<br>• Added birth history outcome measurement for the implementation trial.
5	2020-07-31	• Added Figure 1 (design overview)
6	2020-08-05	• Added interim analysis (3.5)
7	2020-09-24	Updated the design to reflect DSMC feedback, align with the Protocol:<br>• Increased number of communities selected for mortality trial to 3,350<br>• Delayed start of remainder of communities until 1.5 years after the first enrollment (aligned with interim analysis)<br>• Simplified the comparison of delivery trial approaches to a 2-arm trial and removed mortality as an outcome
8	2020-10-08	Updated outcomes and additional analyses with relevant clarifications for costing and cost-effectiveness.
9	2021-04-06	• Added MORDOR continuation substudies to the outcomes definitions in section 6.1<br>• Updated the multiple testing procedure planned for AMR substudy in section 4.1<br>• Clarified the ITT analysis will only include enrolled grappes
10	2022-02-04	• Added sensitivity analyses to Delivery Trial treatment coverage outcome in section 6.2

11	2022-05-24	• Updated the format to include 3 different sections for the 3 different trials.<br>• Added in the Programmatic Trial section.
12	2022-05-27	• Removed details of CSI clinic visit outcome measurement and analyses until those details can be confirmed vis-à-vis field measurement and logistics.
13	2022-10-21	• Updated wording of person-time calculations for Mortality trial
14	2023-07-24	• Included in the trial overview details of study team masking (membership, timing, rationale), section 2.<br>• Updated Figure 1 and included footnote<br>• Updated the language of tempering roots from 1/3 or 1/4 to 1/4 or 1/6 or 1/8, section 3.2.2.<br>• Removed redundant paragraph for masking in the section of interim Analysis, section 3.2.5<br>• Clarified the timing of the primary analysis, to be exactly 2 years after completion of enrollment, section 3.2.6<br>• Revised Mortality trial primary analysis to align notation with the final adaptive randomization schedule, including the model specification and permutation test, section 3.5.2<br>• Moved Delivery Trial and Programmatic Trial analysis plans to separate SAPs to make the SAP easier to navigate.
15	2023-10-17	• Updated Figure 1 to reflect the final count of communities screened and enrolled in the programmatic trial.
16	2024-09-25	• Updated AMR sample size and power calculations. The lab is going to be processing all 150 communities for the AMR outcomes, so we can include the full 150 communities in the primary AMR analyses, as opposed to the 60 that we listed originally.
17	2025-05-27	• Updated AMR sample size and power calculations for load of genetic determinants resistant to macrolides for nasopharyngeal swabs and rectal swabs (section 3.2.3).<br>• Updated language for AMR primary outcome of nasopharyngeal swabs from “prevalence of genetic determinants” to “load of genetic determinants” given swabs were pooled at the community level (sections 3.3.1 and 3.5.2).<br>• Updated Benjamini-Hochberg FDR for microbiome analysis from 5% only to a range of 5% to 10% (section 3.5.1).<br>• Revised from ANOVA to pairwise analyses for the antimicrobial resistance substudy (section 3.5.2).<br>• Revised AMR substudy analysis methods section to exclude minP stepdown procedure and instead report p-values using the hierarchical gatekeeping framework only (section 3.5.2).<br>• Updated guardian stool analysis to a distinct section: antimicrobial resistance age-group spillover outcomes (section 3.5.2).
