# Supplementary material for: Mass azithromycin distribution and antibiotic resistance in the gut and nasopharynx: a cluster-randomized trial
Source: Nat Med. 2026 Mar 17;32(3):859–68. doi: 10.1038/s41591-026-04217-9 (PMC13004693; doi:10.1038/s41591-026-04217-9)
Supplement: Supplementary file 2 — Reporting Summary [file 41591_2026_4217_MOESM2_ESM.pdf]

nature portfolio

Corresponding author(s): Doan, O'Brien, Lietman

Last updated by author(s): 1/8/2026

Reporting Summary

Nature Portfolio wishes to improve the reproducibility of the work that we publish. This form provides structure for consistency and transparency in reporting. For further information on Nature Portfolio policies, see our Editorial Policies and the Editorial Policy Checklist.

Statistics

For all statistical analyses, confirm that the following items are present in the figure legend, table legend, main text, or Methods section.

n/a	Confirmed	
☐	☒	The exact sample size (*n*) for each experimental group/condition, given as a discrete number and unit of measurement
☐	☒	A statement on whether measurements were taken from distinct samples or whether the same sample was measured repeatedly
☐	☒	The statistical test(s) used AND whether they are one- or two-sided<br>*Only common tests should be described solely by name; describe more complex techniques in the Methods section.*
☐	☒	A description of all covariates tested
☐	☒	A description of any assumptions or corrections, such as tests of normality and adjustment for multiple comparisons
☐	☒	A full description of the statistical parameters including central tendency (e.g. means) or other basic estimates (e.g. regression coefficient) AND variation (e.g. standard deviation) or associated estimates of uncertainty (e.g. confidence intervals)
☐	☒	For null hypothesis testing, the test statistic (e.g. *F*, *t*, *r*) with confidence intervals, effect sizes, degrees of freedom and *P* value noted<br>*Give P values as exact values whenever suitable.*
☒	☐	For Bayesian analysis, information on the choice of priors and Markov chain Monte Carlo settings
☐	☒	For hierarchical and complex designs, identification of the appropriate level for tests and full reporting of outcomes
☒	☐	Estimates of effect sizes (e.g. Cohen's *d*, Pearson's *r*), indicating how they were calculated

Our web collection on statistics for biologists contains articles on many of the points above.

Software and code

Policy information about availability of computer code

Data collection	Dimagi by commcare was used in the field to collect data on participants and consents and sample IDs
Data analysis	Python version 3.13 and R version 4.3.1 was used to generate statistical outputs

For manuscripts utilizing custom algorithms or software that are central to the research but not yet described in published literature, software must be made available to editors and reviewers. We strongly encourage code deposition in a community repository (e.g. GitHub). See the Nature Portfolio guidelines for submitting code & software for further information.

Data

Policy information about availability of data

All manuscripts must include a data availability statement. This statement should provide the following information, where applicable:

- Accession codes, unique identifiers, or web links for publicly available datasets
- A description of any restrictions on data availability
- For clinical datasets or third party data, please ensure that the statement adheres to our policy

Non-host sequencing reads for all pooled samples are available at BioProject ID PRJNA1337442. Limited de-identified individual information is available at DOI: 10.5061/dryad.p8cz8wb48. Requests for more information, beyond the scope of the reported results in this article, are subject to approval by the AVENIR Study Group and must comply with legal and regulatory requirements. Requests can be made to Tom.Lietman@ucsf.edu and/or Kieran.Obrien@ucsf.edu, and will be addressed within 120 days. A data transfer agreement may be required.

Research involving human participants, their data, or biological material

Policy information about studies with human participants or human data. See also policy information about sex, gender (identity/presentation), and sexual orientation and race, ethnicity and racism.

Reporting on sex and gender	No effort was made to collect gender identity information from children enrolled in the study. Their sex was reported by the guardian and subsequently reported in the manuscript.
Reporting on race, ethnicity, or other socially relevant groupings	All subjects were Nigerien. No analyses based on race were performed. Country of origin and race were clearly reported in the manuscript.
Population characteristics	1-59 year-old preschool children in the rural and periurban communities in the Dosso District of Niger. All children weighted at least 3 kg and with no known allergy to macrolide antibiotics. Sex of the participants were reported by their guardians. Age and sex are reported in Table 1 of the manuscript. As the trial was conducted at the community level and all analyses targeted community-effects, both sexes were included in all analyses.
Recruitment	Prior to sample collection, consents were obtained at all levels of the government in Niger. Consents were obtained from caregivers if the children were eligible for the study and recorded electronically. No incentives were given for participation.<br><br>This is a cluster-randomized controlled trial. The randomized design minimizes selection bias and balances both measured and unmeasured baseline characteristics across study arms in expectation. The large sample size increases the likelihood of achieving balance across groups and enhances statistical power. These design features reduce the potential for confounding in comparisons between study arms.
Ethics oversight	Ethics approval was obtained from Comite natioanl Ethique pour la Recherche en Sante in Niger and the institutional review board at the University of California, San Francisco (UCSF). A data and safety monitoring committee independently oversaw trial progress through annual meetings and quarterly progress reports.

Note that full information on the approval of the study protocol must also be provided in the manuscript.

Field-specific reporting

Please select the one below that is the best fit for your research. If you are not sure, read the appropriate sections before making your selection.

☒ Life sciences ☐ Behavioural & social sciences ☐ Ecological, evolutionary & environmental sciences

For a reference copy of the document with all sections, see nature.com/documents/nr-reporting-summary-flat.pdf

Life sciences study design

All studies must disclose on these points even when the disclosure is negative.

Sample size	The primary outcomes were load of macrolide resistance in the gut and nasopharynx. For load of genetic resistance determinants in nasopharyngeal swabs, we estimated that 50 communities per group would provide 80% power to detect an approximately 2-fold difference in any pairwise comparison, assuming an alpha of 0.05 and a standard deviation of the log base 2 read count of 1.88. The standard deviation (SD) of the log read count was derived from the placebo arm of a previous cohort of children in Burkina Faso. For load of genetic resistance determinants in rectal swabs, the inclusion of 50 communities per group provided 80% power to detect a 2.6-fold difference in bacterial reads with resistance determinants between any two arms, assuming a SD of 2.4 on the log scale.
Data exclusions	Samples that were collected in the field, but missing information were not processed for sequencing. All samples meeting criteria were sequenced. And all samples that were sequenced were analyzed. Please also see Figure 2.
Replication	The monitoring of AMR in preschool children receiving azithromycin MDA for childhood mortality currently ongoing in Niger and other countries (Nigeria, Mali, and Burkina Faso).
Randomization	The trial used response-adaptive randomization at the community level. AMR monitoring communities were randomly selected from the group that preceded response-adaptive allocation. Aside from the UCSF biostatistician and data analyst responsible for the randomization, all other study personnel, participants, laboratory personnel, and all field workers responsible for administering and sample collection were masked to treatment allocation. Allocation concealment was achieved by offering the treatment to all children in the community. The appearance and smell of the study drug were similar and packaged identically.
Blinding	Aside from the UCSF biostatistician and data analyst who generated the allocation, investigators, field teams, laboratory personnel, and participants were masked to allocation. Allocation concealment was maintained operationally by offering treatment community-wide; study drug and placebo matched in appearance and smell.

Reporting for specific materials, systems and methods

We require information from authors about some types of materials, experimental systems and methods used in many studies. Here, indicate whether each material, system or method listed is relevant to your study. If you are not sure if a list item applies to your research, read the appropriate section before selecting a response.

Materials & experimental systems

n/a	Involved in the study
☒	☐ Antibodies
☒	☐ Eukaryotic cell lines
☒	☐ Palaeontology and archaeology
☒	☐ Animals and other organisms
☐	☒ Clinical data
☒	☐ Dual use research of concern
☒	☐ Plants

Methods

n/a	Involved in the study
☒	☐ ChIP-seq
☒	☐ Flow cytometry
☒	☐ MRI-based neuroimaging

Clinical data

Policy information about clinical studies

All manuscripts should comply with the ICMJE guidelines for publication of clinical research and a completed CONSORT checklist must be included with all submissions.

Clinical trial registration	NCT04224987
Study protocol	The trial MOP and SAP can be found in the Supplementary Information.
Data collection	Sample collection for this study was from: 05/25/2021 to 8/27/2021 and from 5/20/2023 to 11/23/2023 in Niger.
Outcomes	The primary outcome was pre-defined as load of genetic determinants of resistance to macrolides in the gut and nasopharynx. Relative magnitude of macrolide resistance between treatment arms was determined by the exponentiated mean difference of log-transformed normalized reads. Statistical significance was determined using pairwise Wilcoxon rank-sum tests.<br><br>Secondary outcomes were pre-defined as the load of genetic determinants of resistance to non-macrolides in the gut and nasopharynx. Relative magnitude of macrolide resistance between treatment arms was determined by the exponentiated median difference of log-transformed normalized reads. Statistical significance was also determined using pairwise Wilcoxon rank-sum tests.<br><br>Measures of microbiome diversity, composition, and antibiotic resistance genes were exploratory outcomes.<br><br>Please also see the Statistical Methods section.

Plants

Seed stocks	*Report on the source of all seed stocks or other plant material used. If applicable, state the seed stock centre and catalogue number. If plant specimens were collected from the field, describe the collection location, date and sampling procedures.*
Novel plant genotypes	*Describe the methods by which all novel plant genotypes were produced. This includes those generated by transgenic approaches, gene editing, chemical/radiation-based mutagenesis and hybridization. For transgenic lines, describe the transformation method, the number of independent lines analyzed and the generation upon which experiments were performed. For gene-edited lines, describe the editor used, the endogenous sequence targeted for editing, the targeting guide RNA sequence (if applicable) and how the editor was applied.*
Authentication	*Describe any authentication procedures for each seed stock used or novel genotype generated. Describe any experiments used to assess the effect of a mutation and, where applicable, how potential secondary effects (e.g. second site T-DNA insertions, mosiacism, off-target gene editing) were examined.*
